# Helitwistacenes—Combining Lateral and Longitudinal Helicity Results in Solvent‐Induced Inversion of Circularly Polarized Light

**DOI:** 10.1002/anie.202319318

**Published:** 2024-01-29

**Authors:** Israa Shioukhi, Harikrishna Batchu, Gal Schwartz, Louis Minion, Yinon Deree, Benny Bogoslavsky, Linda J. W. Shimon, Jessica Wade, Roy Hoffman, Matthew J. Fuchter, Gil Markovich, Ori Gidron

**Affiliations:** ^1^ Institute of Chemistry and the Center for Nanoscience and Nanotechnology The Hebrew University of Jerusalem Edmond J. Safra Campus 9190401 Jerusalem Israel; ^2^ School of Chemistry Raymond and Beverly Sackler Faculty of Exact Sciences Tel Aviv University 6997801 Tel Aviv Israel; ^3^ Molecular Sciences Research Hub Department of Chemistry Imperial College London White City Campus, 82 Wood Lane W12 0BZ London U.K.; ^4^ Chemical Research Support Unit Weizmann Institute of Science 76100 Rehovot Israel; ^5^ Department of Materials Royal School of Mines Imperial College London SW7 2AZ London U.K.

**Keywords:** Helicenes, Twistacenes, Chirality, Circular Dichroism, Circularly Polarized Luminescence

## Abstract

Helicity is expressed differently in ortho‐ and para‐fused acenes—helicenes and twistacenes, respectively. While the extent of helicity is constant in helicenes, it can be tuned in twistacenes, and the handedness of flexible twistacenes is often determined by more rigid helicenes. Here, we combine helicenes with rigid twistacenes consisting of a tunable degree of twisting, forming helitwistacenes. While the X‐ray structures reveal that the connection does not affect the helicity of each moiety, their electronic circular dichroism (ECD) and circularly polarized luminescence (CPL) spectra are strongly affected by the helicity of the twistacene unit, resulting in solvent‐induced sign inversion. ROESY NMR and TD‐DFT calculations support this observation, which is explained by differences in the relative orientation of the helicene and twistacene moieties.

## Graphical abstract

Combining helicenes with twistacenes, in which the extent of twisting can be tuned, has led to the new family of helitwistacenes. The combination of lateral and longitudinal helicity results in polarity‐induced and twisting‐induced sign inversion of both the electronic circular dichroism and circularly polarized luminescence signals. This sign inversion could be explained by differences in the relative orientation of the helicene and twistacene moieties.

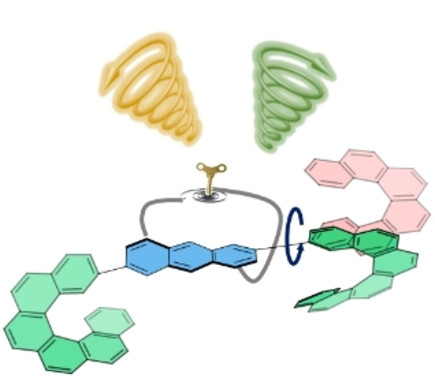

## Introduction

Introducing one or more stereogenic elements to aromatic materials endows them with new electronic, (chiro)optical and magnetic properties that often form the basis for new technologies. For example, chiral materials exhibiting circularly polarized luminescence (CPL) are applied in 3D displays, encryption, and optoelectronic devices.^[1]^ Perhaps the most common structural motif for introducing chirality to polyaromatic materials is by *ortho*‐fusing benzenes to create helicenes.[^2^, ^3^] Helicene‐containing materials are widely explored for application in polarized light absorbing and emitting devices.[^4^, ^5^] However, their para‐fused analogs, twistacenes, are significantly less explored.^[6]^ The combination of helicenes with twistacenes was initially suggested by Pascal, for the construction of an all‐carbon Möbius nanobelt,^[7]^ who later introduced a hybrid helicene and twistacene moiety, in the form of hairpin furans.^[8]^ Recently, both the Nuckolls and Mateo‐Alonso groups directed the handedness of flexible twisted nanoribbons by combining these two structural building blocks.[^9^, ^10^, ^11^] Nazario et al. used helicene scaffold to induce chirality and twist to large nanographenes.[^12^, ^13^]

**Figure 1:**
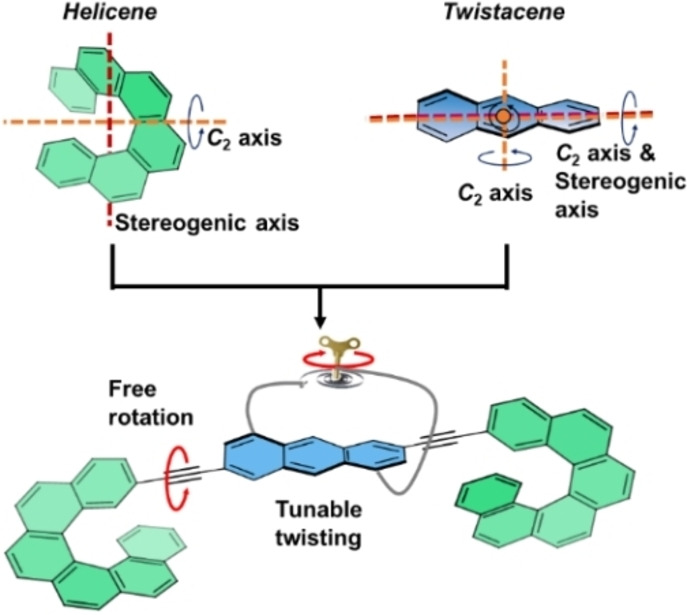
Top: Schematic representation of [6]helicene and twistacene, showing the C2 axes (orange) and stereogenic axes (red). Bottom: Combining helicenes and tethered twistacene with alkyne spacers.

In helicenes, the stereogenic axis is perpendicular to the main *C*_2_ axis (Figure 1, top left), whereas in twistacenes, the stereogenic axis is also the main *C*_2_ axis, crossing through the aromatic backbone (Figure 1, top right). Helical chirality is therefore expressed in lateral helicity in helicenes and longitudinal helicity in twistacenes.^[14]^ Whereas the pitch is constant in helicenes (whose backbone undergoes a full turn every seven rings), backbone twisting in twistacenes can be controlled by the steric parameters of the side groups.^[7]^ Obtaining twistacenes in their enantiopure form is challenging because their conformational flexibility produces racemic mixtures.^[15]^ We therefore applied cyclophane chemistry,^[16]^ to introduce helically‐locked twistacenes, whose conformational stability enables them to be separated into their enantiopure forms.^[17]^ We found that we can tune the electronic, chiro(optical), and magnetic properties by controlling the acene twist.[^18^, ^19^, ^20^] Absorbing tethered twistacenes to different surfaces also affect the extent of chiral induced spin selectivity (CISS).^[21]^

The intensity of the chiroptical properties is expressed by the scalar product of the magnetic and electric dipole moments.^[22]^ In some cases, particularly when two chromophores are connected by a single bond, conformational changes can increase or decrease their chiroptical properties, as shown in their electronic circular dichroism (ECD) and CPL spectra.[^23^, ^24^, ^25^, ^26^] For helicenes, conformational changes can be induced by environmental triggers, thus generating pH, redox and photo switching.^[27]^ In some cases, these conformational changes lead to sign inversion of the polarized light absorption and emission. For example, binaphthyl scaffolds exhibit solvent‐dependent sign‐reversal of CPL,[^28^, ^29^, ^30^, ^31^] helicenes display CPL inversion upon substitution^[32]^ and upon protonation,[^26^, ^33^] and crown ether‐based sensors show reversible switching upon cation coordination.^[34]^ Such chiral switches can be applied as light modulators,^[35]^ as it was observed that reversing the current on azahelicene‐doped OLED reverses the CPL signal.^[36]^ The Mori group demonstrated that the relative orientations of helicenes significantly affects their chiroptical response.^[37]^ However, in all these cases, the extent of backbone twisting was not controlled.

Here, we combine twistacenes whose twist is tunable by manipulating the length of their end‐to‐end tether with helicenes via a flexible triple bond that enables conformational modification (Figure 1, bottom). Solid‐state X‐ray analysis of their structures shows that the helicenes adopt a *syn* conformation. Sign inversion of both ECD and CPL occurs upon modifying the solvent polarity. In addition, the same enantiomer produces opposite ECD signals upon tether extension. Time‐dependent density‐functional theory (TD‐DFT) calculations and ROESY NMR measurements support this trend, and indicate that these effects originate from the different relative orientations of the twistacene and helicene moieties, which in turn invert the magnetic dipole moment.

## Results and Discussion

**Scheme 1:**
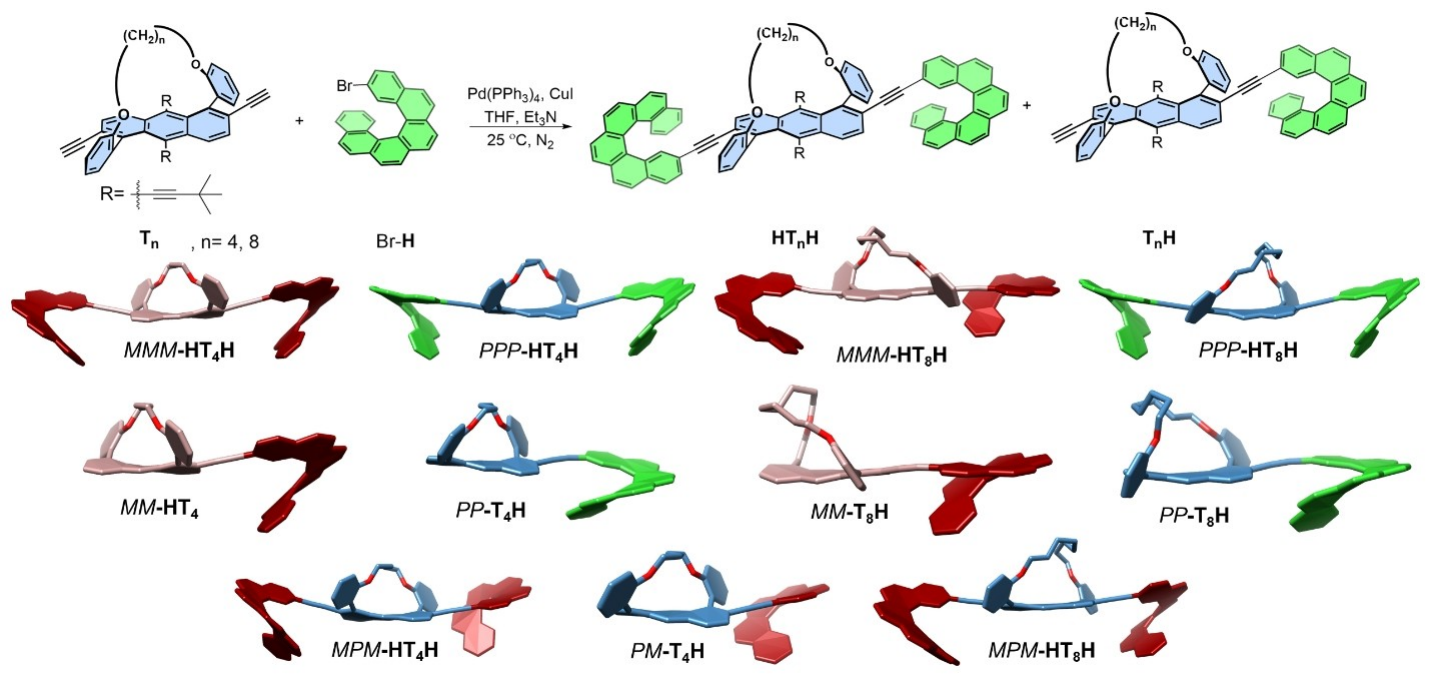
Top: Synthetic pathway for the preparation of helicene‐twistacenes (**T_n_H**) and helicene‐twistacene‐helicenes (**HT_n_H**). Et_3_N, triethylamine; PPh_3_, triphenylphosphine; THF, tetrahydrofuran. Middle and bottom: calculated (B3LYP/6‐31G(d)) structures of **T_4_H** (*MM*; 13 %, *PP*; 26, *PM*; 13 %), **HT_4_H** (*MMM*; 13 %, *PPP*; 14 %, *MPM*; 17 %), **T_8_H** (*MM*; 13 %, *PP*; 16 %) and **HT_8_H** (*MMM*; 11 %, *PPP*; 17 %, *MPM*; 15 %). Maroon/red: *P* handedness, blue/green: *M* handedness.

For the synthesis of helitwistacenes, tethered twistacenes **T_4_** and **T_8_**, bearing acetylene spacers at the 2,6‐positions,^[38]^ were coupled to bromo‐[6]helicene^[39]^ (**H**) using Sonogashira coupling as depicted in Figure 1 and Scheme 1. The twistacene moieties, referred to here as **T_4_** and **T_8_**, are sterically locked into an end‐to‐end twist by a butyl or octyl tether, respectively, that is diagonally attached at the 1,5 positions. The major products were a mixture of singly coupled helicene‐twistacenes (**T_4_H** and **T_8_H**) and doubly coupled helicene‐twistacene‐helicenes (**HT_4_H** and **HT_8_H**), in which each helicene and twistacene could have *M* or *P* helicity. Thus, synthesis produced helicene‐twistacenes *P,M*‐**T_4_H**, *M,M*‐**T_4_H** and *M,M*‐**T_8_H** *P,P*‐**T_4_H** and *P,P*‐**T_4_H** as well as helicene‐twistacene‐helicene *PPP‐* **HT_4_H** and *PPP‐* **HT_8_H**, *MMM‐* **HT_4_H** and *MMM‐* **HT_8_H**, and *MPM*‐**HT_4_H** and *MPM*‐**HT_8_H** (Scheme 1, bottom). The products were characterized using mass and NMR spectroscopy (see Supporting Information).

**Figure 2:**
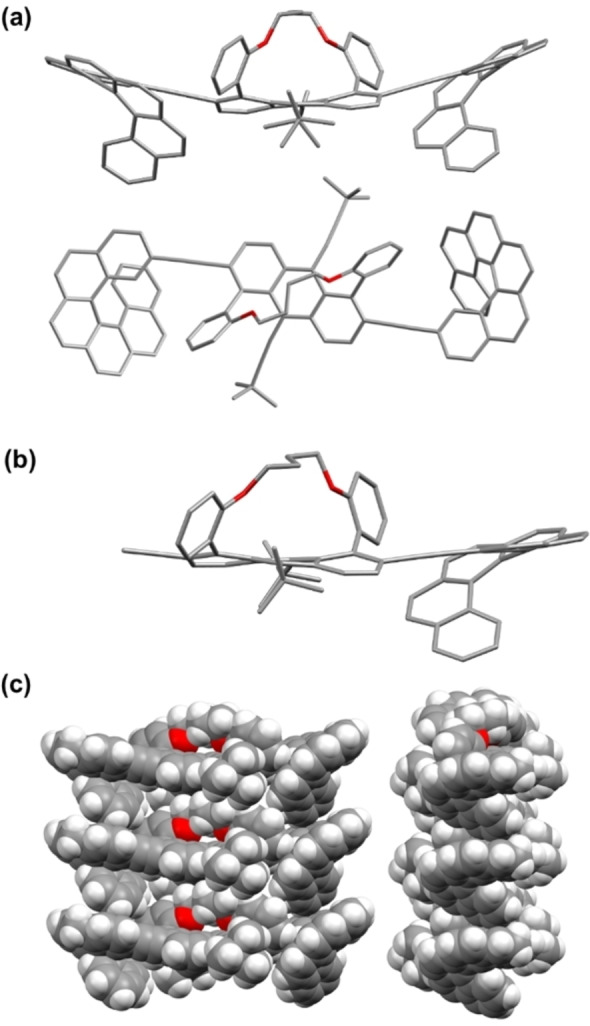
X‐ray structures. (a) side view and top view of *MPM*‐**HT_4_H**. (b) Side view of *MP*‐**T_4_H**. Hydrogen atoms are omitted for clarity. (c) Packing of *MPM*‐**HT_4_H** (spacefill).

To obtain further structural details, *MPM*‐**HT_4_H** and *MP*‐**T_4_H** were crystallized from a hexane/dichloromethane mixture (Figure 2).^[40]^ Connecting the diagonal 1,5 positions of the twistacene moiety using a butyl tether induces end‐to‐end twisting in the anthracene moiety of 33° in *MPM*‐**HT_4_H** and of 29° in *MP*‐**T4H**. This is in line with the previously‐observed 31° twisting for **T_4_** in contrast to 4° for **T_8_**,^[38]^ and with the calculated twisting angle for *MPM*‐**HT_4_H** of 33°. As expected, coupling to helicene did not affect the degree of twistacene twist, which is mostly determined by tether length.

For *MPM*‐**HT_4_H** in the solid state, the two helicenes orient *syn* with respect to each other, angled away from the tether, creating a C‐shaped structural motif. The small dihedral angle (23°) between the twistacene and the helicene indicates good π orbital overlap. A nearly identical orientation is observed for the *MP*‐**T_4_H** structure, which also has a 23° dihedral angle. This can be explained by a combination of increased π overlap between the twistacene and helicene moieties and tight intermolecular packing (Figure 2c). The slight twist results from the H‐π interactions, with distances of 2.7 Å between neighboring helicenes. Both structures exhibit a columnar arrangement, despite the alkyl‐tether that was expected to prohibit close packing. It is interesting to note that previous structures of tethered twistacenes did not display columnar packing, which could stem from the fact that the current structures are the first reported for enantiopure tethered twistacenes.

**Figure 3:**
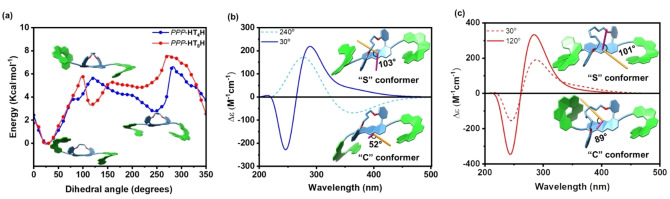
(a) Normalized UV/Vis absorption spectra of twistacene (**T_4_**, blue line), helicene (**H**, green line) and *PPP*‐**HT_4_H** (red line) and the calculated spectrum (pink). (b) The natural transition orbitals involved with labelled transitions (yellow→blue represents the difference in electron density for S_0_→S_n_). (c) ECD spectra of helitwistacenes. All experimental spectra were measured in tetrahydrofuran.

The absorption spectra for *PPP*‐**HT_4_H** is given as representative in Figure 3a (the spectra for the entire series is reported in section S4 in the Supporting Information). The spectra display a well‐structured transition between 410–500 nm, three overlapping bands between 360–380 nm, and a broader transition at ~320 nm. For better assignment of each transition, the spectrum was compared to **T_4_** (blue line) and [6]helicene (**H**, green line). The lowest energy transition can be assigned to the *p*‐band of acenes, bathochromically shifted by ca. 20 nm compared to **T_4_**. The bands at 360–380 nm correspond to the *β*‐band of helicene, bathochromically‐shifted by ca. 40 nm compared to [6]helicene, overlapping with the *β*‐band for twistacene. The calculated absorption spectrum matches the experimental one, and Natural Transition Orbitals (NTO) analysis is in agreement with the expected contributions from the twistacene acetylene and helicene moieties. The bathochromic shift for the *p*‐band is explained by the extension of conjugation to the acetylene moiety (Figure 3b, top). It is interesting to note that this transition consists of helical orbital density around the acetylene bond, and such electrohelicity is often related to intense chiroptical properties. The transition at ~320 is assigned to the *β*‐band of the twistacene moiety. The calculated spectrum for **HT_4_H** corresponds to the experimental spectra (Figure 3a, bottom), and the orbital contributions for the main transitions support the above‐mentioned assignment (see Supporting Information).

Examining the ECD spectra for all the synthesized **HT_4_H** and **HT_8_H** (Figure 3c), several differences stand out between the lowest energy band (410–500 nm) and the higher‐energy *β*‐bands (320–400 nm): (1) Although the sign of the higher‐energy bands is determined by the handedness of the helicene, the sign of the lowest‐energy band does not follow the handedness of the twistacene in the same manner. (2) The intensity of the higher energy bands is relatively similar for all compounds, whereas the intensity of the lowest energy band varies significantly, from −15 (*MPM*‐**HT_8_H)** to −96 (*MPM*‐**HT_4_H)** cm^−1^ M^−1^.

Perhaps the most striking observation emerges from comparing the ECD spectra of the lowest energy bands for *PPP*‐**HT_4_H** and *PPP*‐**HT_8_H** (Figure 3c (inset)). Although both are composed of helicene and twistacene moieties with the same *P‐*handedness, the ECD signals of their lowest transitions have opposite signs. To the best of our knowledge, such sign reversal for the absorption of circularly polarized light by the same enantiomer is unprecedented for helicenes or twistacenes.

**Figure 4:**
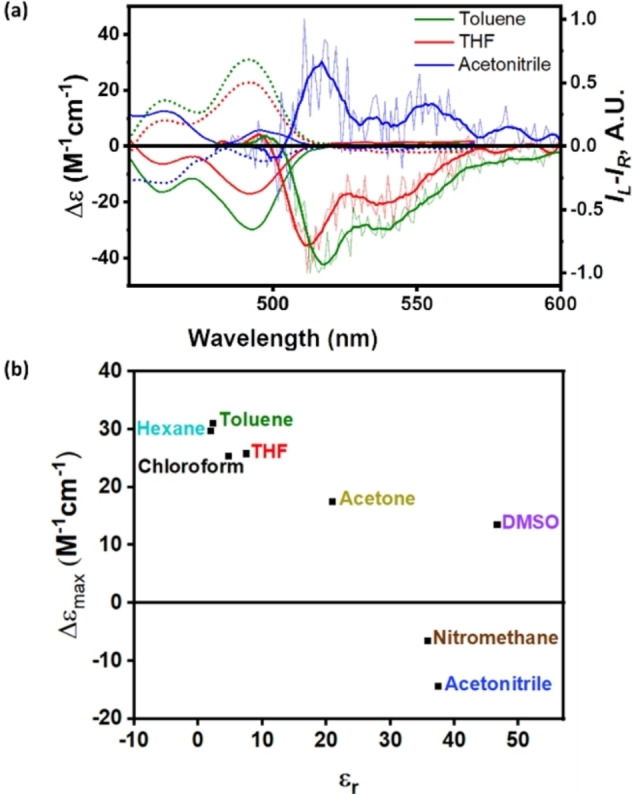
(a) ECD of *PPP*‐**HT_4_H** and *MMM*‐**HT_4_H** and CPL of *PPP*‐**HT_4_H** in tetrahydrofuran (red), acetonitrile (blue) and toluene (green). The smoothed CPL spectrum is displayed in bold and the original data are displayed by thin lines. (b) The maximal intensity of the S_0_→S_1_ transition vs. solvent polarity (dielectric constant) of *MMM*‐**HT_4_H** at 493 nm.

While ECD was previously reported to be affected by environmental factors such as solvent polarity,^[31]^ this phenomenon has not been reported for twisted acenes. We therefore attempted to examine the effect of solvents on our helitwistacenes. Figure 4a presents the ECD spectra of *PPP*‐**HT_4_H** and *MMM*‐**HT_4_H** in three different solvents; tetrahydrofuran, toluene and acetonitrile. Whereas the signal for *PPP*‐**HT_4_H** is negative in both toluene and tetrahydrofuran, the sign inverts upon dissolving it in the more polar acetonitrile, with the *MMM*‐**HT_4_H** enantiomer displaying the opposite effect. It is interesting to note that the other higher energy transitions are not affected in the same manner, i.e., the sign does not invert with increasing solvent polarity. Extending the study to different solvents, a clear correlation can be observed between the solvent polarity and Δϵ (Figure 4b), with complete sign inversion observed for the most polar solvent. DMSO is the only outlier, which indicates that alongside solvent polarity, a specific noncovalent interaction may be involved.

Since sign‐inversion was observed for the S_0_→S_1_ transition, we expected that the CPL spectra, which are composed exclusively of the S_1_→S_0_, should also behave in the same manner (invert with increasing solvent polarity). Indeed, the CPL in helitwistacenes strongly depends on solvent polarity (Figure 4, right). The anisotropy factor *g*_lum_ is positive for *PPP*‐**HT_4_H** in acetonitrile (g_lum_=3×10^−4^) and negative in THF and toluene (g_lum_=−4×10^−4^ for both). Compared with the ECD spectra, same sign and small Stokes shift (0.15 eV) indicate similar conformation in the excited state. The opposite trend is observed for the *MMM* enantiomer, further verifying this phenomenon. In contrast to the abovementioned solvent dependency observed for *PPP*‐**HT_4_H**, solvent‐induced ECD and CPL of *PPP*‐**HT_8_H** did not result in the same sign inversion, but only in the reduction of the intensity upon increasing the solvent polarity (Figure S58). We note that the CPL brightness (B_CPL_) is in the range of 2 M^−1^ cm^−1^ for both *PPP*‐**HT_4_H** and *PPP*‐**HT_8_H**, which is the typical range for helicenes.^[41]^ An analysis of the Lippert–Mataga plot, indicates a significant deviation from linearity, which serves as an indication that the conformational differences stem from the solvent polarity. The ratio g_lum_/g_abs_ is ca. 0.5, which corresponds to previously reported twistacenes and helicenes.[^20^, ^22^]

**Figure 5:**
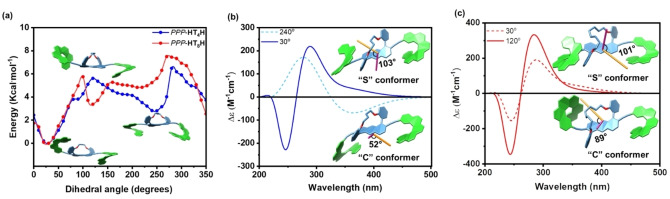
(a) Calculated (B3LYP‐GD3/6‐31G(d)) torsional energy of *PPP*‐**HT_4_H** (blue) and *PPP*‐**HT_8_H** (red). Calculated (TD‐DFT/CAM‐B3LYP/6‐31G(d)) ECD for *PPP*‐**HT_4_H** (b) and *PPP*‐**HT_8_H** (c) at their local and global minima. Magnetic and electric transition dipole moment (MTDM and ETDM) vectors are represented by purple and orange lines, respectively, for each conformer with their relative angles.

To understand the reason for the ECD spectral sensitivity of *PPP*‐**HT_4_H**, we performed DFT and TD‐DFT calculations for different conformations of *PPP*‐**HT_4_H** and *PPP*‐**HT_8_H**. As both the helicene and twistacene units are fairly rigid, the only significant degree of freedom is expressed in their relative orientations, which are determined by free rotation around the acetylene bond. We therefore performed a scan around this bond in both *PPP*‐**HT_4_H** and *PPP*‐**HT_8_H** (Figure 5a). Both *PPP*‐**HT_4_H** and *PPP*‐**HT_8_H** have a global minimum at a relative helicene‐twistacene dihedral angle of 30° when in the “S” conformation, that is, when the two *P* helicenes are *anti* with respect to each other. The difference between *PPP*‐**HT_4_H** and *PPP*‐**HT_8_H** is evident in their local minima: *PPP*‐**HT_4_H** (blue trace) exhibits a local minimum at 240° (“C” conformer) in which the two helicenes are angled away from the tether, similarly to their X‐ray conformation (Figure 2a). In contrast, *PPP*‐**HT_8_H** shows a local minimum at 120° (Figure 5a). Figure 5b shows the calculated (TD‐DFT) ECD spectra of the two minima mentioned above. Similar to the experimental observation, the lower energy transition (~420 nm) is most affected by the conformational changes, being negative for a 240° orientation (being the local minimum for *PPP*‐**HT_4_H**) and positive for a 30° orientation (being the global minimum). The ECD or CPL intensity of a given transition is proportional to its rotational strength (R): R=|ETDM||MTDM| ⋅ cos (Θ), where ETDM and MTDM are the electric and magnetic transition dipole moment vectors, respectively, and Θ is the angle between them. To understand why the lowest energy band is affected to the largest extent by the relative orientations of the helicene and twistacene moieties, we plotted the ETDM (purple) and MTDM (orange) vectors at both 240° and 30° (Figures 5b and 5c). The different values of Θ, namely Θ=103° for a 30° orientation and Θ=52° for 240° orientation, result in sign reversal for cosine Θ (and therefore an opposite sign of R) and account for the sign reversal observed in the ECD spectra. In contrast, in the case of *PPP*‐**HT_8_H**, the 89° between the ETDM and MTDM observed for the local minima of 120° does not expect to produce a significant contribution to the ECD (as the cosine value is very small). We can therefore conclude that (i) the lowest energy band is strongly affected by conformation, which can explain the sensitivity of this transition to environmental factors such as solvent polarity, and (ii) *PPP*‐**HT_4_H** and *PPP*‐**HT_8_H** exhibit different local minima, each resulting in an opposite sign for the lowest energy transition in the ECD spectra.

**Figure 6:**
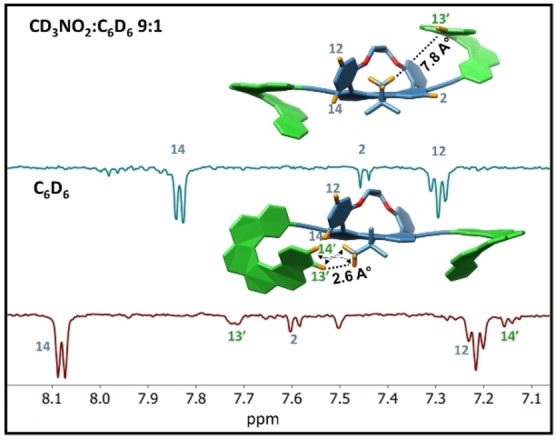
ROESY NMR spectra recorded in CD_3_NO_2_:C_6_D_6_ 9 : 1 (blue trace) and C_6_D_6_ (red trace) of *PPP*‐**HT_4_H**.

The conformational difference in different solvents is also supported by the ^1^H NMR of *PPP*‐**HT_4_H**: Upon increasing the CD_3_CN:CDCl_3_ ratio from 1 : 0 to 0.5 : 0.5, helicene protons located in the vicinity of the acetylene bond or close to the bridge are more affected compared to distant protons (for full discussion, see section S7 in the Supporting Information for details). ROESY NMR in different solvents provided an additional indication for the solvent‐dependent conformational changes: in non‐polar benzene, we observe a correlation between the *tert*‐butyl and protons 13’ and 14’ located on the helicene moiety, as expected for the “C” conformer (Figure 6, red trace). In contrast, in more polar solvent mixture (CD_3_NO_2_ : C_6_D_6_ 9 : 1), this correlation is not observed, indicating that the helicene moieties are facing towards the bridge (Figure 6, blue trace).

## Conclusion

In summary, we have introduced helitwistacenes as molecules that consist of acenes with both lateral and longitudinal helicity. The relative orientations of the constituent units strongly affect their chiroptical properties. Helitwistacenes with the same helicity but different twists display opposite ECD signals for the lowest energy transition. In addition, modification of solvent polarity inverts both the ECD and CPL signals. TD‐DFT calculations and ROESY NMR support these observations, showing different local minima for different degrees of twisting, and strong sensitivity of the lowest energy transition to conformational changes, specifically, the relative orientation between the helicene and twistacene units. This work demonstrates the potential of combining helicenes and twistacenes to develop sensitive chiroptical responsive materials and building units for macrocycles with Möbius aromaticity.

## Supporting Information

The authors have cited additional references within the Supporting Information.

## Conflict of interests

The authors declare no conflict of interest.

1

## Supporting information

As a service to our authors and readers, this journal provides supporting information supplied by the authors. Such materials are peer reviewed and may be re‐organized for online delivery, but are not copy‐edited or typeset. Technical support issues arising from supporting information (other than missing files) should be addressed to the authors.

Supporting Information

Supporting Information

Supporting Information

## Data Availability

The data that support the findings of this study are available from the corresponding author upon reasonable request.
