# Supplementary material for: Helitwistacenes—Combining Lateral and Longitudinal Helicity Results in Solvent‐Induced Inversion of Circularly Polarized Light
Source: Angew Chem Int Ed Engl. 2024 Jan 29;63(11):e202319318. doi: 10.1002/anie.202319318 (PMC11497310; doi:10.1002/anie.202319318)
Supplement: Supplementary file 1 — Supporting Information [file ANIE-63-e202319318-s001.pdf]

Angewandte Chemie
Eine Zeitschrift der Gesellschaft Deutscher Chemiker

Supporting Information

Helitwistacenes—Combining Lateral and Longitudinal Helicity Results in Solvent-Induced Inversion of Circularly Polarized Light

*I. Shioukhi, H. Batchu, G. Schwartz, L. Minion, Y. Deree, B. Bogoslavsky, L. J. W. Shimon, J. Wade, R. Hoffman, M. J. Fuchter, G. Markovich, O. Gidron**

Supporting Information

Helitwistacenes – Combining Lateral and Longitudinal Helicity Results in Solvent-Induced Inversion of Circularly Polarized Light.

Israa Shioukhi[1], Harikrishna Batchu[1], Gal Schwartz[2], Louis Minion[3], Yinon Deree[1], Benny Bogoslavsky[1], Linda J. W. Shimon[4], Jessica Wade[3,5], Roy Hoffman[1], Matthew J. Fuchter[3], Gil Markovich[2] and Ori Gidron[1*]

[1]Institute of Chemistry and the Center for Nanoscience and Nanotechnology, The Hebrew University of Jerusalem, Edmond J. Safra Campus, 9190401, Jerusalem, Israel. Email: ori.gidron@mail.huji.ac.il. [2]School of Chemistry, Raymond and Beverly Sackler Faculty of Exact Sciences, Tel Aviv University, Tel Aviv 6997801, Israel. [3]Molecular Sciences Research Hub, Department of Chemistry, Imperial College London, White City Campus, 82 Wood Lane, London W12 0BZ, U.K. [4]Chemical Research Support Unit, Weizmann Institute of Science, Rehovot 76100, Israel. [5]Department of Materials, Royal School of Mines, Imperial College London, London, SW7 2AZ, U.K.

Contents

S1 General 3

S2 Synthesis 6

S3 Characterization 7

S3.1 NMR 7

S3.2 Chiral HPLC separation of racemic twisted acenes and 2-bromo[6]helicene 34

S3.3 Mass spectroscopy: MALDI and LCMS 37

S4 Photophysical properties 48

S4.1 UV-vis absorption spectra 48

S4.1.1 UV-vis absorption spectra of ***MMM***-HT_4H and ***PPP***-HT_8H in different solvents. 49

S4.2 ECD spectra 51

S4.2.1 ECD spectra of helitwistacene molecules in different solvents 52

S4.2.2 The effect of solvents viscosity on the ECD. 55

S4.2.3 ECD spectra of ***PPP-*** HT_4H molecules in different temperatures. 56

S4.3 Steady-state fluorescence spectra 57

S4.3.1 Steady-state fluorescence spectra of *PPP*-**HT_4H** and *PPP*-**HT_8H** in different solvents 58

S4.3.2 Lippert-Mataga plot of (a) *PPP*-**HT_4H** and (b) *PPP*-**HT_8H**. 58

S4.4 Excitation spectra 61

S4.5 Circularly polarized luminescence (CPL). 62

S4.6 Quantum yields and CPL brightness factor of helitwistacenes molecules. 63

S4.7 Fluorescence lifetime (τ_f) 64

S5 Single crystal X-ray diffraction crystallography (SCXRD) 65

S6 Computational details 68

S6.1 Calculated structures of the synthesized molecules 69

S6.2 Calculated UV-vis absorption spectrum and CD spectrum of the synthesized molecules 71

S6.3 Scanning potential energy surface 72

S7 NMR analysis in different solvents 75

S7.1 NMR analysis of *PPP*-**HT_4H** in different solvents 75

S7.2 NMR analysis of *PPP*-**HT_8H** in different solvents 78

S8 Supplementary References 79

S1 General

Commercially available reagents and chemicals were used without further purification unless otherwise stated. Twistacenes and 2-Bromo[6]Helicene were synthesized according to previously published methods.[1,2]

Flash chromatography (FC) was performed using CombiFlash SiO_2 columns. Chiral HPLC separations were performed with a Chiralpak® IG semi-preparative column and CHIRALPAK® IB-N (250 × 4.6 mm / 5µm) preparative columns, with hexane/dichloromethane as eluent.

^{1}H and ^{13}C NMR spectra were recorded in solution on a Bruker-Neo 400 MHz and 500 MHz spectrometers using the ^{1}H signal of tetramethylsilane (TMS) or the residual solvent peak that had been previously calibrated [3] to TMS as the external standard. ^{13}C-NMR spectra were referenced to the ^{1}H frequency multiplied by the standard factor of 0.25145020.[4] ^{13}C-NMR spectra were ^{1}H decoupled. The spectra were recorded using chloroform-*d*, CD_3CN, C_6D_6 and CD_3NO_2. Chemical shifts (δ) are expressed in ppm.

COSY spectra were phase-sensitive and gradient-enhanced and double-quantum filtered. ^{1}H-^{13}C Heteronuclear single-quantum coherence (HSQC) spectra were phase-sensitive and gradient-enhanced using a double INEPT transfer with sensitivity improvement. They included multiplicity editing using 180° pulses with matched adiabatic sweep.[5] ^{1}H-^{13}C Heteronuclear multiple-bond coherence (HMBC) spectra were gradient-enhanced using zero- and double-quantum coherence. They are phase-sensitive in the evolution (*f*1) dimension and employ a three-fold low-pass *J*-filter to suppress one-bond correlations.[6] The size of the molecules studied have tumbling rates in solution that lead to NOE correlations near zero. As a result, ROE correlations are stronger that NOE correlations. Selective 1D-ROE (known as selective ROESY) measurements were used in this work.[7]

UV-vis absorption spectra were recorded with an Agilent Cary-5000 spectrophotometer. The spectra were measured using a quartz cuvette (1 cm) at 25 °C. The absorption wavelengths are reported in nm with the extinction coefficient ε ($M^{-1}cm^{-1}$) in brackets.

Steady state fluorescence measurements were performed on a HORIBA JOBIN YVON Fluoromax-4 spectrofluorometer with the excitation/emission geometry at right angles. Fluorescence quantum yields (Θ_f) were determined using an integrating sphere. The lifetimes of the excited species were measured using an NL-C2 Pulsed Diode Controller NanoLED light source

with time-correlated single proton counting (TSCPC) Controller DeltaHub (HORIBA), referenced against colloidal Ludox solution (50 wt. % solution in water) obtained from Aldrich.

Electronic Circular Dichroism (ECD) spectra were recorded on a MOS-500 spectrophotometer from BioLogic Science Instruments.

High resolution mass spectra were measured on a HR Q-TOF LCMS and Waters Micromass GCT_Premier Mass Spectrometer using ESI MALDI-TOFMS spectra were acquired using an MALDI-TOF/TOF autoflex speed mass spectrometer (Bruker Daltonilk GmbH, Bremen, Germany) equipped with a smartbeam-II solid-state laser (modified Nd:YAG laser) λ = 355 nm. The instrument was operated in positive ion, reflectron mode. The accelerating voltage was 21.0 kV. The delay time was 130 ns. Laser fluence were optimized for each sample. The laser was fired at a frequency of 2 kHz and spectra were accumulated in multiples of 500 laser shots to achieve 1500 shots in total. Sample preparation: 2-[(2E)-3-(4-tert-Butylphenyl)-2-methylprop2-enylidene] malononitrile (DCTB) matrix solutions were made to a concentration of 20 mg/mL in dichloromethane (DCM). Sample solutions were made to an approximate concentration of 5 mg/mL in DCM. Sample and matrix solutions were premixed at a ratio of 1:9 or 1:40 (v/v). A volume of 0.5 µL of this mixture was deposited onto a MALDI steel target plate. After evaporation of the solvent, the target was inserted into the mass spectrometer.

Experimental setup for CPL measurements

All measurements were performed in a home-built CPL system using a photoelastic modulator and lock-in detection.[8] The samples were excited using a 395 nm diode laser (Vortran Stradus, 100 mW). Light emitted from the samples was collected at 90° to the excitation, passed through the photoelastic modulator, linear polarizer and monochromator, and detected by a photomultiplier. The laser light passed through a shortpass filter with a cutoff wavelength of 450 nm and the emitted light passed through a longpass filter with a cutoff wavelength of 450 nm at the entrance to the monochromator. A fused silica cuvette of 1 cm optical path length was used for all measurements. Spectral smoothing was obtained by Savitzky-Golay method (15 points, 2nd order) applied in OriginPro 2022 software

Single crystal X-ray diffraction crystallography (SCXRD)

Single crystals of *MP*-**T$_4$H** and *MPM*-**HT$_4$H** were obtained from a mixture of dichloromethane/hexane by the slow evaporation method. The system was controlled by a Pentium-based PC running the SMART software package.[9] Data were collected at room temperature using Mo-Kα radiation (λ=0.71073 Å). Immediately after collection, the raw data frames were subjected to integration and reduction by the SAINT program package.[10] The structure was solved and refined by the SHELXTL software package.[11]

X-Ray diffraction data sets and solution for crystals of *MPM*-**HT$_4$H** were collected by mounting a single crystal on a MiTeGen MicroLoops E™, then using a XtaLAB Synergy-S, Single source at offset/far, HyPix diffractometer equipped with oxford Cryostream 800 operating at T = 100.0(1) K. Data were measured using MoKα radiation. Using Olex2, the structure was solved with the SHELXT structure solution program using Intrinsic Phasing and refined with the SHELXL refinement package using Least Squares minimization.

S2 Synthesis

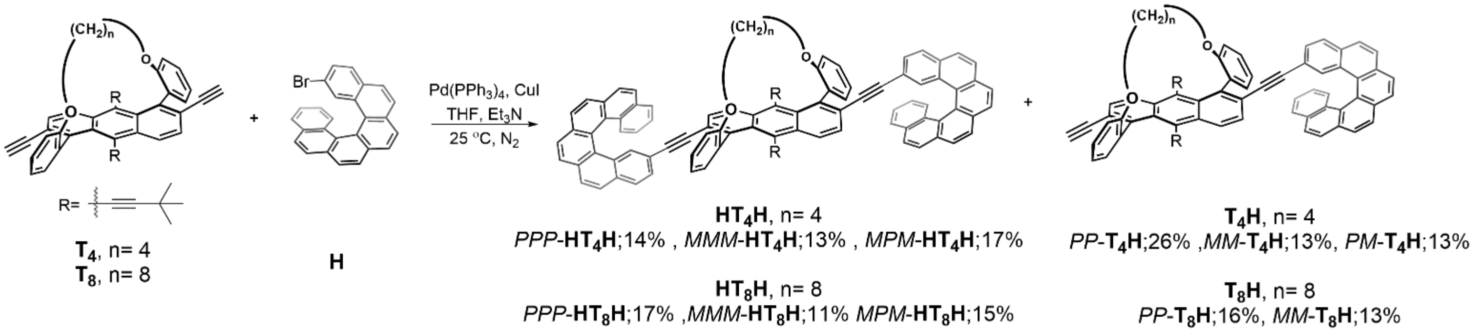

General procedure for the synthesis of helitwistacene molecules:

Under an inert atmosphere, enantiopure twistacene (0.078 mmol), enantiopure 2-bromo[6]Helicene (0.235 mmol), $Pd(PPh_3)_4$ (4.5 mg, 5% mol, 0.0039 mmol), and CuI (1.5 mg, 10% mol, 0.0078 mmol) were added to an oven-dried one-necked round bottomed flask equipped with a magnetic stirrer. A mixture (4 mL) of dry trimethylamine and tetrahydrofuran (THF; 1:1) was added to the previously mixed reagents. The reaction mixture was stirred for 3 days at room temperature. The solvents were evaporated and the reaction mixture was loaded into a silica gel column. A 10% of a mixture of ethyl acetate in hexane or a 20% mixture of ethyl acetate in hexane were used to obtain singly coupled helicene-twistacenes (**T_4H** and **T_8H)** or doubly coupled helicene-twistacene-helicene (**HT_4H** and **HT_8H**), respectively, in which the twistacene was sterically locked into an end-to-end twist by a butylene or octylene tether, as indicated by the subscript.

S3 Characterization

S3.1 NMR

***PPP*-HT$_4$H**

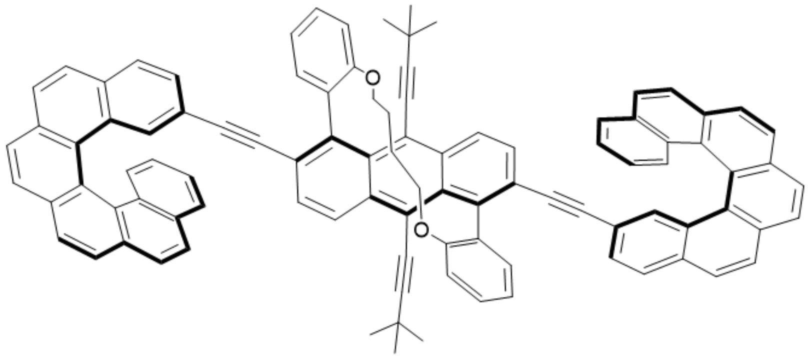

Following the general procedure, 50 mg of *P*-**T$_4$** and 100 mg of *P*-**H** were used to obtain *PPP*-**HT$_4$H** as a dark yellow solid (19 mg, 14% yield).

^{1}H NMR (400 MHz, $CDCl_3$) δ 8.32 (d, *J* = 8.9 Hz, 2H), 8.03 (s, 1H), 8.01 (s, 6H), 7.97 (s, 5H), 7.94 (d, *J* = 5.7 Hz, 2H), 7.93 – 7.82 (m, 8H), 7.72 (d, *J* = 8.2 Hz, 2H), 7.68 – 7.63 (m, 2H), 7.62 – 7.55 (m, 2H), 7.39 – 7.27 (m, 4H), 7.16 – 6.94 (m, 4H), 6.78 (ddd, *J* = 8.4, 6.9, 1.4 Hz, 2H), 6.60 (d, *J* = 8.3 Hz, 2H), 3.59 (d, *J* = 8.2 Hz, 2H), 3.39 – 3.12 (m, 2H), 1.16 (s, 18H), 0.71 (t, *J* = 12.9 Hz, 2H), 0.50 (t, *J* = 13.2 Hz, 2H); **^{13}C NMR** (101 MHz, $CDCl_3$) δ 155.82, 138.44, 133.13, 132.75, 132.17, 132.11, 132.01, 131.52, 131.40, 131.10, 129.66, 129.54, 129.39, 128.94, 128.85, 128.05, 127.82 (d, *J* = 5.6 Hz), 127.62 – 127.29 (m), 127.24, 127.10, 126.90 (d, *J* = 9.0 Hz), 126.20, 125.87 (d, *J* = 4.0 Hz), 124.69, 124.09, 121.95, 120.12, 119.11, 118.30, 113.40, 109.98, 92.40 (d, *J* = 438.5 Hz), 65.82, 30.84, 28.43, 25.94. MALDI-TOF m/z:1276.502 (M) calcd. for $C_{98}H_{68}O_2$: 1276.521. UV-vis (THF): λ_{max} (ε) = 239 nm (109555 M^{-1} cm^{-1}), 492 nm (19548 M^{-1} cm^{-1}).

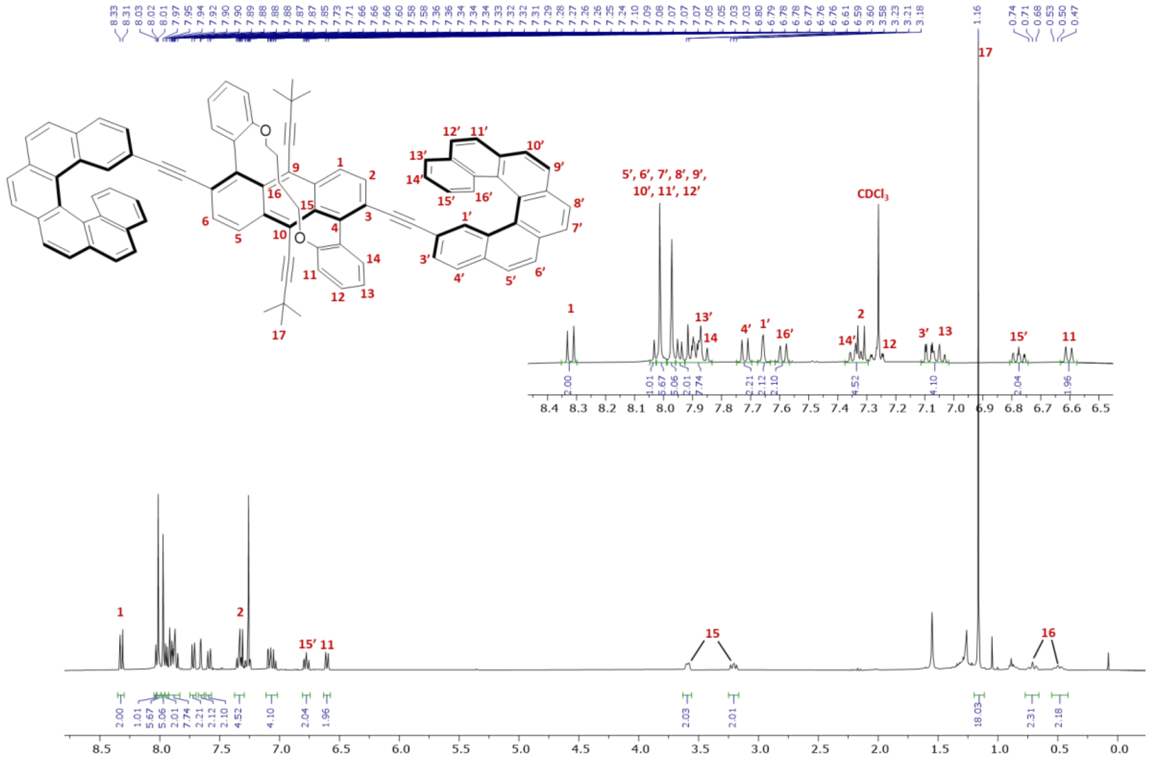

Figure S 1. ^{1}H NMR (400 MHz) of *PPP*-**HT$_4$H** in $CDCl_3$, measured at 298 K.

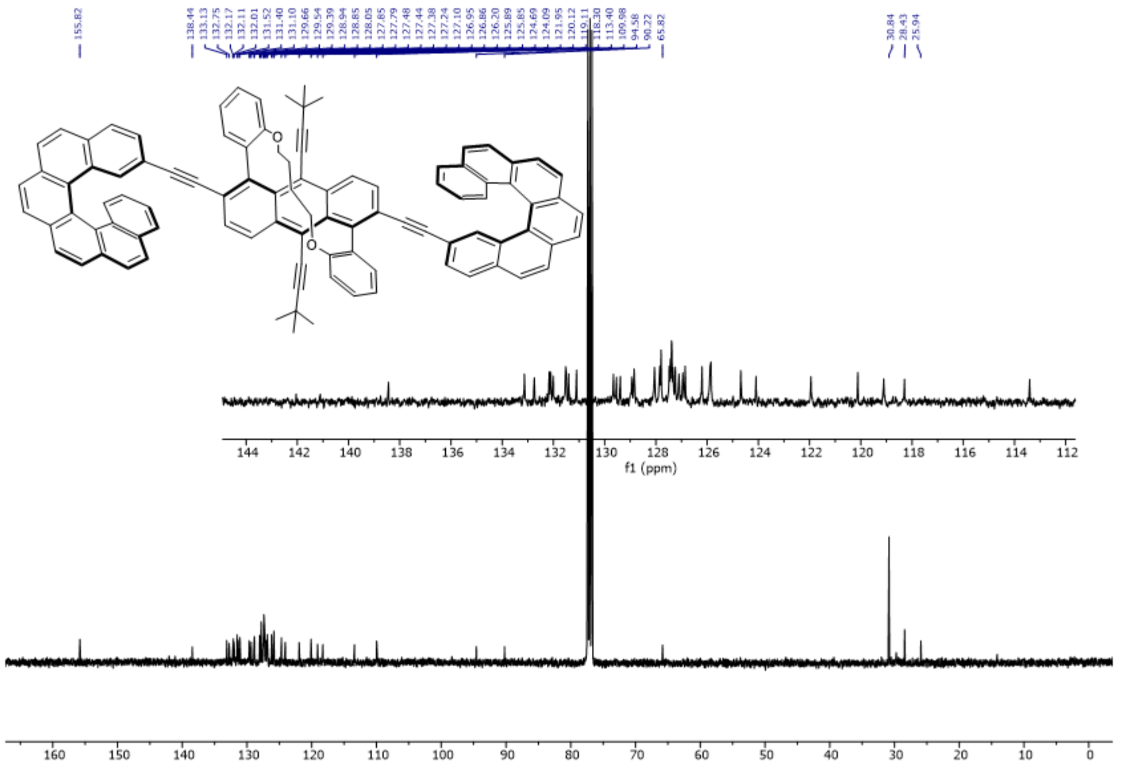

Figure S 2. ^{13}C NMR (101 MHz) of *PPP*-**HT$_4$H** in $CDCl_3$, measured at 298 K.

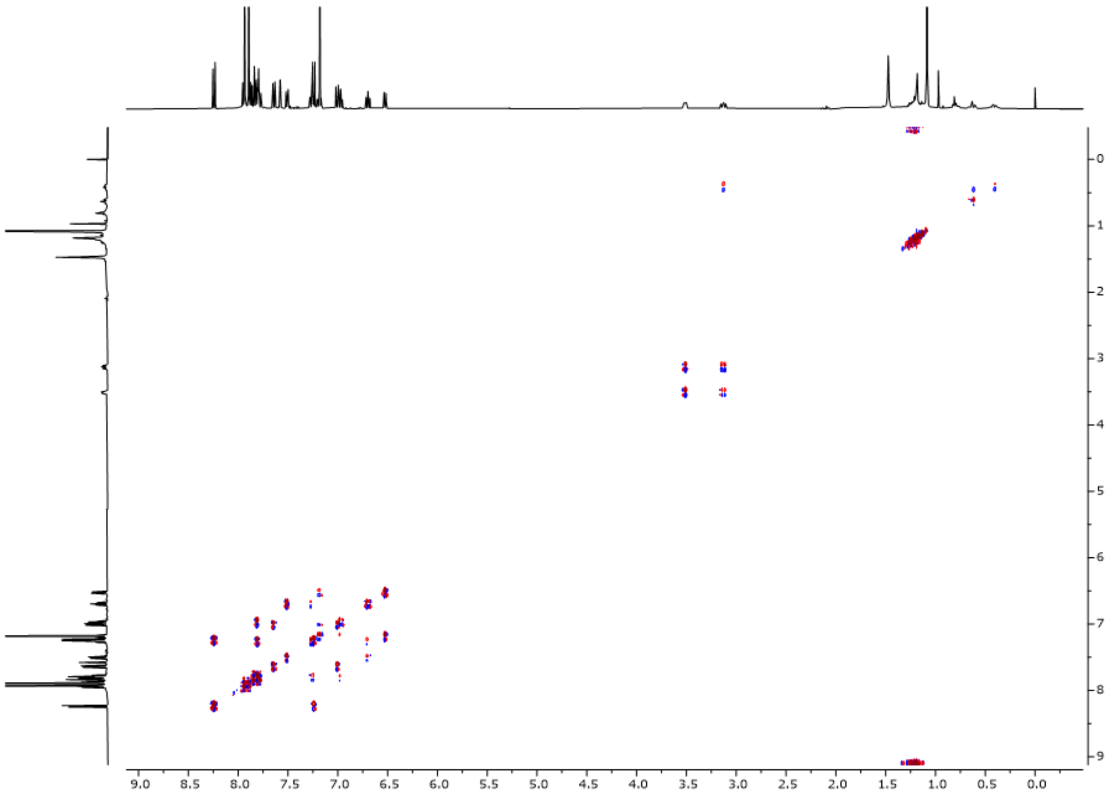

Figure S 3. COSY NMR (400 MHz) of *PPP*-**HT$_4$H** in $CDCl_3$, measured at 298 K.

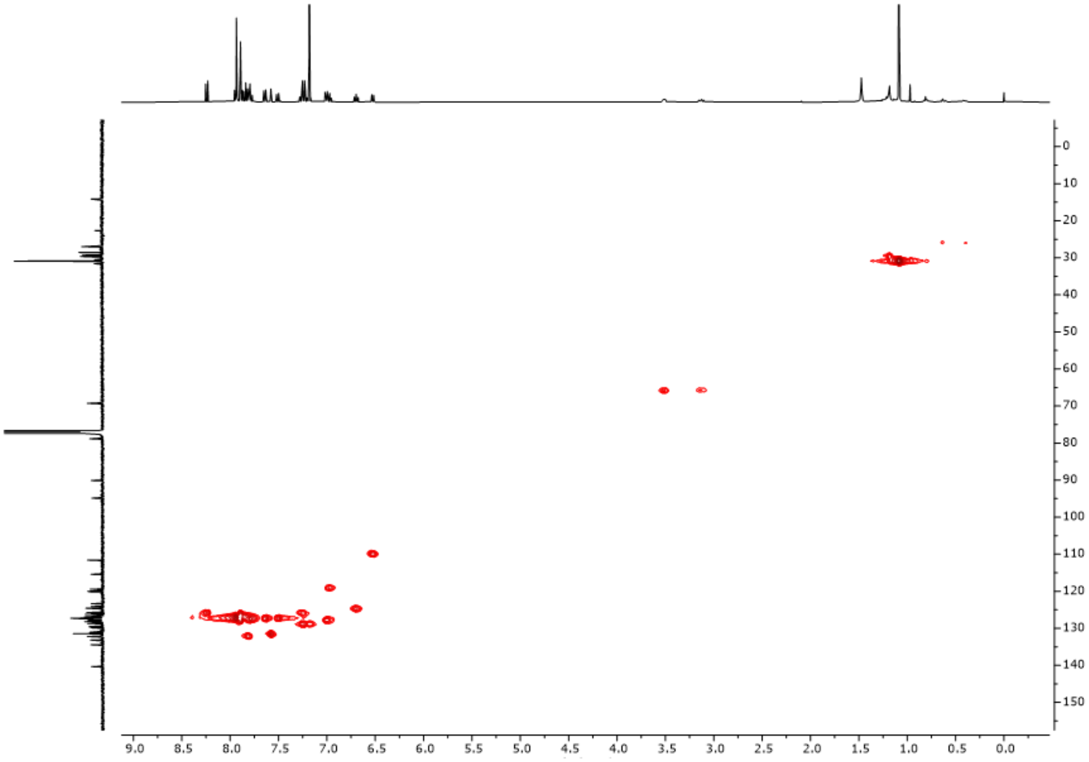

Figure S 4. HSQC NMR (400 MHz) of *PPP*-**HT$_4$H** in $CDCl_3$, measured at 298 K.

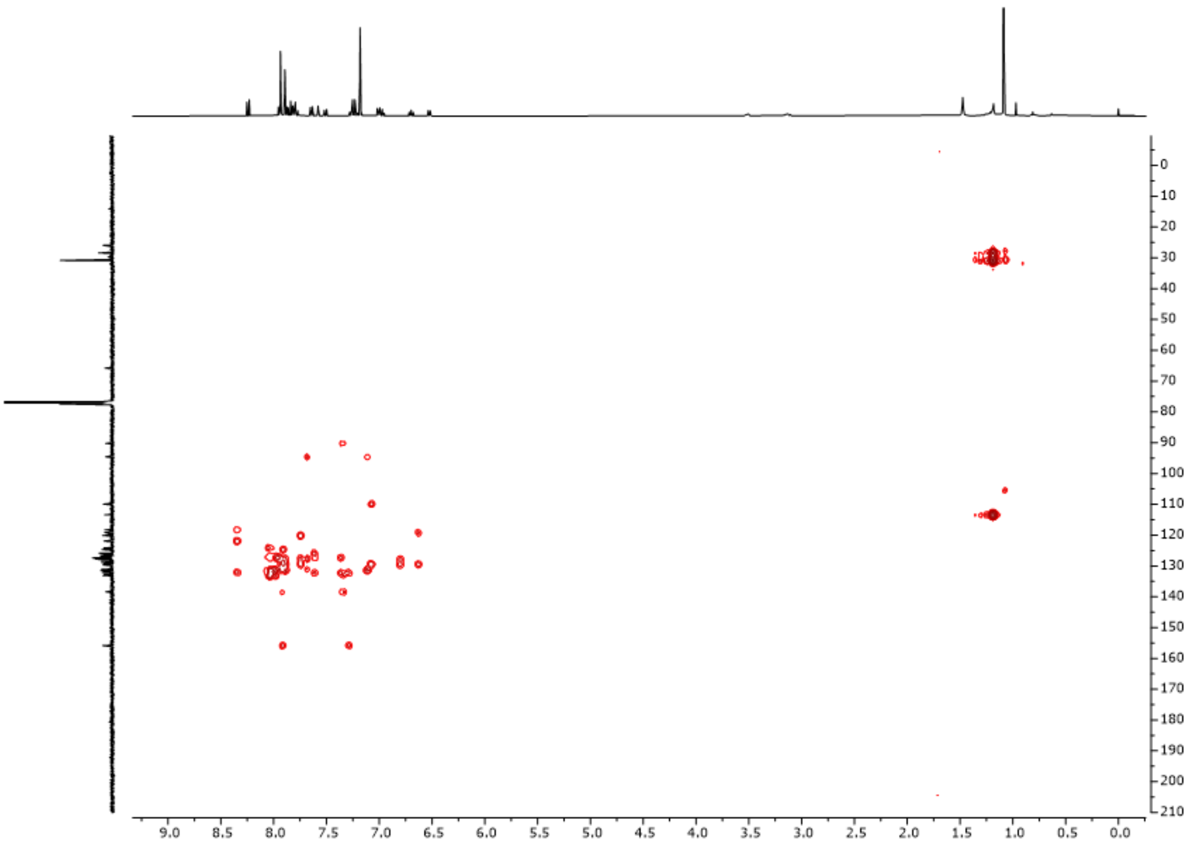

Figure S 5. HSQC NMR (400 MHz) of *PPP*-**HT$_4$H** in $CDCl_3$, measured at 298 K.

MMM-**HT$_4$H**

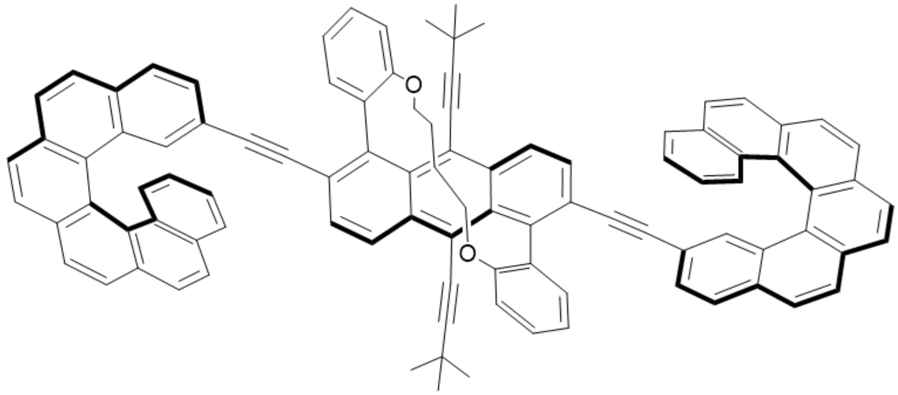

Following the general procedure, 50 mg of *M*-**T$_4$** and 100 mg of *M*-**H** were used to obtain *MMM*-**HT$_4$H** as a dark yellow solid (13 mg, 13% yield). NMR spectra of *MMM*-**HT$_4$H** are consistent the NMR spectra of compound *PPP*-**HT$_4$H**. HR-ESI-MS m/z:1276.5194 (M) calcd. for $C_{98}H_{68}O_2$: 1276.5214. UV-vis (THF): λ_{max} (ε) = 239 nm (109555 M^{-1} cm^{-1}), 492 nm (19548 M^{-1} cm^{-1}).

PP-**T$_4$H**

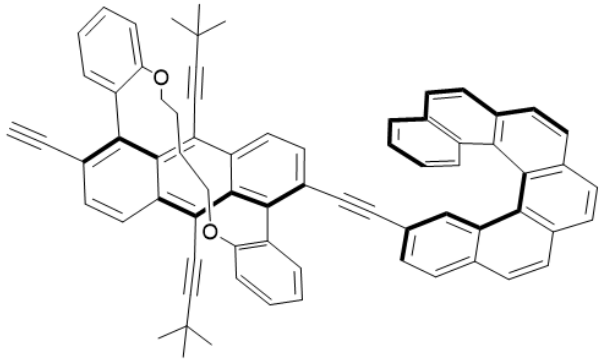

Following the general procedure , 50 mg of *P*-**T$_4$** and 100 mg of *P*-**H** were used to obtain *PP*-**T$_4$H** as a dark yellow solid (20 mg, 26% yield).

^{1}H NMR (400 MHz, $CDCl_3$) δ 8.32 (dd, *J* = 9.0, 5.2 Hz, 2H), 8.01 (s, 3H), 7.97 (q, *J* = 2.5 Hz, 4H), 7.94 (d, *J* = 5.2 Hz, 1H), 7.92 (s, 1H), 7.90 – 7.83 (m, 3H), 7.74 – 7.70 (m, 1H), 7.66 (dd, *J* = 1.5, 0.7 Hz, 1H), 7.61 – 7.56 (m, 1H), 7.37 – 7.28 (m, 4H), 7.12 (td, *J* = 7.5, 1.0 Hz, 1H), 7.08 (dd, *J* = 8.2, 1.5 Hz, 1H), 7.04 (td, *J* = 7.5, 1.1 Hz, 1H), 6.77 (ddd, *J* = 8.4, 6.9, 1.4 Hz, 1H), 6.64 (ddd, *J* = 21.8, 8.3, 1.1 Hz, 1H), 3.62 (d, *J* = 8.1 Hz, 2H), 3.25 (dt, *J* = 20.4, 10.4 Hz, 2H), 3.11 (s, 1H), 1.14 (d, *J* = 15.2 Hz, 18H); **^{13}C NMR** (101 MHz, $CDCl_3$) δ 131.77, 131.54, 131.45 (d, *J* = 9.3 Hz), 131.12, 129.65, 129.47, 129.36 (d, *J* = 6.0 Hz), 129.29, 129.16, 129.04, 128.89, 128.04, 127.84, 127.47, 127.39, 127.29 (d, *J* = 8.1 Hz), 127.04 (d, *J* = 13.0 Hz), 126.86, 126.16 (d, *J* = 7.9 Hz), 124.68, 124.08, 122.13, 120.48, 120.06, 119.35, 119.12, 118.48 (d, *J* = 9.6 Hz), 113.66 (d, *J* = 5.8 Hz), 110.02 (d, *J* = 10.9 Hz), 94.69, 90.12, 84.33, 81.63, 65.86 (d, *J* = 10.5 Hz), 30.81 (d, *J* = 5.2 Hz), 29.72, 28.41 (d, *J* = 2.9 Hz), 25.93 (d, *J* = 3.4 Hz). HR-ESI-MS m/z: 951.4179 (M) calcd. for $C_{72}H_{54}O_2$: 951.4197. UV-vis (THF): λ_{max} (ε) = 314 nm (50215 M^{-1} cm^{-1}), 482 nm (14243 M^{-1} cm^{-1}).

MM-**T$_4$H**

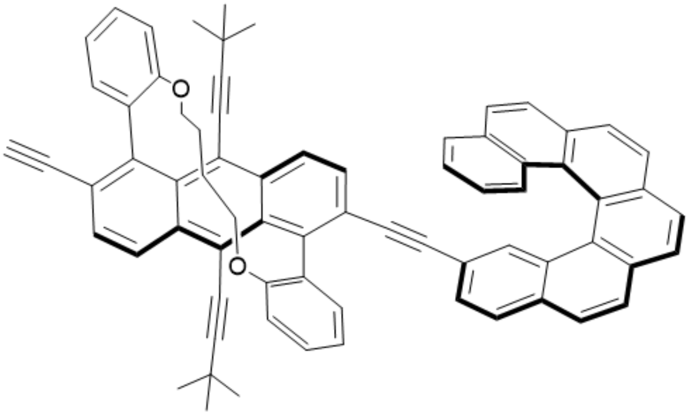

Following the general procedure, 50 mg of *M*-**T$_4$** and 100 mg of *M*-**H** were used to obtain *MM*-**T$_4$H** as a dark yellow solid (10 mg, 13% yield). NMR spectra of *MM*-**T$_4$H** are consistent with the corresponding NMR spectra of *PP*-**T$_4$H**. MALDI-TOF m/z: 950.400 (M) calcd. for $C_{72}H_{54}O_2$: 950.412.

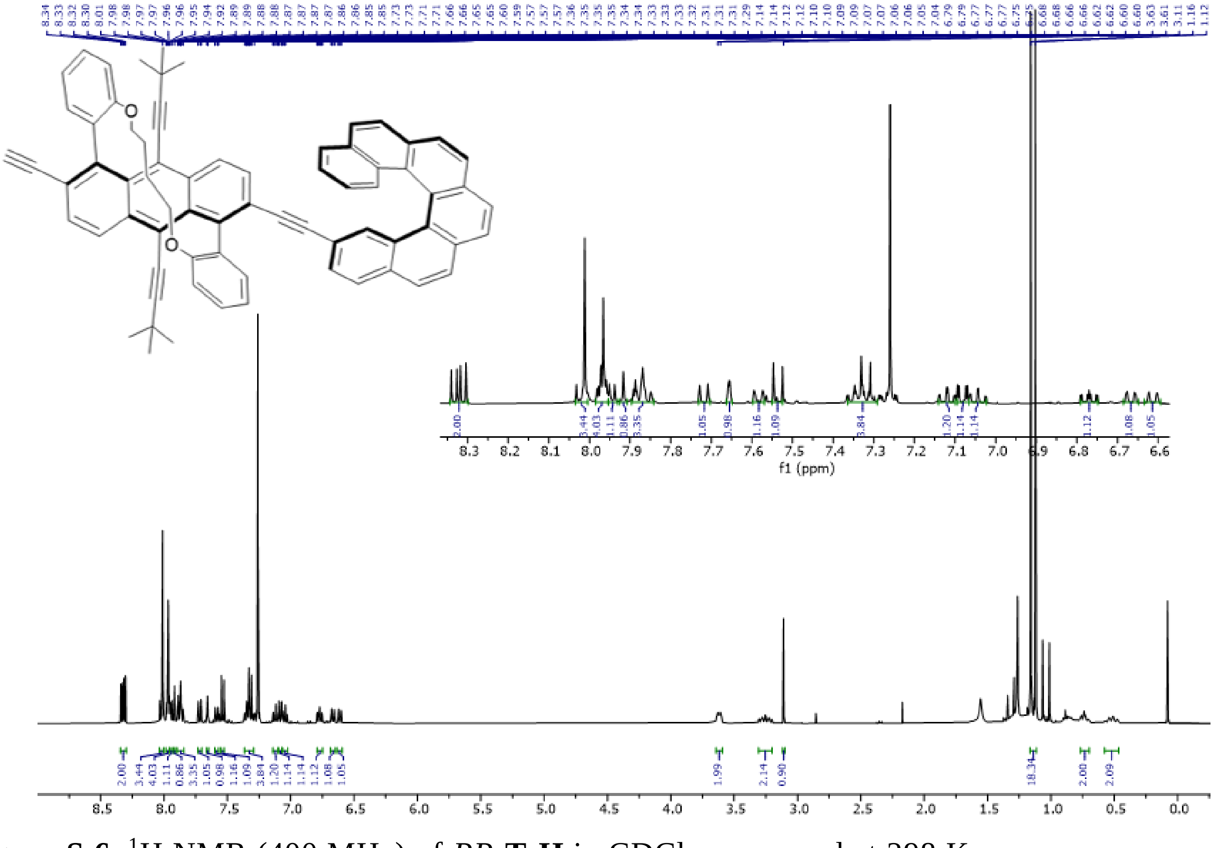

Figure S 6. ^{1}H NMR (400 MHz) of *PP*-**T$_4$H** in $CDCl_3$, measured at 298 K.

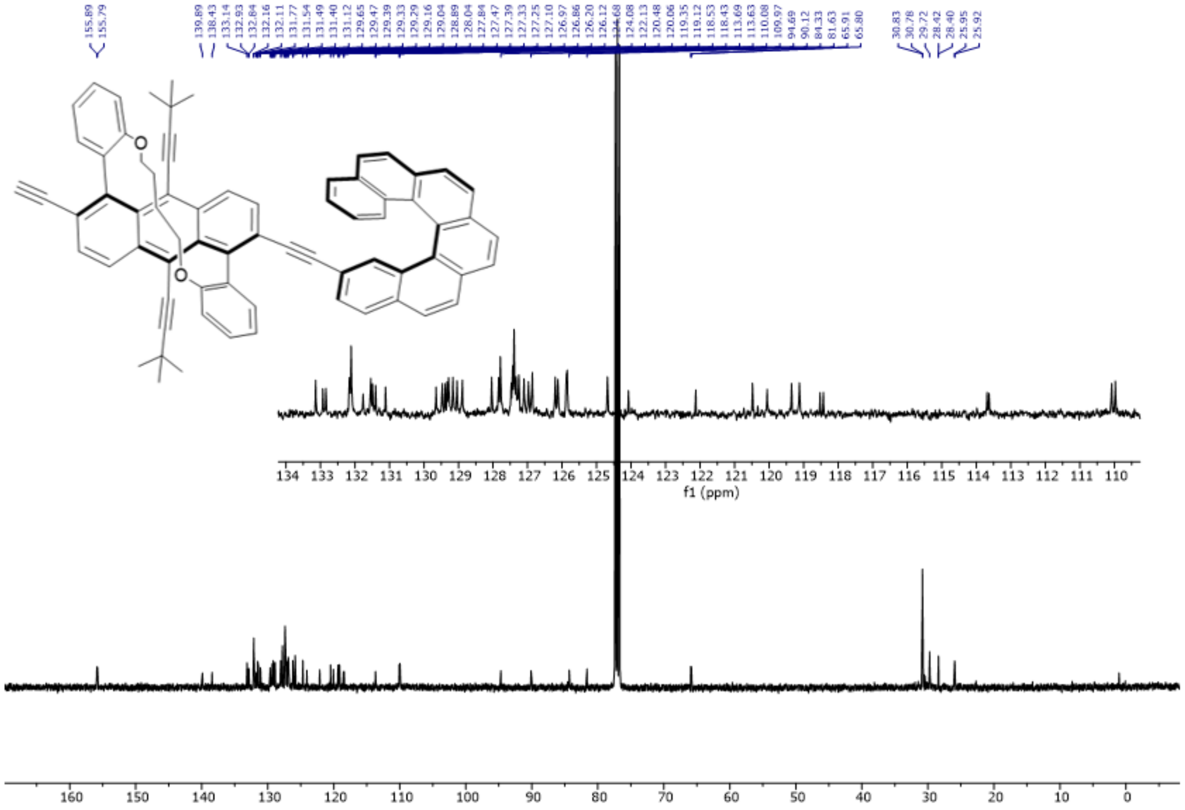

Figure S 7. ^{13}C NMR (101 MHz) of *PP*-**T$_4$H** in $CDCl_3$, measured at 298 K.

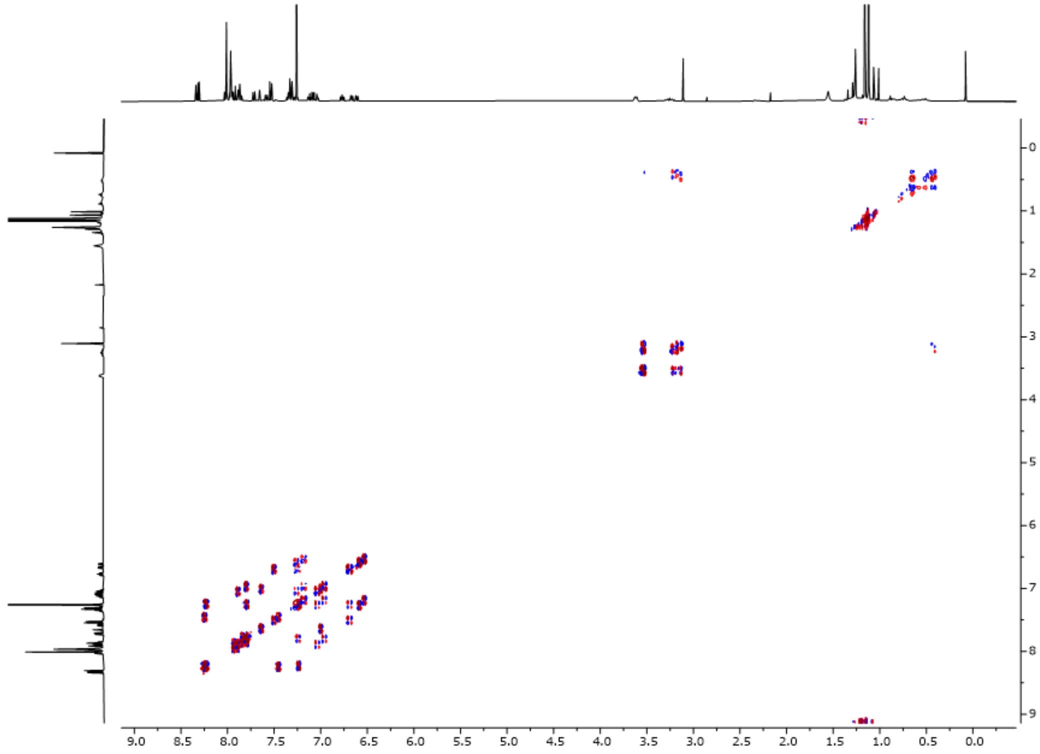

Figure S 8. COSY (400 MHz) of *PP*-**T$_4$H** in $CDCl_3$, measured at 298 K.

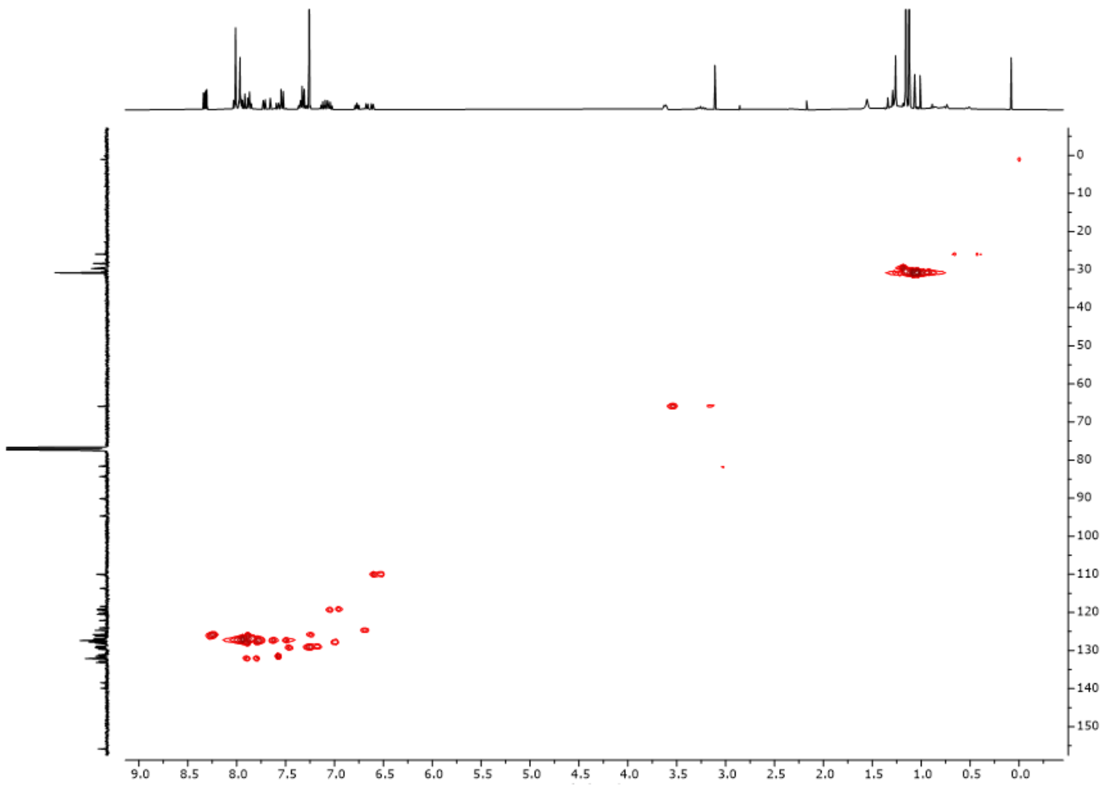

Figure S 9. HSQC (400 MHz) of ***PP*-T$_4$H** in $CDCl_3$, measured at 298 K.

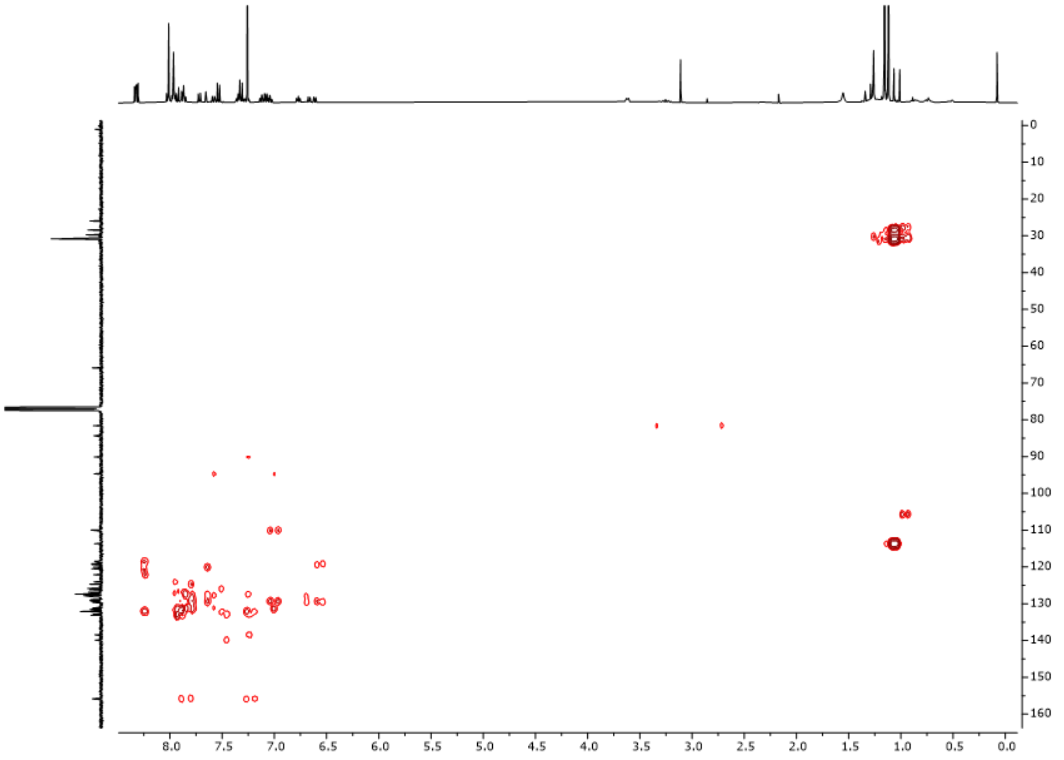

Figure S 10. HMBC (400 MHz) of ***PP*-T$_4$H** in $CDCl_3$, measured at 298 K.

MPM-$\mathbf{HT_4H}$

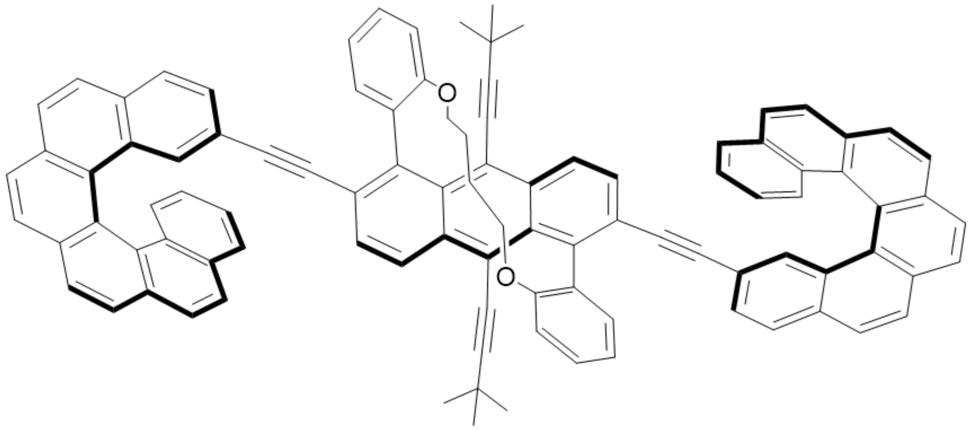

Following the general procedure, 50 mg of *P*-$\mathbf{T_4}$ and 100 mg of *M*-**H** were used to obtain *MPM*-$\mathbf{HT_4H}$ as a dark yellow solid (17 mg, 17% yield).

^{1}H NMR (500 MHz, $CDCl_3$) δ 8.32 (dd, *J* = 8.9, 0.8 Hz, 2H), 8.13 – 8.02 (m, 10H), 7.99 (d, *J* = 8.1 Hz, 2H), 7.94 (d, *J* = 8.4 Hz, 2H), 7.90 (d, *J* = 8.0 Hz, 2H), 7.86 (d, *J* = 8.5 Hz, 2H), 7.81 (s, 2H), 7.71 (d, *J* = 8.1 Hz, 2H), 7.60 (d, *J* = 8.6 Hz, 2H), 7.56 (dt, *J* = 7.3, 1.4 Hz, 2H), 7.41 (dd, *J* = 8.9, 0.9 Hz, 2H), 7.28 – 7.26 (m, 1H), 7.26 – 7.23 (m, 1H), 7.16 – 7.09 (m, 2H), 7.04 (td, *J* = 7.9, 1.5 Hz, 2H), 6.72 (ddd, *J* = 8.0, 6.7, 1.2 Hz, 2H), 6.55 (t, *J* = 7.5 Hz, 2H), 6.49 (d, *J* = 8.1 Hz, 2H), 3.54 (d, *J* = 8.1 Hz, 2H), 3.15 (dd, *J* = 12.0, 8.4 Hz, 2H), 1.29 (m, 8H), 1.21 (s, 18H), 0.76 – 0.61 (m, 2H), 0.48 (t, *J* = 13.3 Hz, 2H). **^{13}C NMR** (126 MHz, $CDCl_3$) δ 155.73, 138.60, 133.40, 132.86, 132.32, 132.19, 132.02, 131.73, 131.32, 129.83, 129.72, 129.48, 129.13, 128.90, 128.40, 128.12, 128.03, 127.70, 127.62, 127.56, 127.49, 127.43, 127.31, 127.18, 127.12, 126.44, 126.11, 125.98, 124.86, 124.32, 121.98, 120.27, 119.36, 118.45, 113.55, 109.82, 94.33, 90.30, 65.86, 31.74, 31.01, 28.59, 26.03. MALDI-TOF m/z:1276.511 (M) calcd. for $C_{98}H_{68}O_2$: 1276.521. UV-vis (THF): λ_{max} (ε) = 239 nm (111348 M^{-1} cm^{-1}), 494 nm (22739 M^{-1} cm^{-1}). MALDI-TOF m/z:1276.511 (M) calcd. for $C_{98}H_{68}O_2$:1276.521. UV-vis (THF): λ max (ε) = 239 nm (111348 M -1 cm -1), 494 nm (22739 M -1 cm -1).

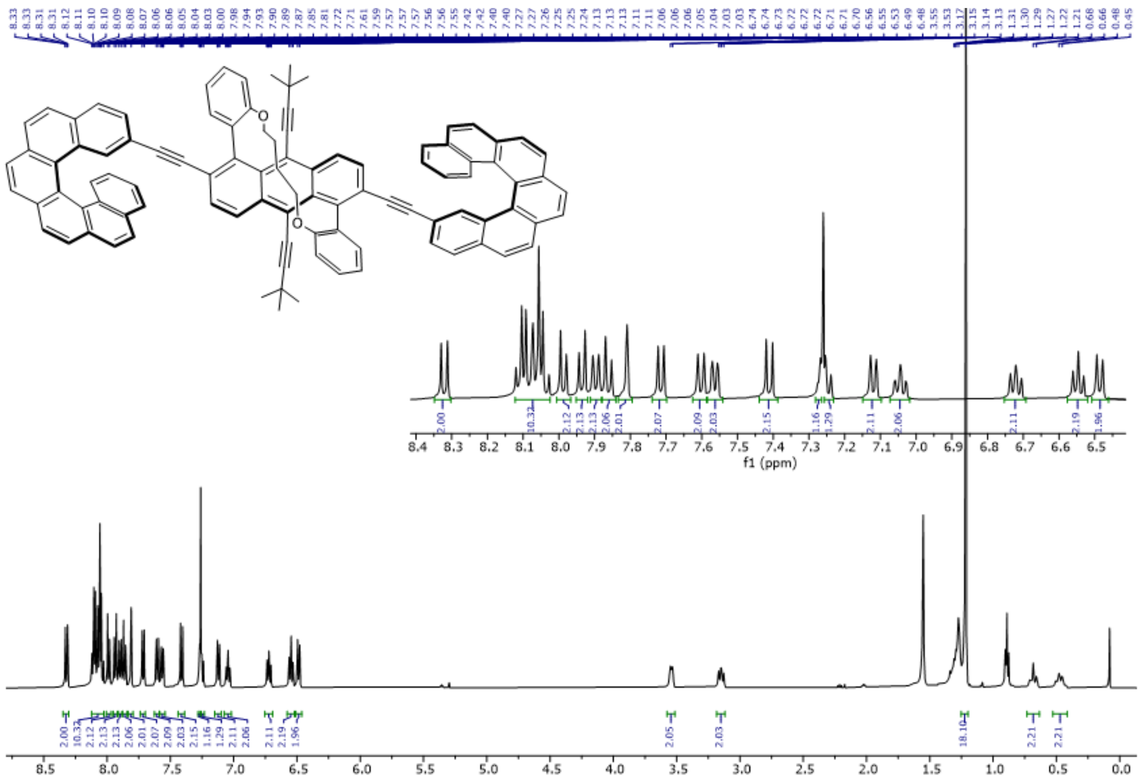

Figure S 11. ^{1}H NMR (500 MHz) of *MPM*-**HT$_4$H** in $CDCl_3$, measured at 298 K**.**

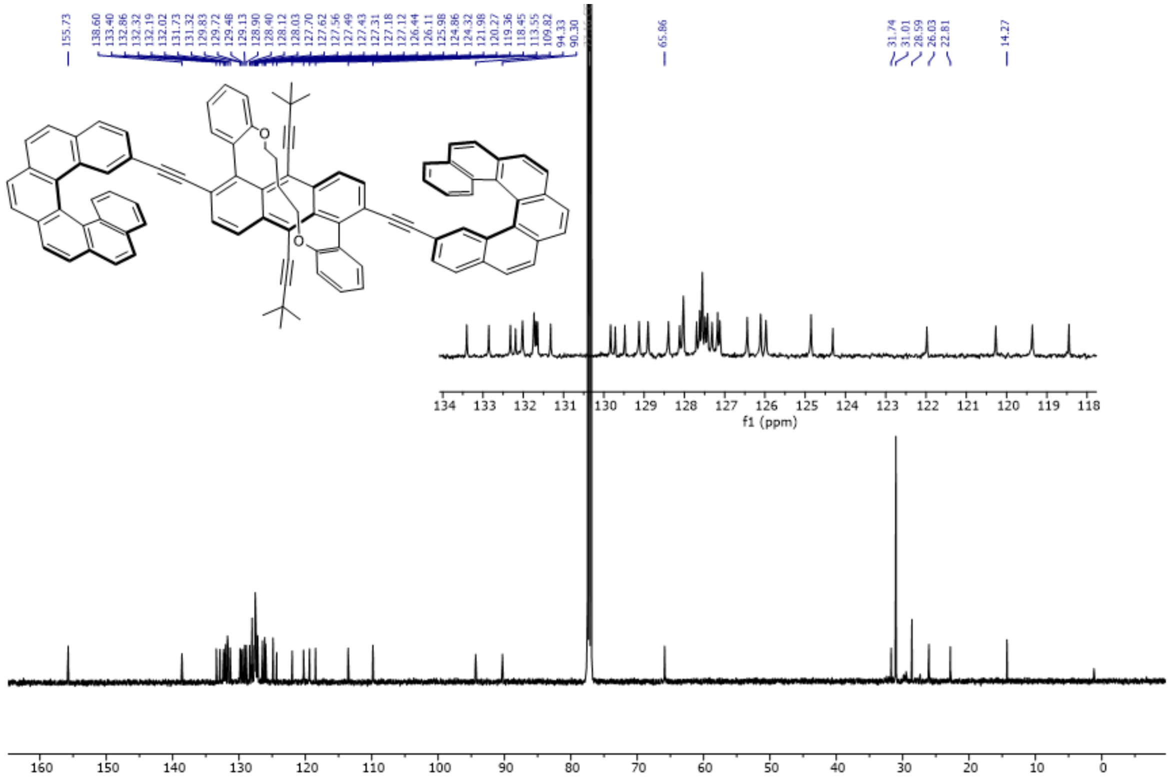

Figure S 12. ^{13}C NMR (125 MHz) of *MPM*-**HT$_4$H** in $CDCl_3$, measured at 298 K.

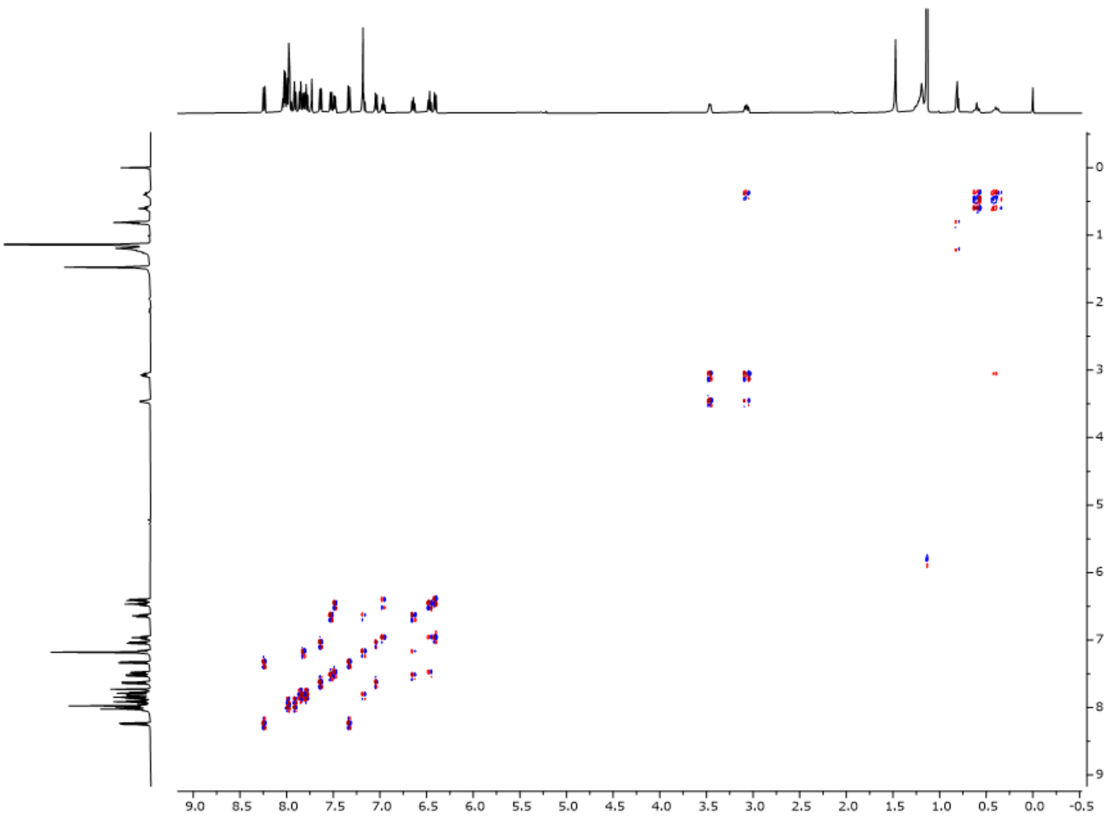

Figure S 13. COSY (500 MHz) of *MPM*-**HT$_4$H** in $CDCl_3$, measured at 298 K.

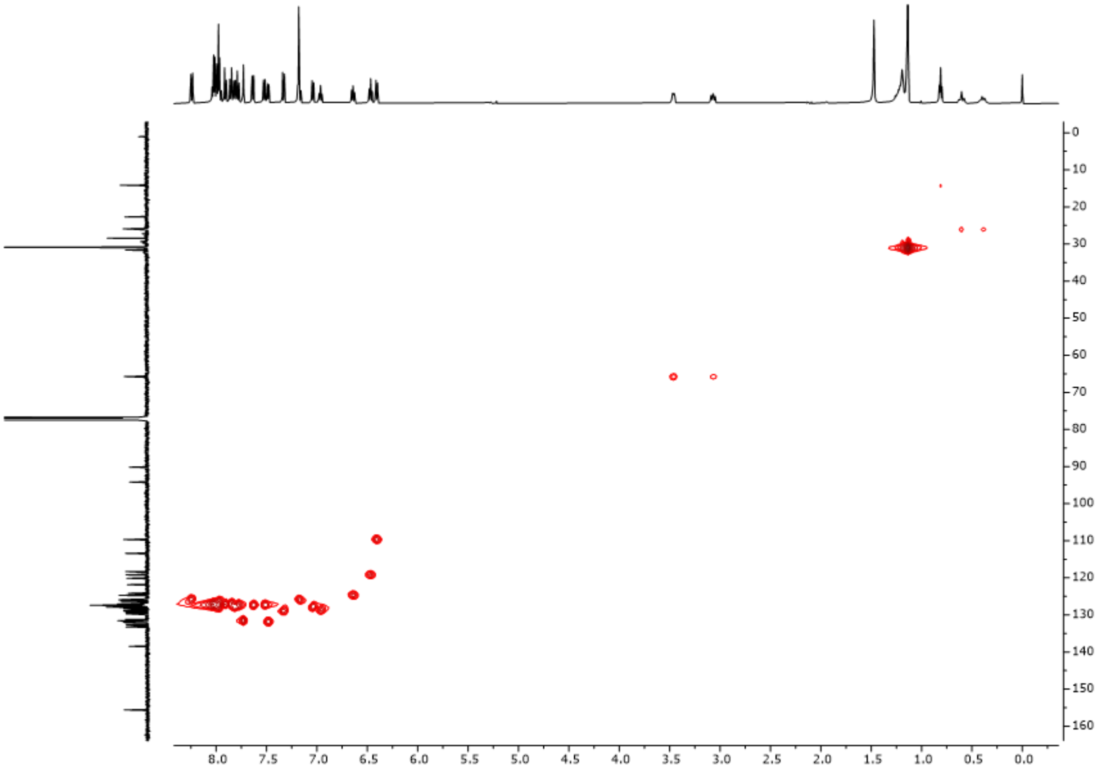

Figure S 14. HSQC (500 MHz) of *MPM*-**HT$_4$H** in $CDCl_3$, measured at 298 K.

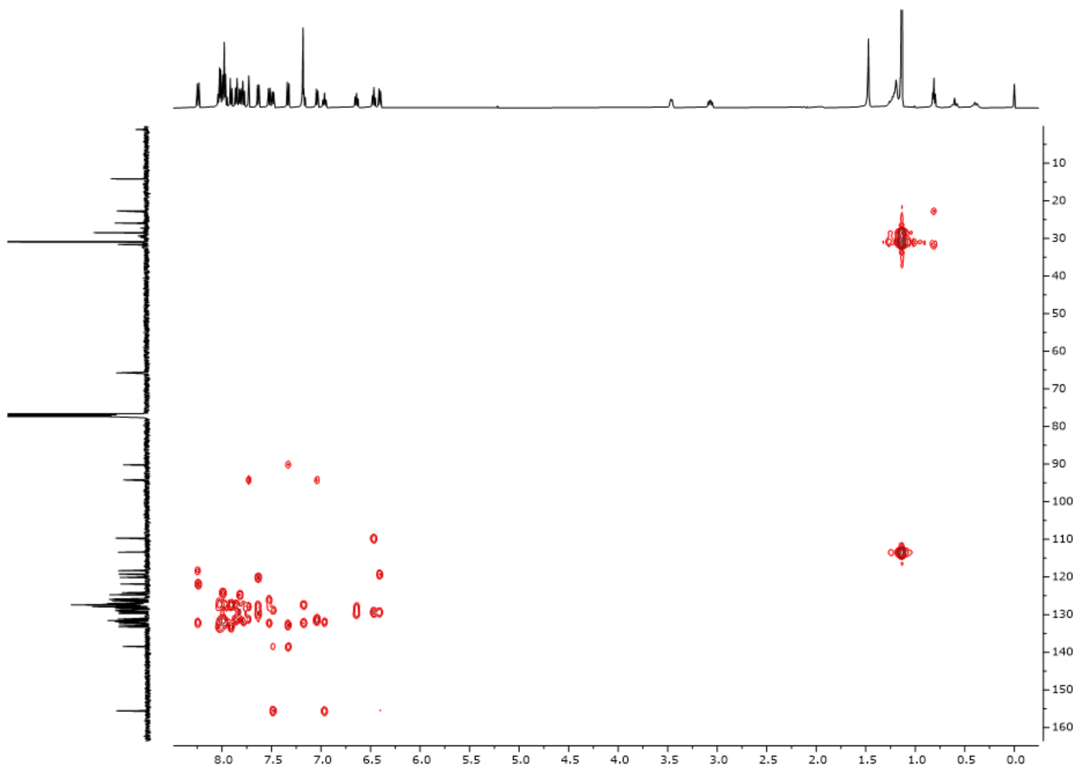

Figure S 15. HMBC (500 MHz) of *MPM*-**HT$_4$H** in $CDCl_3$, measured at 298 K.

PM-T$_4$H

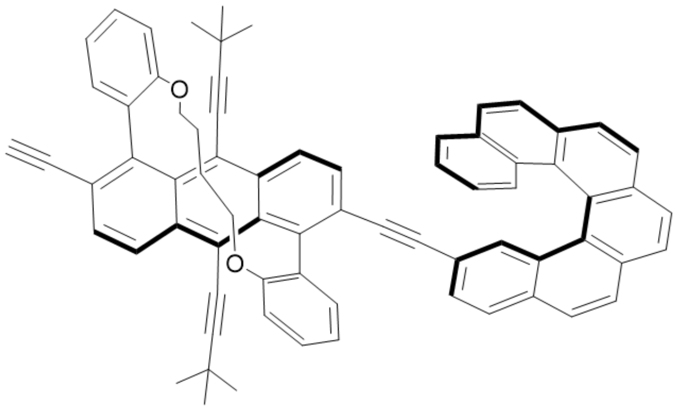

Following the general procedure, 50 mg of *P*-**T$_4$** and 100 mg of *M*-**H** were used to obtain *PM*- **T$_4$H** as a dark yellow solid (10 mg, 13% yield).

^{1}H NMR (500 MHz, $CDCl_3$) δ 8.36 – 8.28 (m, 2H), 8.13 – 8.08 (m, 2H), 8.07 (m, 1H), 8.05 (d, *J* = 2.9 Hz, 1H), 8.03 (m, 1H), 7.99 (d, *J* = 8.1 Hz, 1H), 7.96 (dd, *J* = 7.5, 1.8 Hz, 1H), 7.93 (s, 1H), 7.90 (d, *J* = 7.8 Hz, 1H), 7.86 (d, *J* = 8.5 Hz, 1H), 7.83 – 7.79 (m, 1H), 7.71 (d, *J* = 8.3 Hz, 1H), 7.60 (d, *J* = 8.5 Hz, 1H), 7.57 – 7.50 (m, 1H), 7.41 (dd, *J* = 8.9, 0.6 Hz, 1H), 7.35 (td, *J* = 7.8, 1.8

Hz, 2H), 7.16 – 7.09 (m, 2H), 7.07 – 7.00 (m, 1H), 6.72 (ddd, *J* = 8.5, 6.7, 1.3 Hz, 1H), 6.67 (d, *J* = 8.1 Hz, 1H), 6.53 (td, *J* = 7.5, 1.0 Hz, 1H), 6.49 (d, *J* = 8.2 Hz, 1H), 3.64 (m, 1H), 3.56 (d, *J* = 8.3 Hz ,1H), 3.33 – 3.25 (m, 1H), 3.21 – 3.14 (m, 1H), 1.16 (d, *J* = 16.8 Hz, 18H), 0.73 (m, 2H), 0.51 (m, 2H). **^{13}C NMR** (126 MHz, $CDCl_3$) δ 155.86 (d, *J* = 40.0 Hz), 140.02, 133.41, 133.05, 132.33, 132.25, 132.02, 131.78, 131.65, 129.84, 129.70, 129.43, 129.30, 129.23, 128.94, 128.38, 128.12, 128.00, 127.62, 127.56, 127.50, 127.44, 127.33, 127.19, 126.45, 126.25, 126.11, 125.98, 124.86, 124.32, 122.15, 120.63, 120.21, 119.50, 119.38, 118.71, 118.54, 113.81 (d, *J* = 12.2 Hz), 110.20, 109.82, 94.43, 90.20, 84.46, 81.76, 66.06, 31.74, 30.96, 29.85, 28.56 (d, *J* = 2.5 Hz), 26.07, 22.81. MALDI-TOF m/z: 950.398 (M) calcd. for $C_{72}H_{54}O_2$: 950.412. UV-vis (THF): λ_{max} (ε) = 314 nm (52397 M^{-1} cm^{-1}), 494 nm (14598 M^{-1} cm^{-1}).

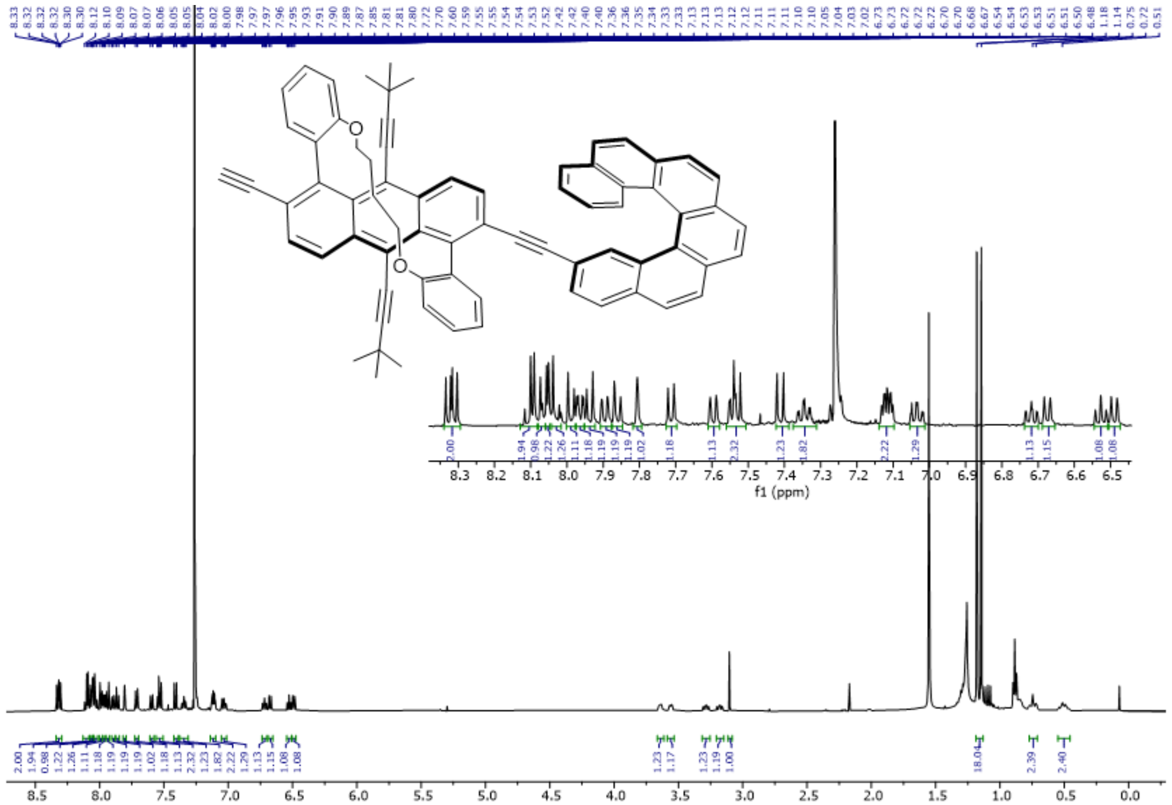

Figure S 16. ^{1}H NMR (500 MHz) of *PM*-**T$_4$H** in $CDCl_3$, measured at 298 K.

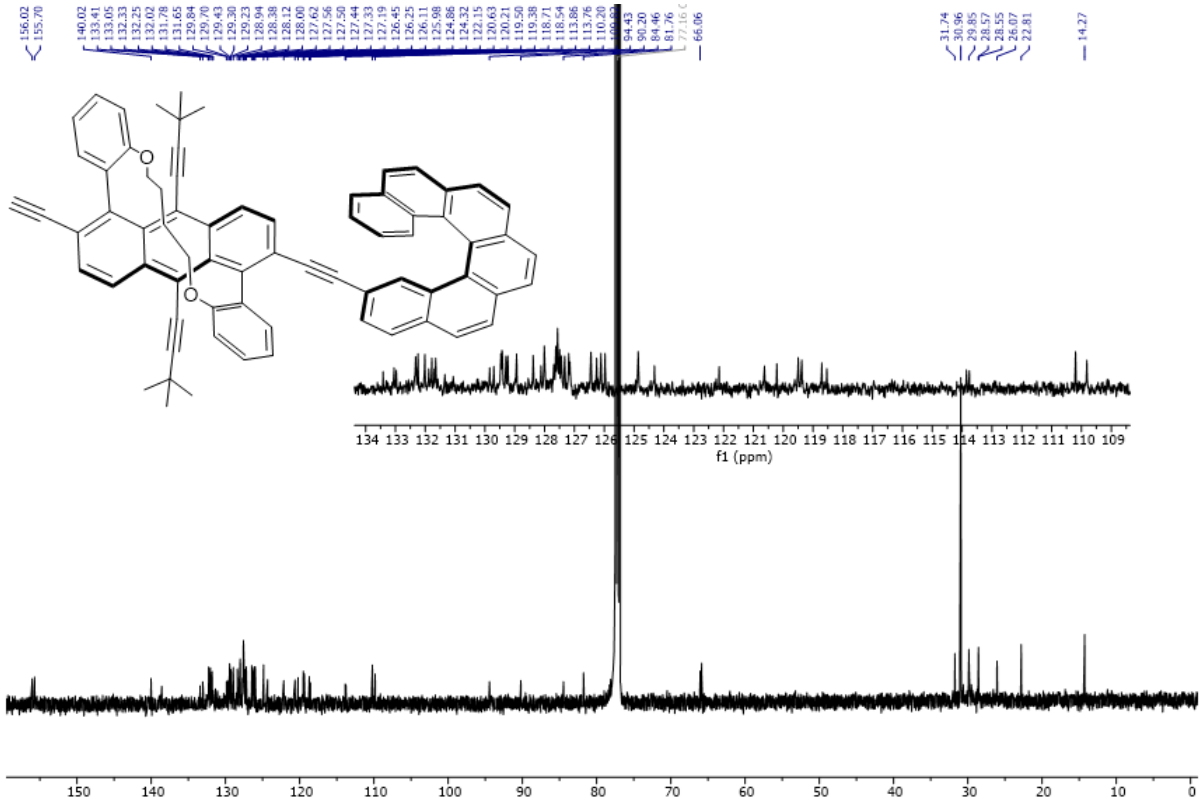

Figure S 17. ^{13}C NMR (126 MHz) of *PM*-**T$_4$H** in $CDCl_3$, measured at 298 K.

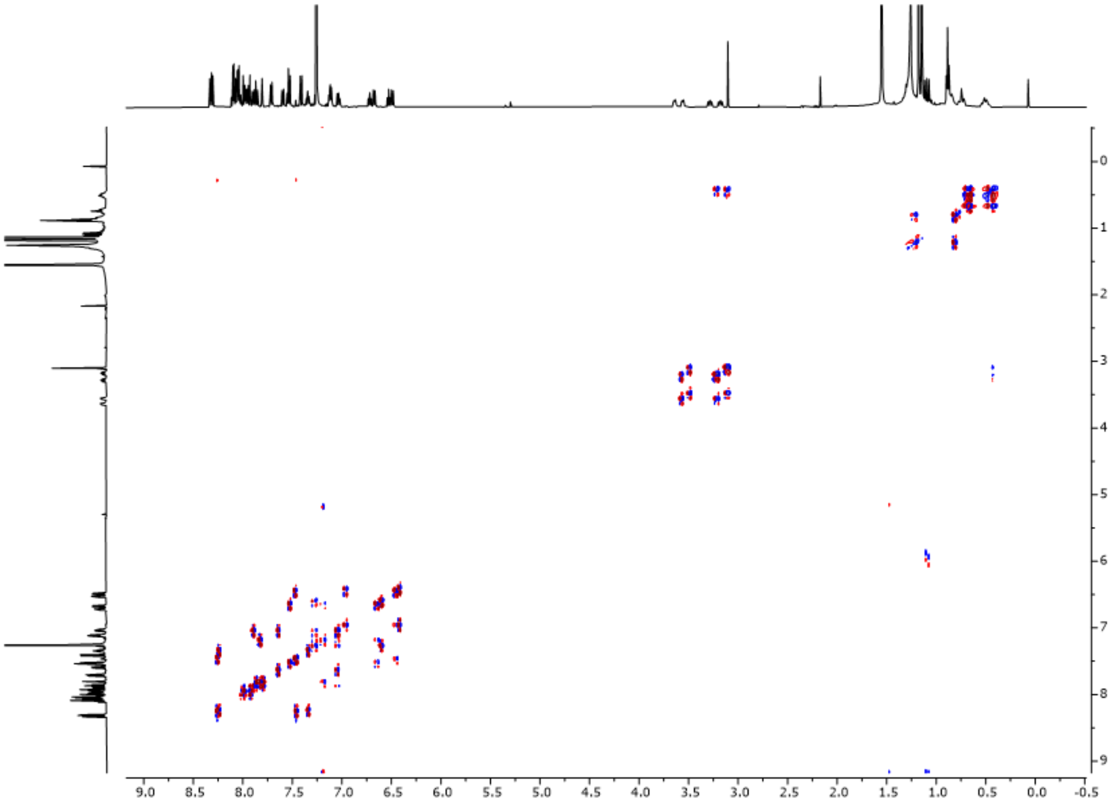

Figure S 18. COSY (500 MHz) of *PM*-**T$_4$H** in $CDCl_3$, measured at 298 K.

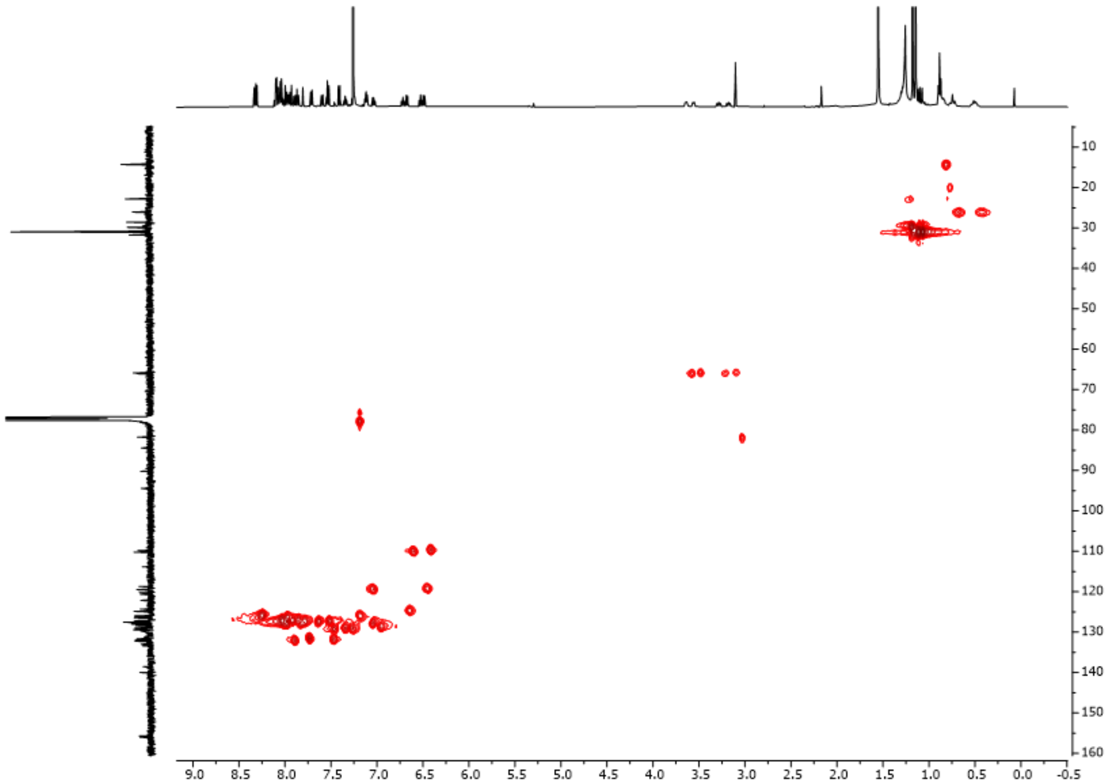

Figure S 19. HSQC (500 MHz) of *PM*-**T$_4$H** in $CDCl_3$, measured at 298 K.

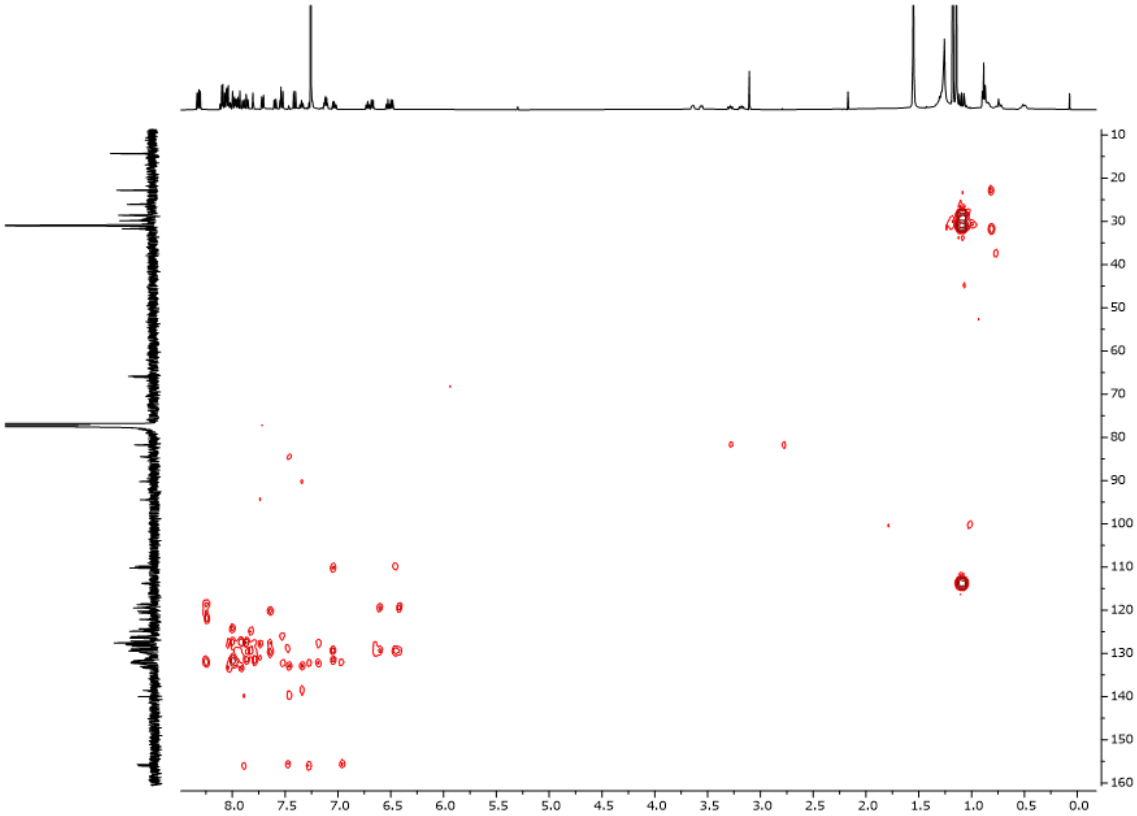

Figure S 20. HMBC (500 MHz) of *PM*-**T$_4$H** in $CDCl_3$, measured at 298 K.

PPP-**HT_8H**

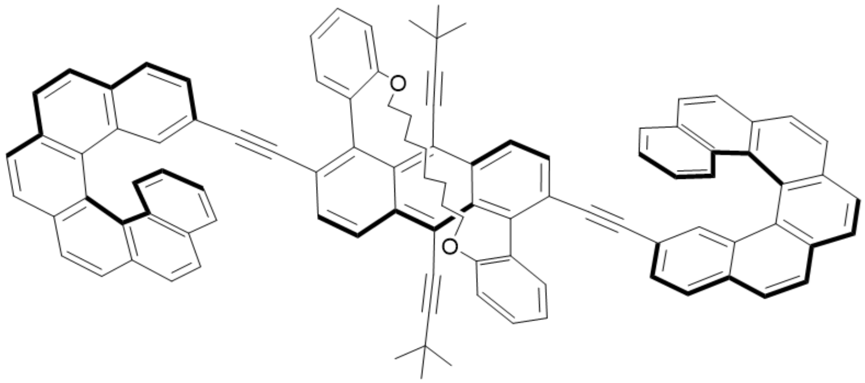

Following the general procedure, 55 mg of *P*-**T_8** and 100 mg of *P*-**H** were used to obtain *PPP*- **H T_8H** as a dark yellow solid (18 mg, 17% yield).

^{1}H NMR (400 MHz, $CDCl_3$) δ 8.64 (dd, *J* = 9.1, 1.6 Hz, 2H), 8.04 – 7.99 (m, 7H), 7.99 – 7.93 (m, 6H), 7.92 (s, 1H), 7.90 (s, 1H), 7.85 (d, *J* = 0.8 Hz, 1H), 7.84 – 7.81 (m, 3H), 7.67 (d, *J* = 8.2 Hz, 2H), 7.59 – 7.50 (m, 4H), 7.39 – 7.31 (m, 4H), 7.31 – 7.27 (m, 2H), 7.25 (d, *J* = 1.2 Hz, 1H), 6.97 (ddd, *J* = 7.4, 6.7, 1.1 Hz, 2H), 6.82 (ddd, *J* = 9.2, 8.0, 1.3 Hz, 4H), 6.71 (ddd, *J* = 8.4, 6.9, 1.3 Hz, 2H), 3.74 (ddq, *J* = 14.0, 8.5, 4.7 Hz, 4H), 1.55 (m, 4H), 1.17 (s, 18H), 0.51 (d, *J* = 40.6 Hz, 8H).

^{13}C NMR (101 MHz, $CDCl_3$) δ 157.83, 140.41, 134.45, 133.14, 132.37, 132.10, 131.61, 131.01, 129.83, 129.58, 129.49, 128.72, 128.50, 128.35, 128.06, 127.82, 127.72, 127.55, 127.40 (d, *J* = 3.5 Hz), 127.28, 127.25, 127.21, 127.08, 126.84, 126.16, 125.86, 124.65, 124.09, 123.43, 115.44, 111.88, 95.05, 90.22, 78.89, 69.27, 30.92, 29.72, 29.50, 29.18, 28.58, 26.85. HR-ESI-MS m/z: 1333.5902 (M) calcd. for $C_{102}H_{76}O_2$: 1333.5918. UV-vis (THF): λ_{max} (ε) = 236 nm (93867 M^{-1} cm^{-1}), 484 nm (18780 M^{-1} cm^{-1}).

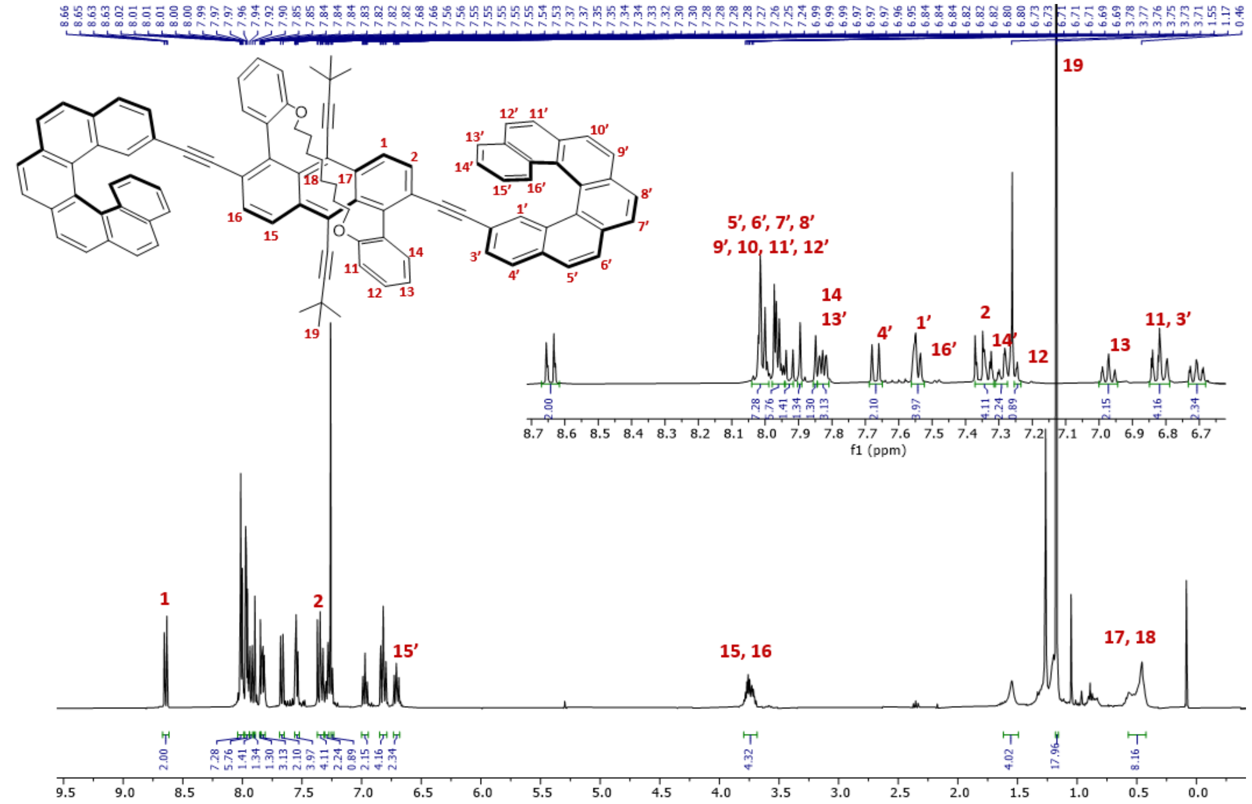

Figure S 21.^{1}H NMR (400 MHz) of *PPP*-**HT$_8$H** in $CDCl_3$, measured at 298 K.

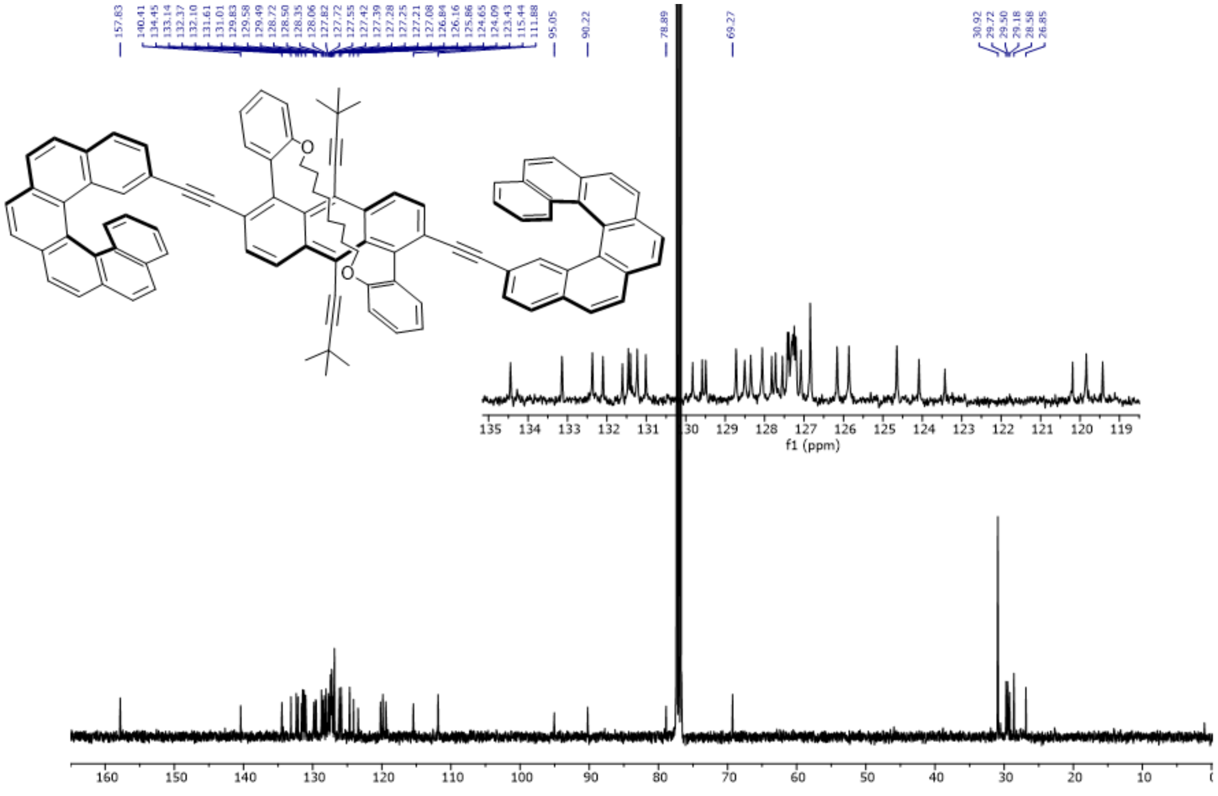

Figure S 22. ^{13}C NMR (101 MHz) of *PPP*-**HT$_8$H** in $CDCl_3$, measured at 298 K.

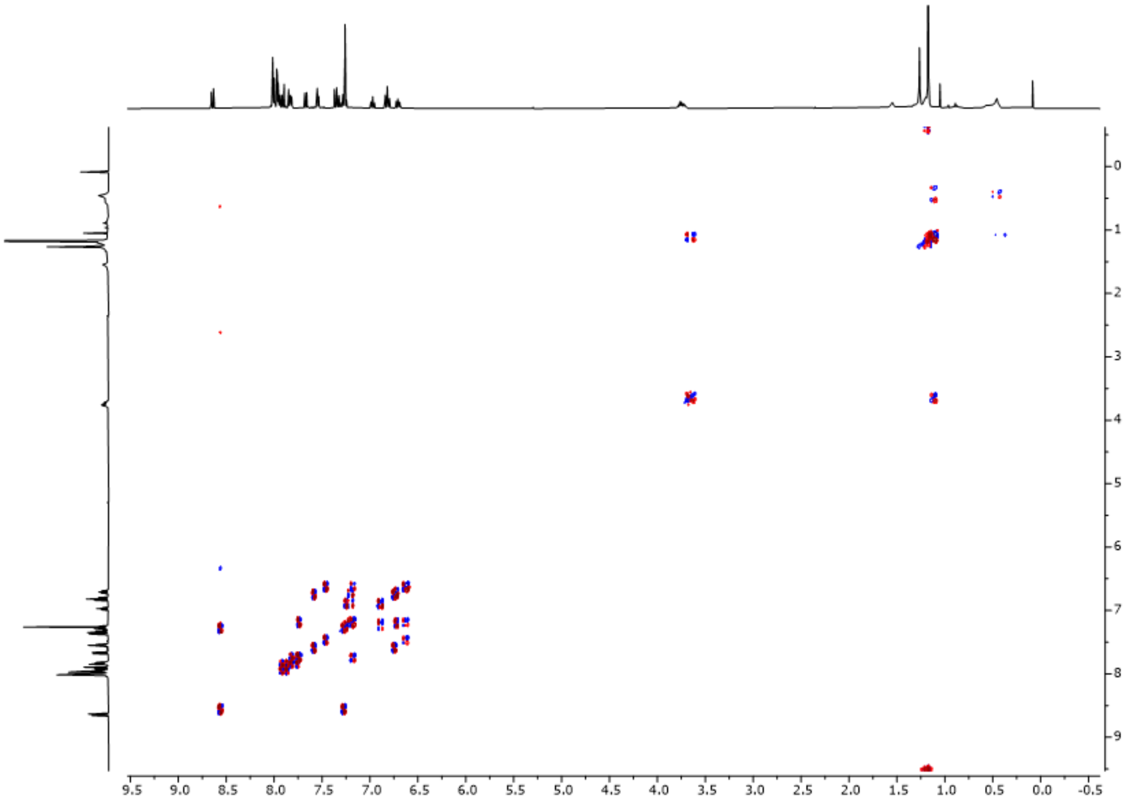

Figure S 23. COSY (400 MHz) of *PPP*-**HT$_8$H** in $CDCl_3$, measured at 298 K.

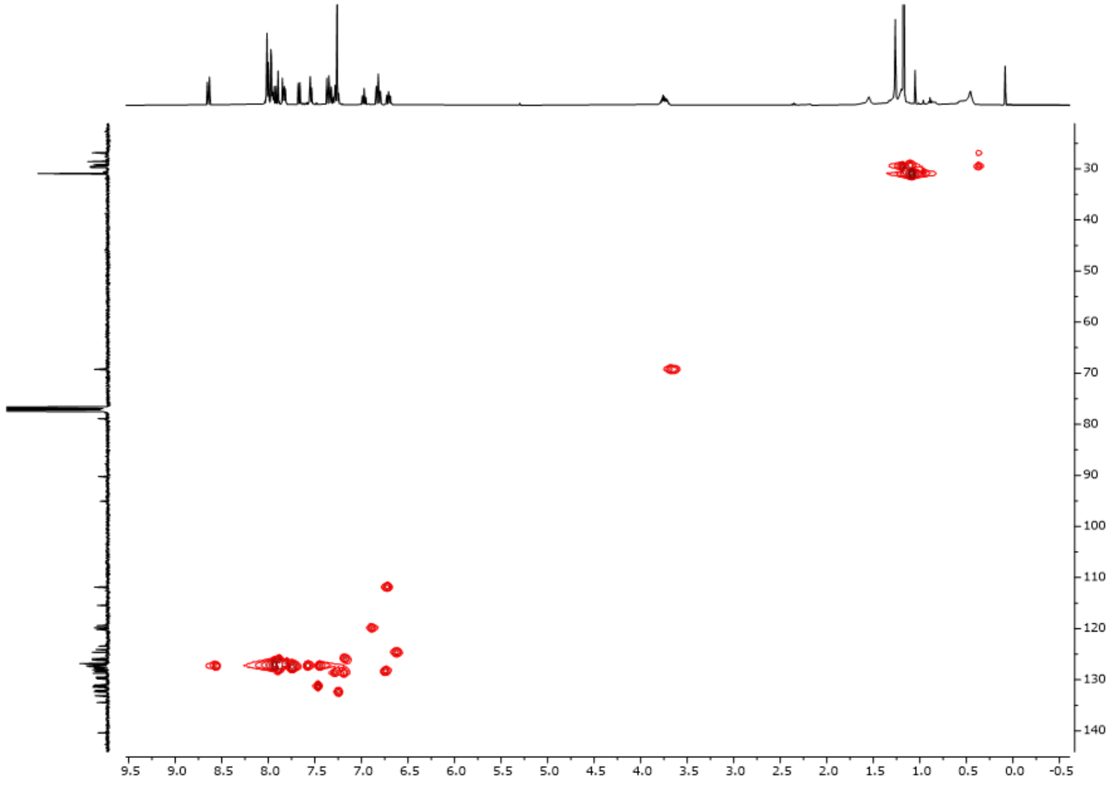

Figure S 24. HSQC (400 MHz) of *PPP*-**HT$_8$H** in $CDCl_3$, measured at 298 K.

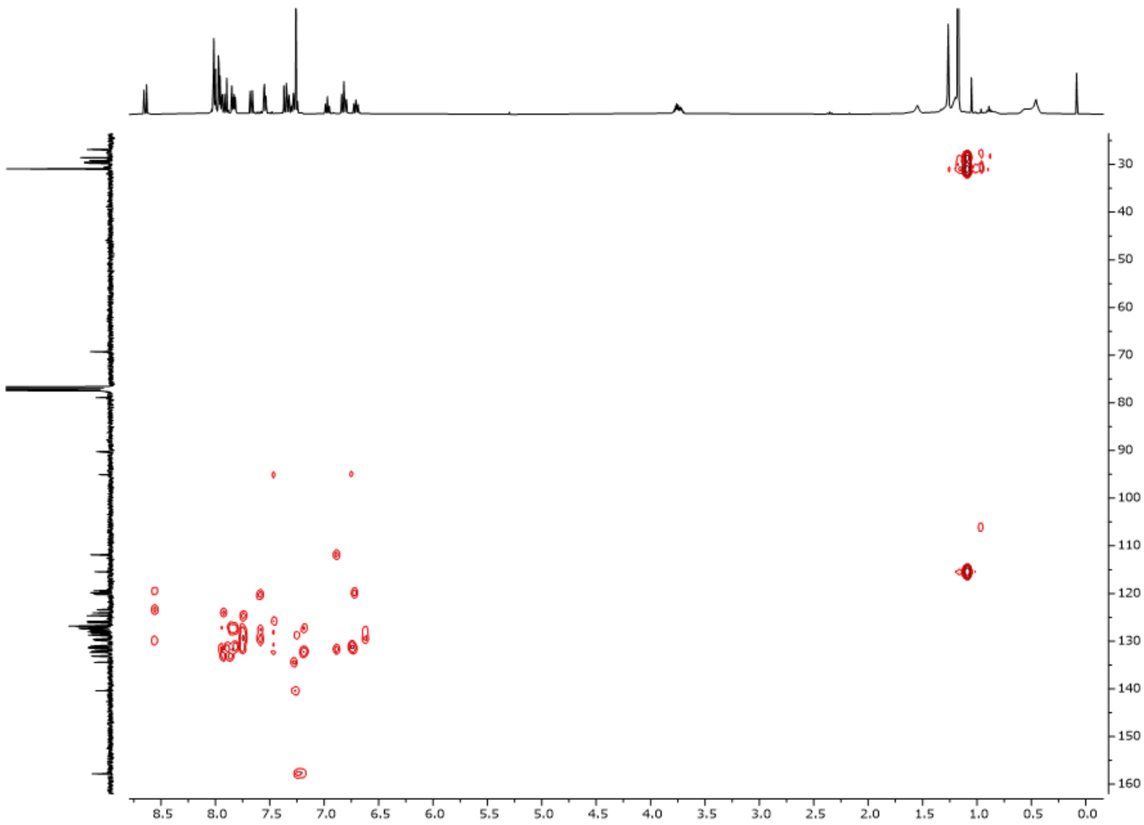

Figure S 25. HMBC (400 MHz) of *PPP*-**HT$_8$H** in $CDCl_3$, measured at 298 K.

MMM-**HT$_8$H**

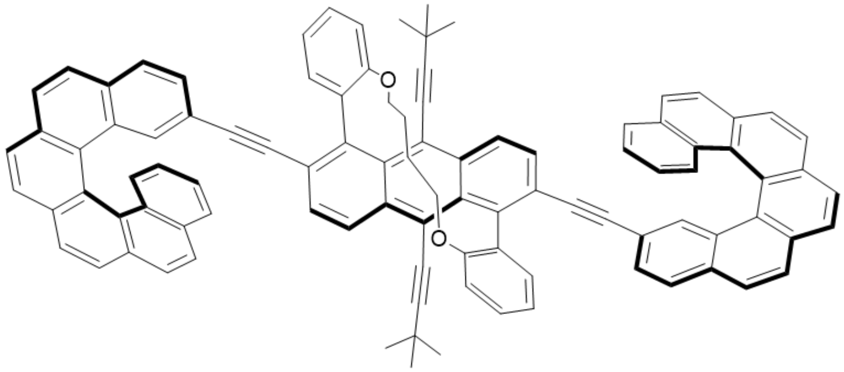

Following the general procedure, 55 mg of *M*-**T$_8$** and 100 mg of *M*-**H** were used to obtain *MMM*-**HT$_8$H** as a dark yellow solid (11 mg, 11% yield). NMR spectra of *MMM*-**HT$_8$H** are consistent the NMR spectra of compound *PPP*-**HT$_8$H**. MALDI-TOF m/z: 1332.314 (M) calcd. for $C_{102}H_{76}O_2$: 1332.584. 1332.584.

***PP*-T₈H**

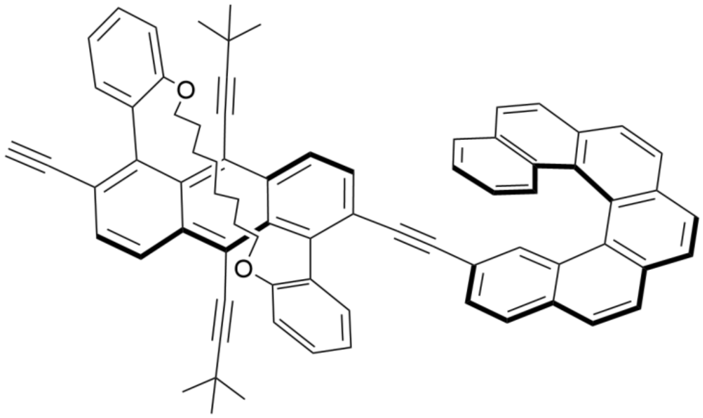

Following the general procedure, 55 mg of *P*-**T**$_8$ and 100 mg of *P*-**H** were used to obtain *PP*- **T**$_8$**H** as a dark yellow solid (13 mg, 16% yield).

^{1}H NMR (500 MHz, $CDCl_3$) δ 8.64 (dd, *J* = 9.1, 4.4 Hz, 2H), 8.03 – 7.99 (m, 4H), 7.97 (d, *J* = 4.2 Hz, 2H), 7.93 (d, *J* = 8.5 Hz, 1H), 7.90 (s, 1H), 7.84 (d, *J* = 8.4 Hz, 2H), 7.81 (s, 1H), 7.66 (d, *J* = 8.2 Hz, 2H), 7.58 – 7.50 (m, 1H), 7.49 – 7.41 (m, 1H), 7.39 (dd, *J* = 7.4, 1.7 Hz, 1H), 7.31 (dd, *J* = 7.4, 1.7 Hz, 1H), 7.29 – 7.27 (m, 1H), 7.08 (td, *J* = 7.4, 1.0 Hz, 1H), 6.97 – 6.91 (m, 2H), 6.82 (dd, *J* = 8.2, 1.5 Hz, 1H), 6.79 (d, *J* = 7.9 Hz, 1H), 6.70 (ddd, *J* = 8.5, 6.9, 1.4 Hz, 2H), 4.11 – 3.88 (m, 1H), 3.78 (dq, *J* = 13.7, 4.2 Hz, 2H), 3.74 – 3.67 (m, 1H), 2.97 (s, 1H), 1.19 (s, 9H), 1.11 (s, 9H), 0.89 (dd, *J* = 7.6, 2.0 Hz, 4H), 0.48 (d, *J* = 10.8 Hz, 8H). **^{13}C NMR** (126 MHz, $CDCl_3$) δ 157.93 (d, *J* = 2.0 Hz), 141.67, 140.52, 134.79, 134.68, 133.28, 132.47, 131.60, 131.57, 131.54, 131.40, 131.17, 130.15, 129.76, 129.71, 129.61, 129.30, 129.00, 128.89, 128.75, 128.45, 128.18, 127.96, 127.84, 127.72, 127.68, 127.55, 127.47, 127.42, 127.39, 127.35, 127.22, 126.99, 126.29, 125.99, 124.78, 124.22, 123.77, 122.08, 120.31, 120.26, 119.97, 119.82, 119.71, 115.91, 115.84, 112.22, 111.97, 95.31, 90.24, 84.24, 81.60, 78.93, 69.49, 69.36, 34.54, 31.59, 31.07, 30.98, 30.35, 29.71, 29.59, 29.38, 28.75, 28.68, 27.01, 26.98, 26.17, 24.27. MALDI-TOF m/z: 1006.349 (M) calcd. for $C_{76}H_{62}O_2$: 1006.474. UV-vis (THF): λ_{max} (ε) = 309 nm (49713 M^{-1} cm^{-1}), 474 nm (14101 M^{-1} cm^{-1}).

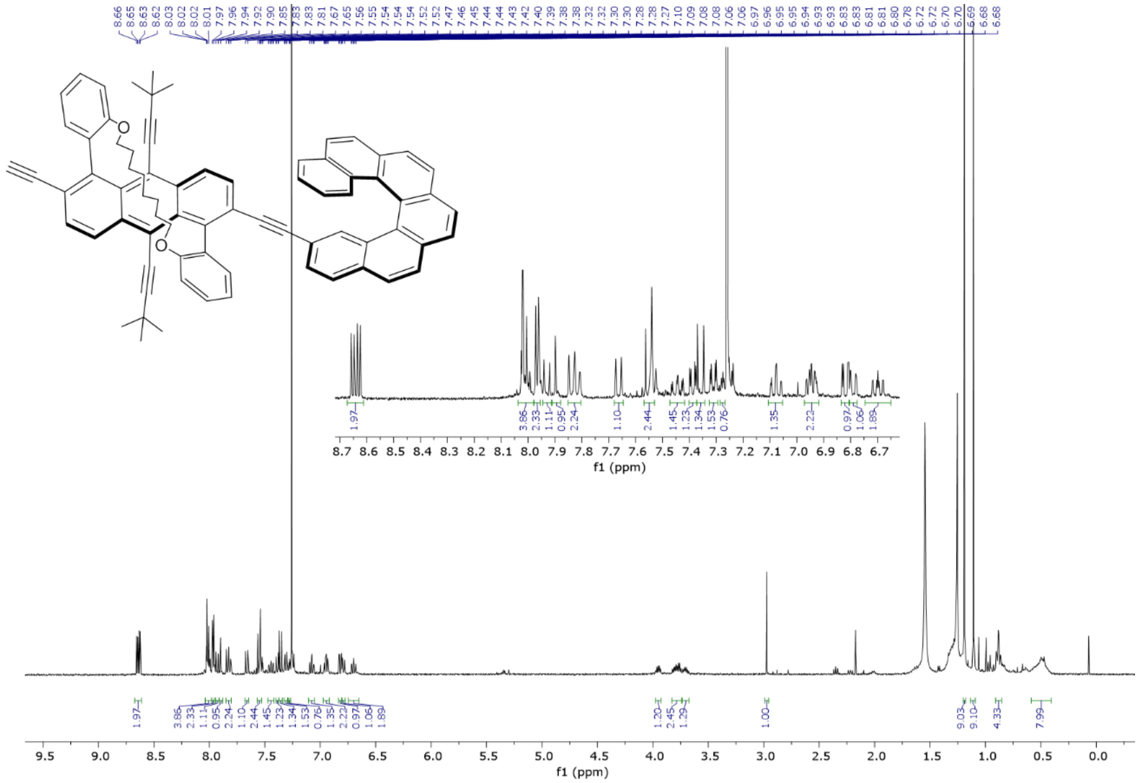

Figure S 26. ^{1}H NMR (500 MHz) of *PP*-**T$_8$H** in CDCl$_3$, measured at 298 K.

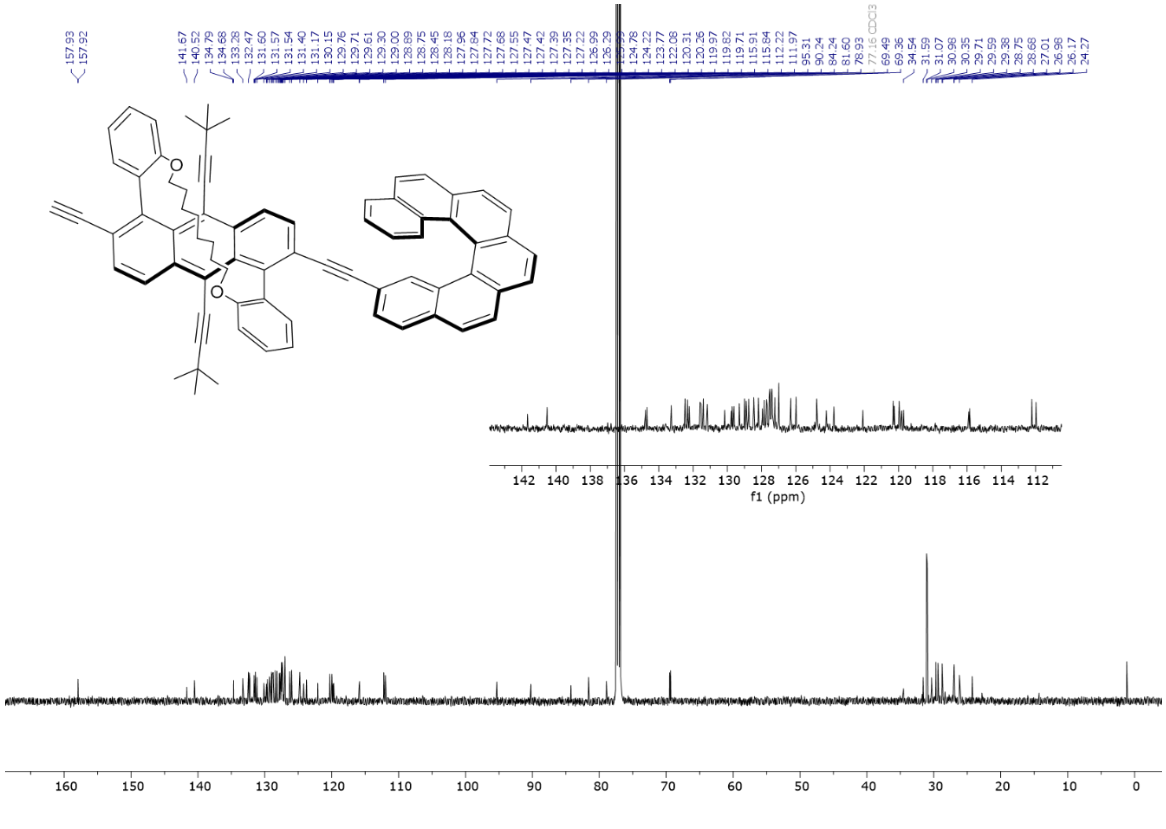

Figure S 27. ^{13}C NMR (126 MHz) of *PP*-**T$_8$H** in CDCl$_3$, measured at 298 K.

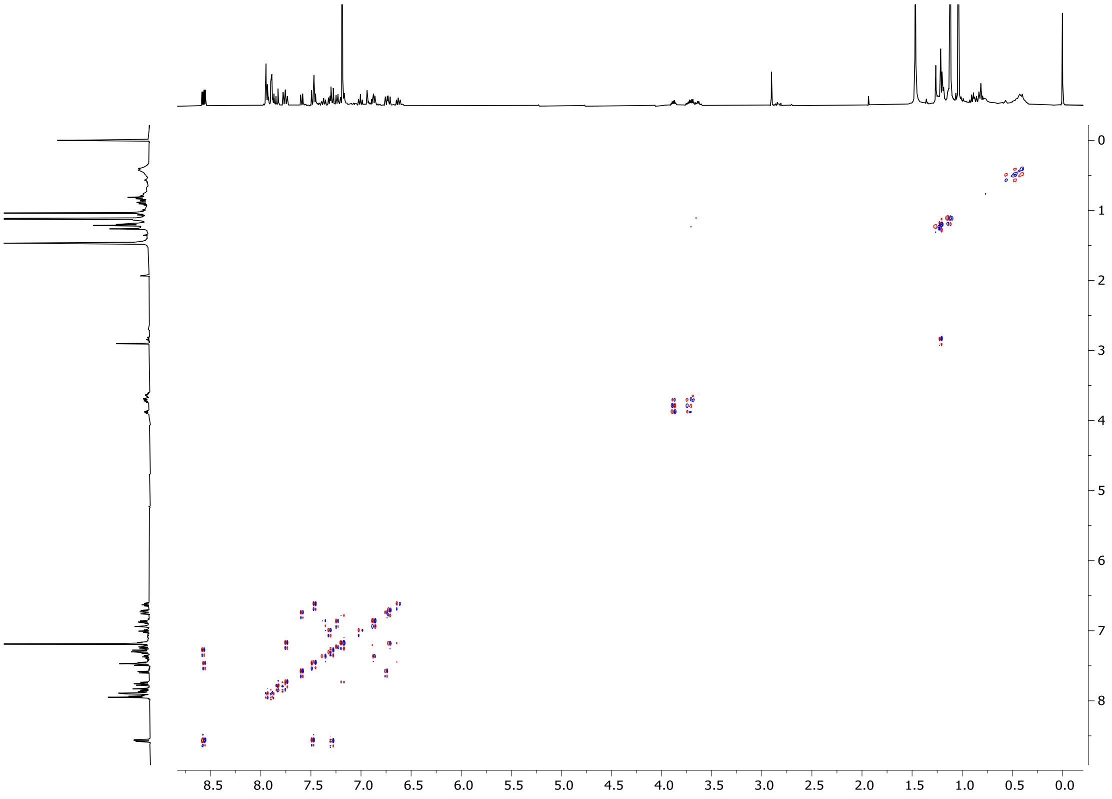

Figure S 28. COSY (500 MHz) of *PP*-$\mathbf{T_8H}$ in $CDCl_3$, measured at 298 K.

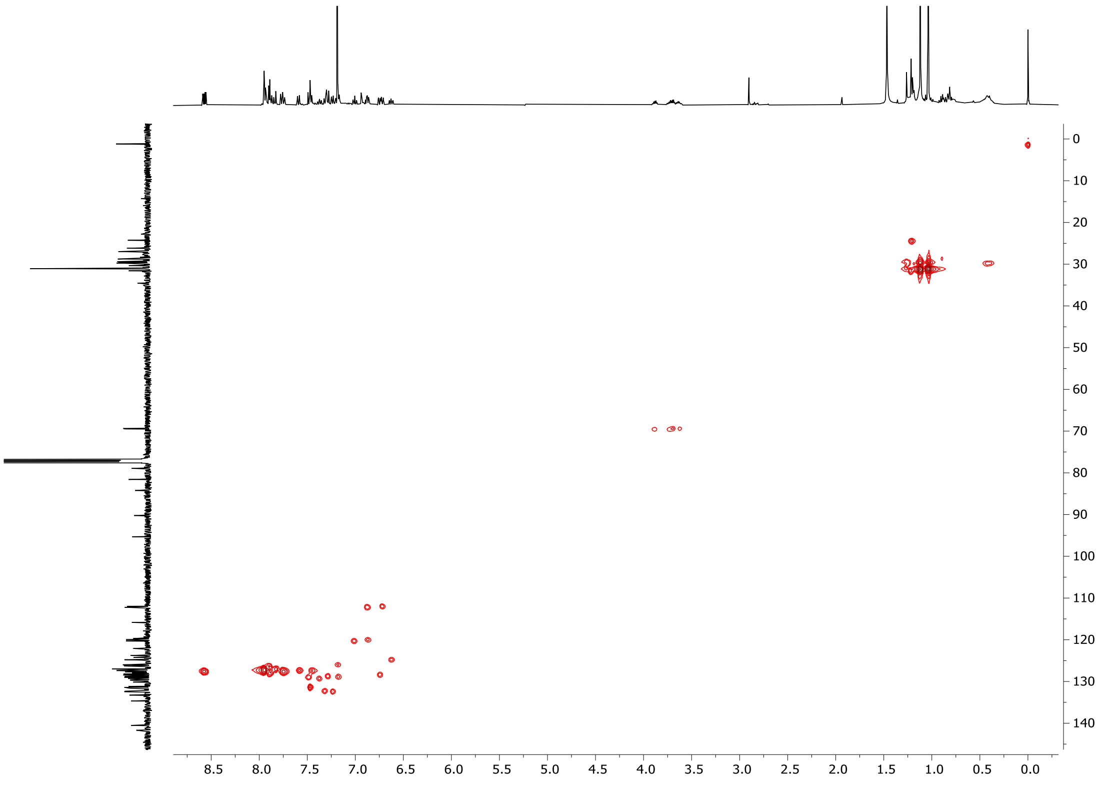

Figure S 29. HSQC (500 MHz) of *PP*-$\mathbf{T_8H}$ in $CDCl_3$, measured at 298 K.

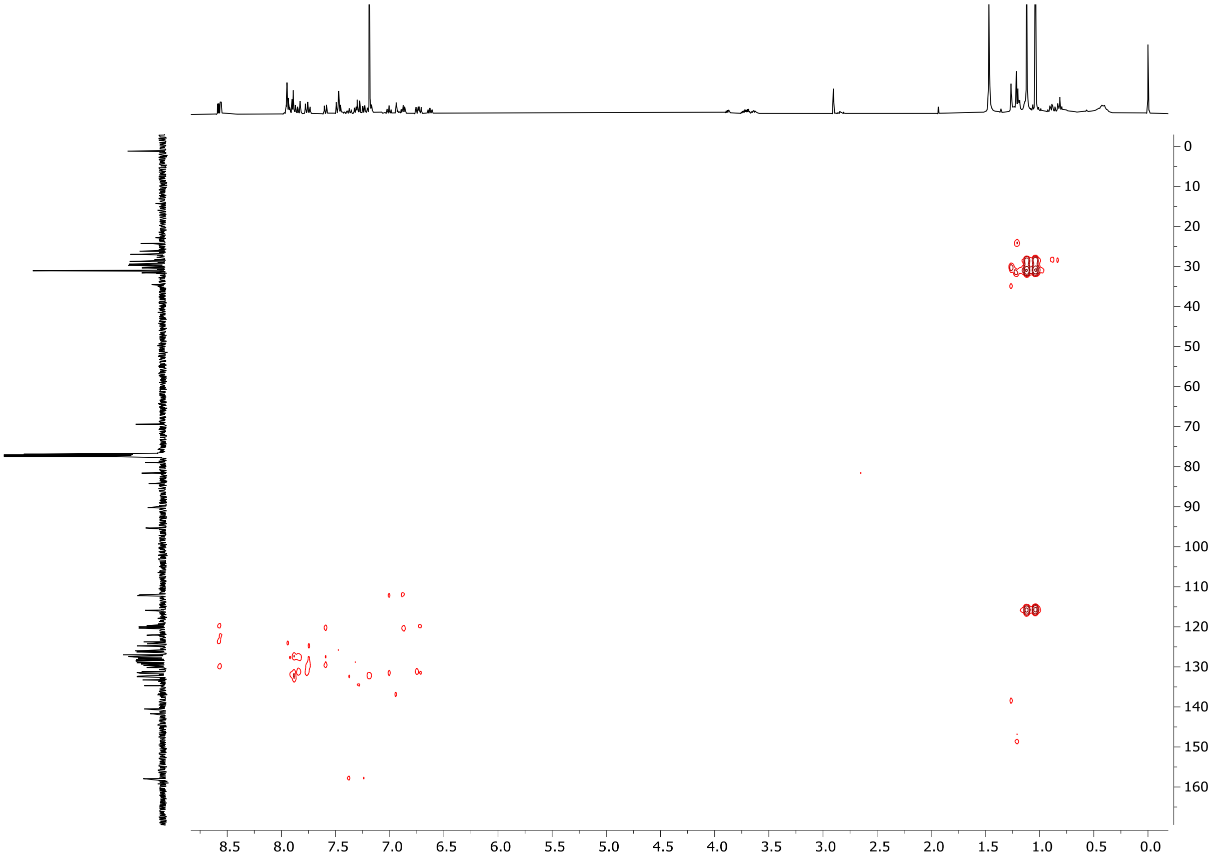

Figure S 30. HMBC (500 MHz) of *PP*-**T8H** in $CDCl_3$, measured at 298 K.

MM-T8H

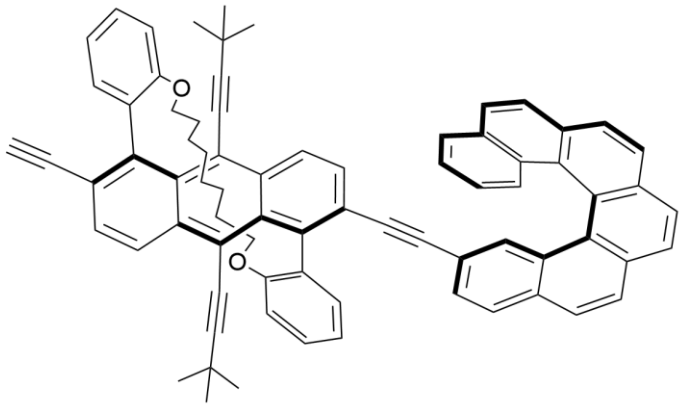

Following the general procedure, 55 mg of *M*-**T8** and 100 mg of *M*-**H** were used to obtain *MM*-**T8H** as a dark yellow solid (10 mg, 13% yield). NMR spectra of *MM*-**HT8H** are consistent the NMR spectra of compound *PP*-**HT8H**. MALDI-TOF m/z: 1006.558 (M) calcd. for $C_{76}H_{62}O_2$: 1006.474.

MPM-**HT**$_{8}$**H**

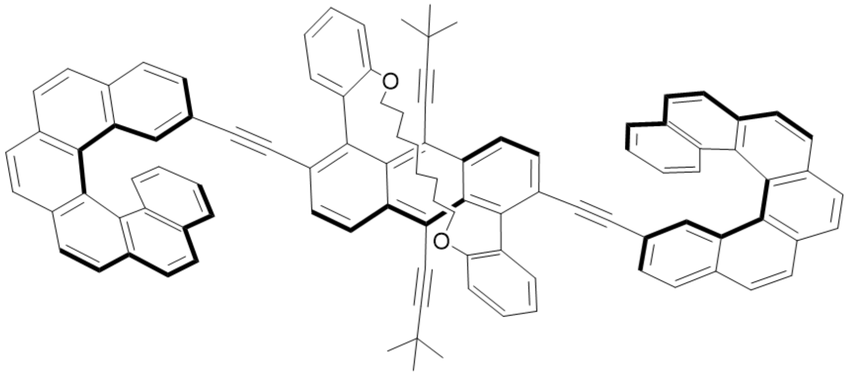

Following the general procedure, 55 mg of *P*-**T**$_{8}$ and 100 mg of *M*-**H** were used to obtain *MPM*-**HT**$_{8}$**H** as a dark yellow solid (16 mg, 15% yield).

^{1}**H NMR** (500 MHz, $CDCl_3$) δ 8.63 (d, *J* = 9.1 Hz, 2H), 8.07 – 8.03 (m, 4H), 8.02 (d, *J* = 1.4 Hz, 2H), 8.00 (d, *J* = 3.1 Hz, 4H), 7.96 (d, *J* = 8.2 Hz, 2H), 7.91 (d, *J* = 8.5 Hz, 2H), 7.84 (d, *J* = 8.6 Hz, 2H), 7.80 (dd, *J* = 8.0, 1.4 Hz, 2H), 7.67 (d, *J* = 8.2 Hz, 2H), 7.60 – 7.57 (m, 2H), 7.55 – 7.52 (m, 2H), 7.39 (d, *J* = 9.1 Hz, 2H), 7.20 (dd, *J* = 6.7, 1.3 Hz, 2H), 7.18 (d, *J* = 7.4 Hz, 4H), 6.86 (dd, *J* = 8.2, 1.5 Hz, 2H), 6.79 (d, *J* = 8.2 Hz, 2H), 6.74 (td, *J* = 7.4, 1.0 Hz, 2H), 6.67 (ddd, *J* = 8.5, 6.8, 1.4 Hz, 2H), 3.85 – 3.72 (m, 4H), 1.31 (m, 6H), 1.17 (s, 18H), 0.73 – 0.54 (m, 8H).

^{13}**C NMR** (126 MHz, $CDCl_3$) δ 157.77, 140.32, 134.45, 133.17, 131.05, 129.86, 129.62, 129.49, 128.70, 128.54, 128.17, 128.09, 127.87, 127.72, 127.55, 127.42, 127.39, 127.26, 127.22, 127.09, 126.90, 126.85, 126.22, 125.76, 124.58, 124.12, 123.34, 120.15, 120.03, 119.41, 115.43, 111.60, 94.86, 90.12, 78.86, 69.28, 31.60, 30.89, 30.51, 29.72, 29.61, 29.26, 28.57, 26.99, 22.67. MALDI-TOF m/z: 1332.533 (M) calcd. for $C_{102}H_{76}O_2$: 1332.584. UV-vis (THF): λ_{max} (ε) = 235 nm (104065 M^{-1} cm^{-1}), 485 nm (16668 M^{-1} cm^{-1}).

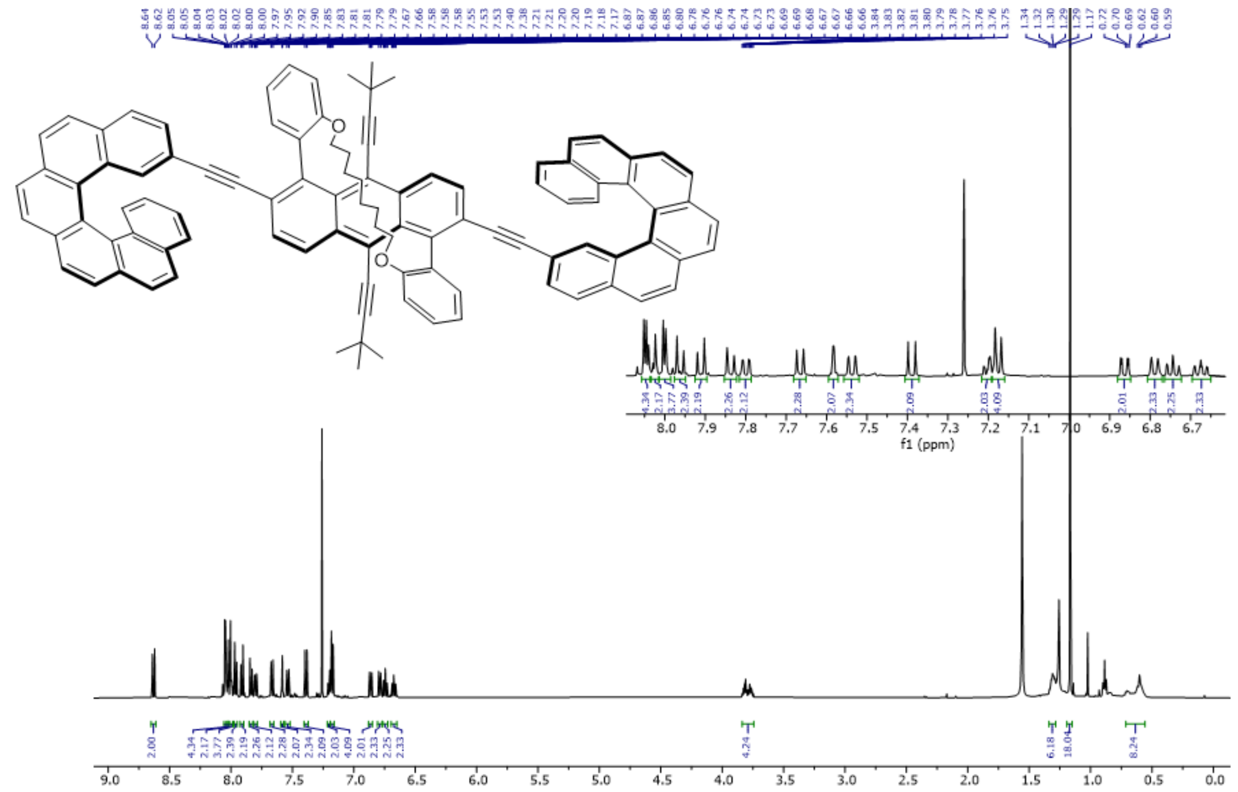

Figure S 31. ^{1}H NMR (500 MHz) of *MPM*-**HT$_8$H** in $CDCl_3$, measured at 298 K.

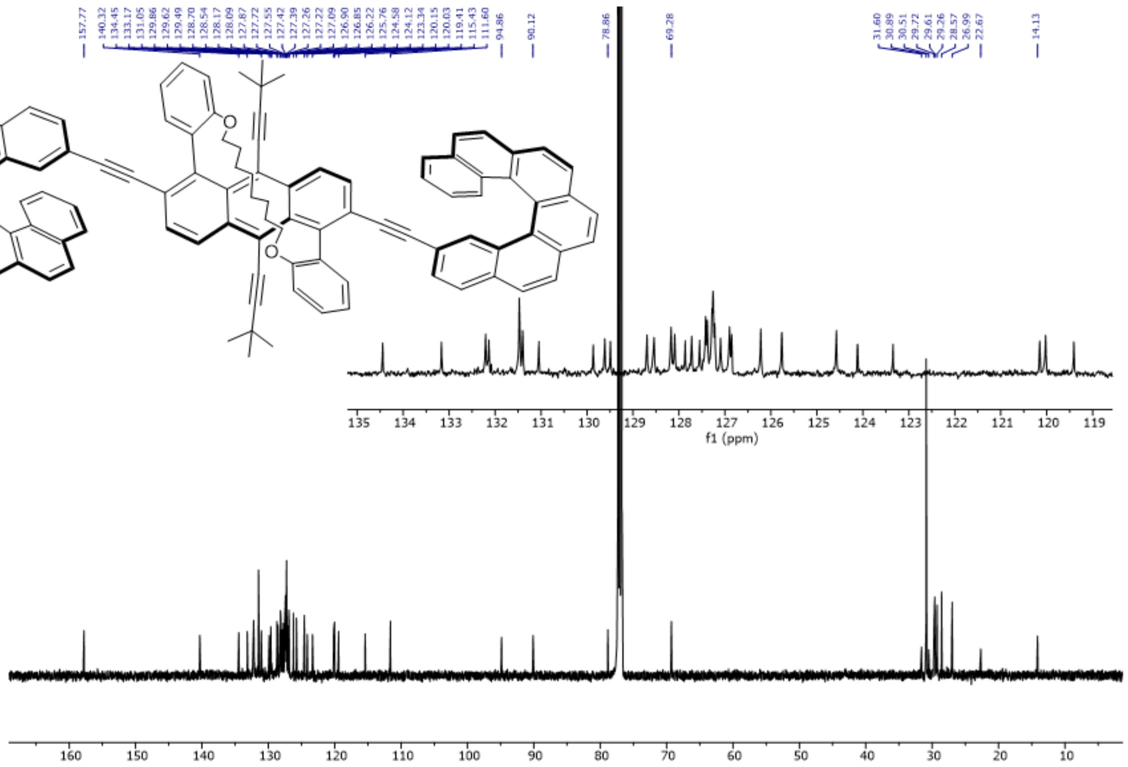

Figure S 32. ^{13}C NMR (126 MHz) of *MPM*-**HT$_8$H** in $CDCl_3$, measured at 298 K.

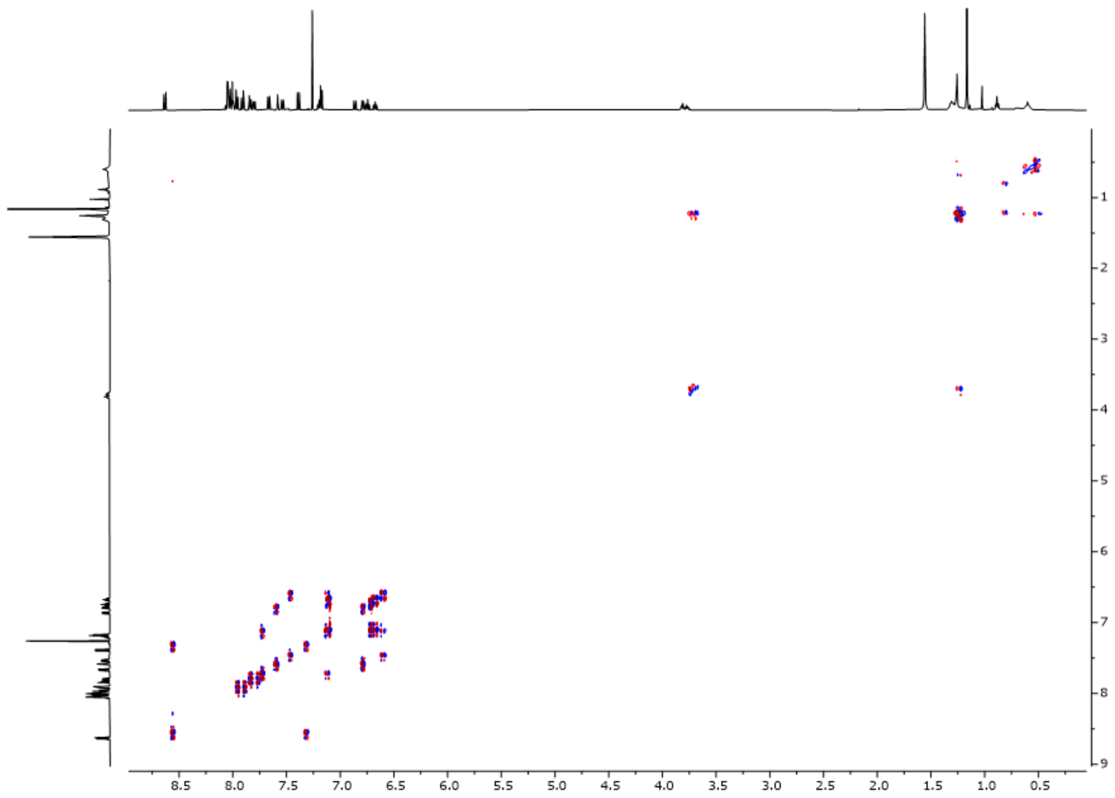

Figure S 33. COSY (500 MHz) of *MPM*-**HT₈H** in $CDCl_3$, measured at 298 K.

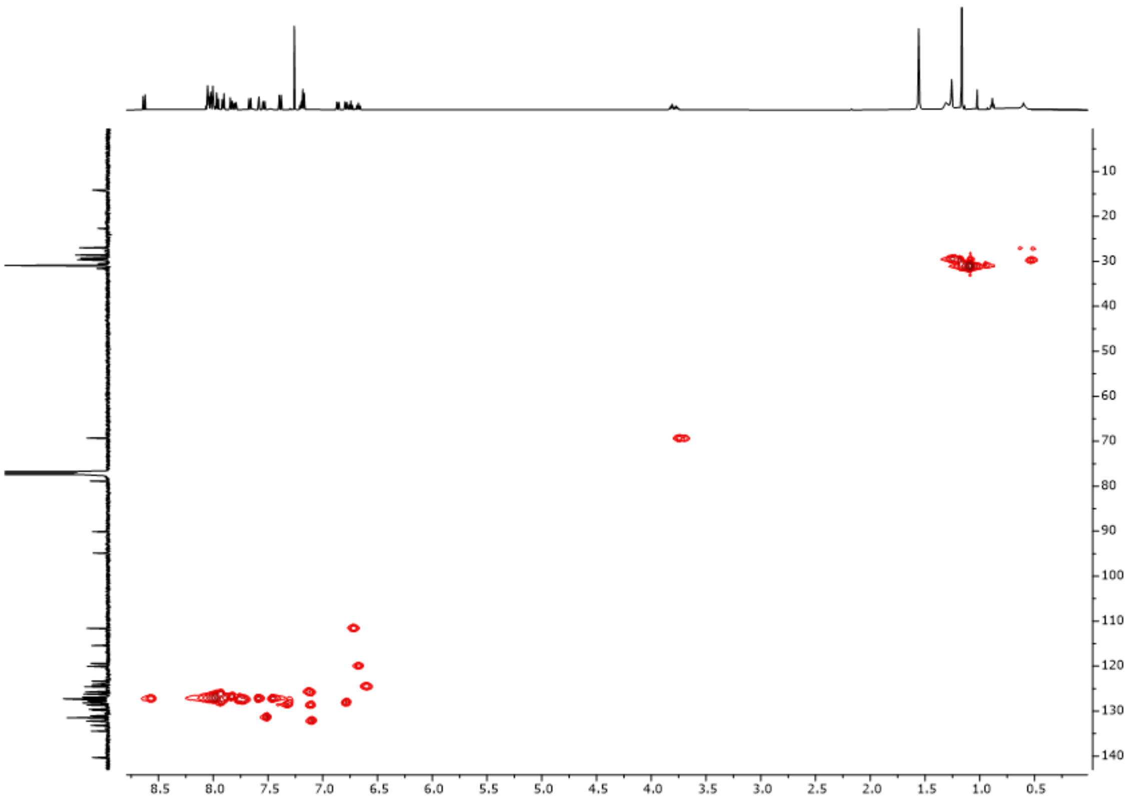

Figure S 34. HSQC (500 MHz) of *MPM*-**HT₈H** in $CDCl_3$, measured at 298 K.

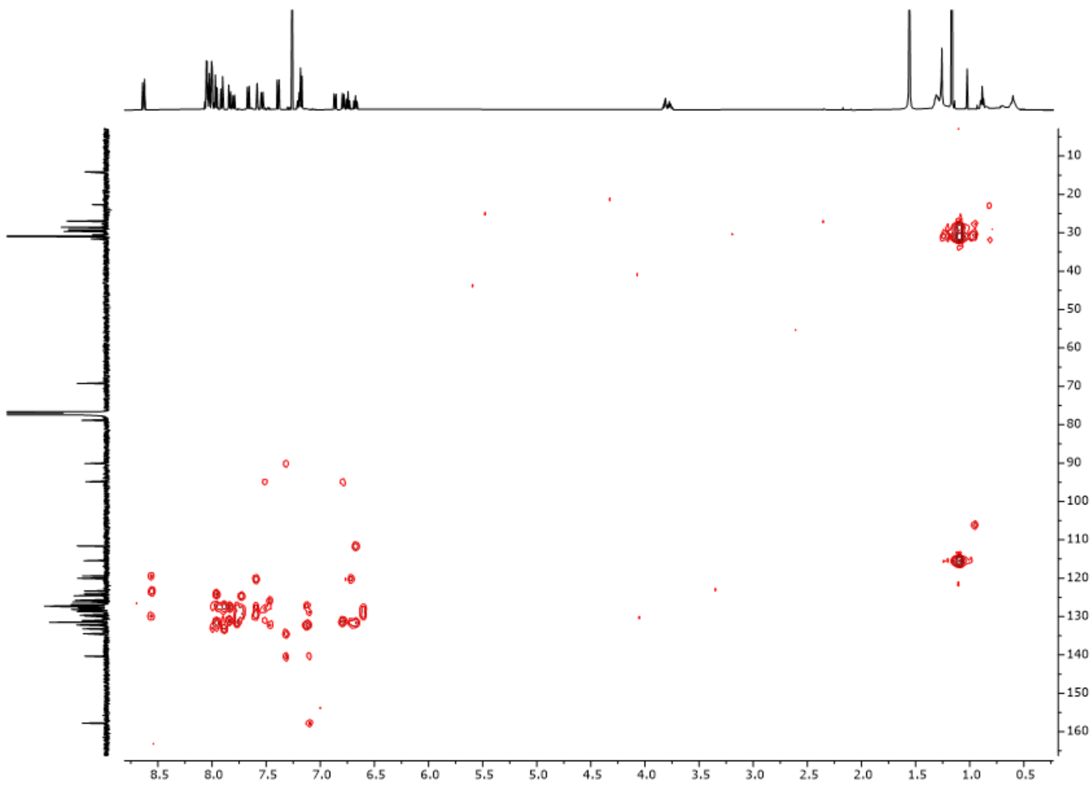

Figure S 35. HMBC (500 MHz) of *MPM*-**HT$_8$H** in $CDCl_3$, measured at 298 K.

S3.2 Chiral HPLC separation of racemic twisted acenes and 2-bromo[6]helicene

- Racemic 2-bromo[6]helicene was resolved using semi-preparative CHIRALPAK-IG column using 35% dichloromethane/hexane as the eluent, the *P*-enantiomer was eluted first followed by the *M*-enantiomer (Figure 39).
- *PPP*-**HT$_4$H**, *PPP*-**HT$_8$H** and *MPM*-**HT$_4$H** were loaded into semi-preparative CHIRALPAK-IG column using 25% dichloromethane/hexane as the eluent, the results show single peak after many cycles.

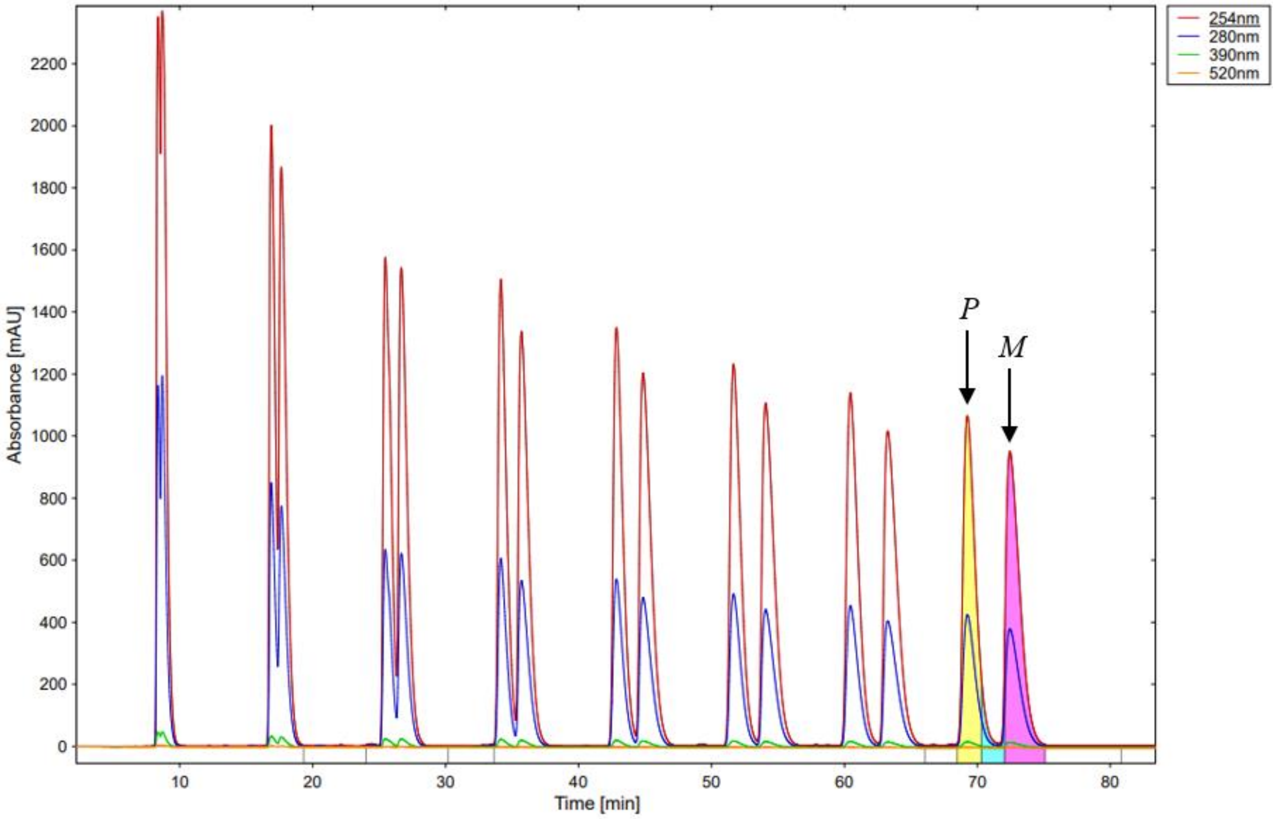

Figure S 36. Chiral HPLC separation of 2-bromo[6]helicene

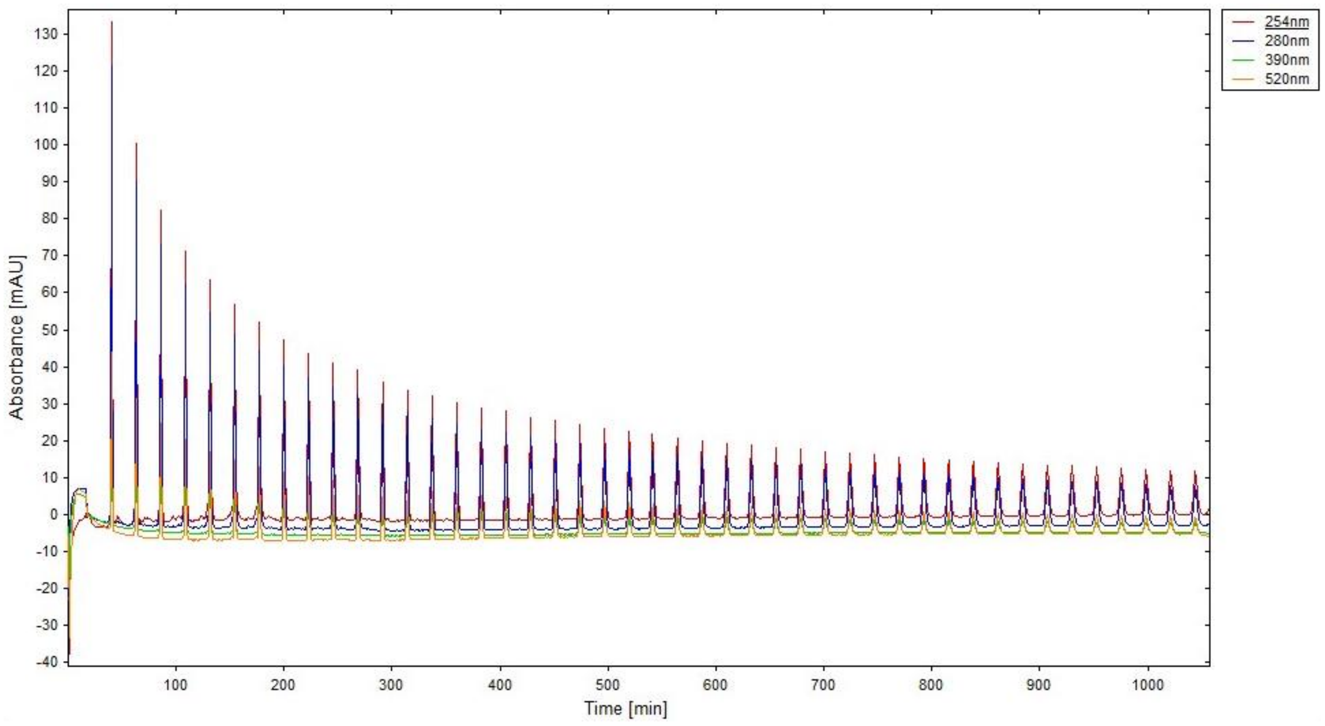

Figure S 37. Chiral HPLC of *PPP*-**HT$_4$H**.

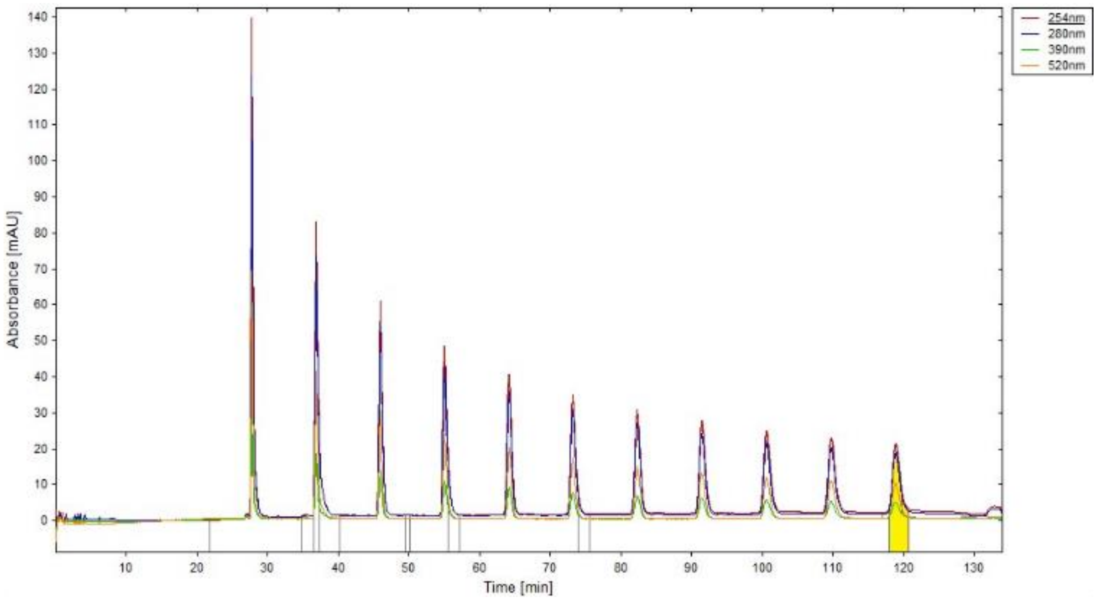

Figure S 38. Chiral HPLC of *MPM*-**HT$_4$H**.

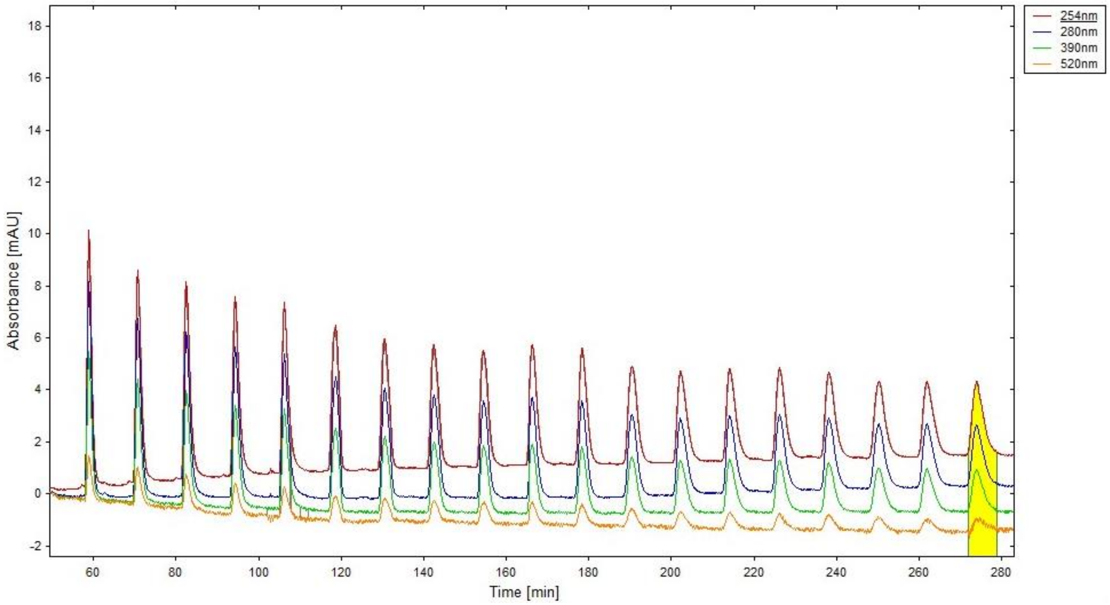

Figure S 39. Chiral HPLC of *PPP*-**HT$_8$H**.

S3.3 Mass spectroscopy: MALDI and LCMS

MMM-**HT₄H**

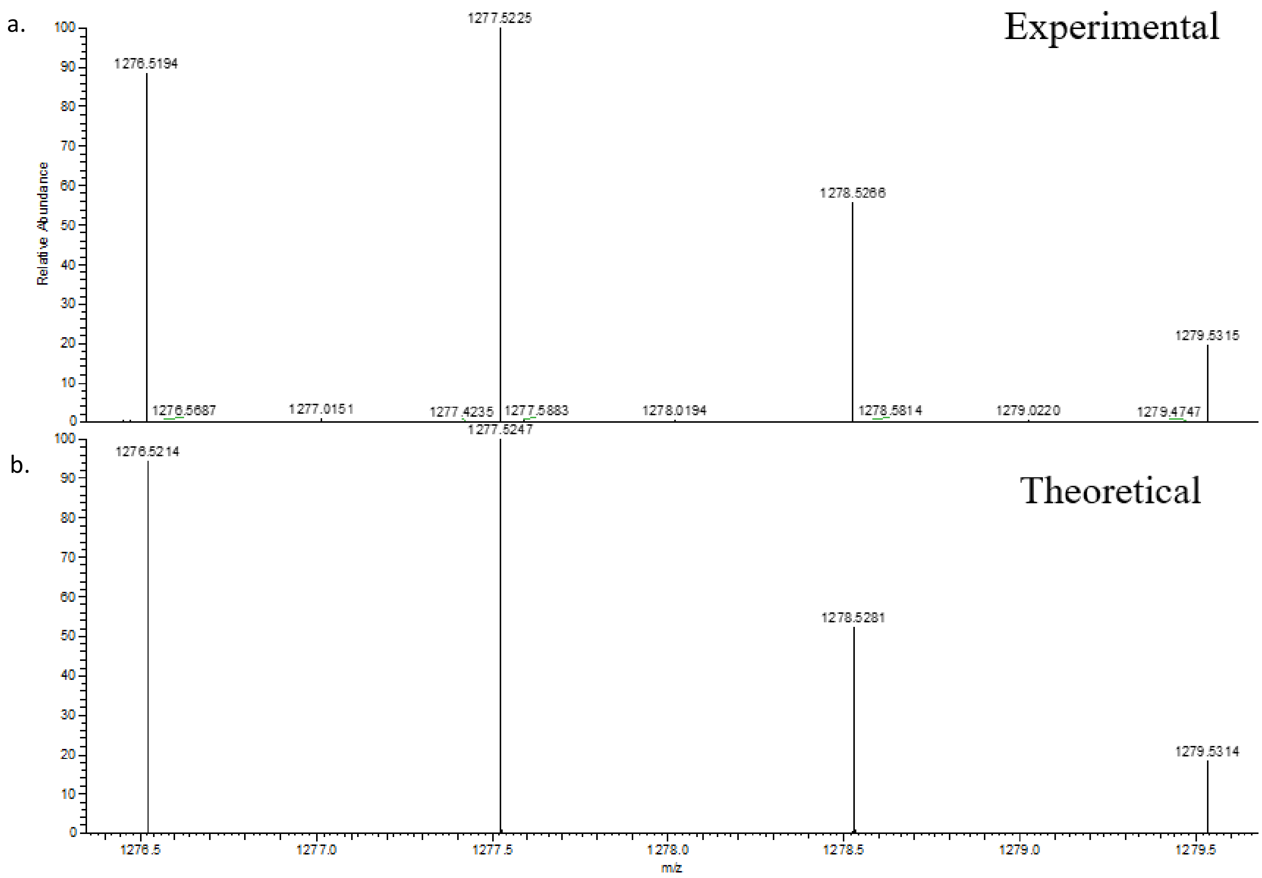

Figure S 40. HRMS spectra of *MMM*-**HT$_4$H**. a. experimental spectrum, b. theoretical spectrum.

MM-**T$_4$H**

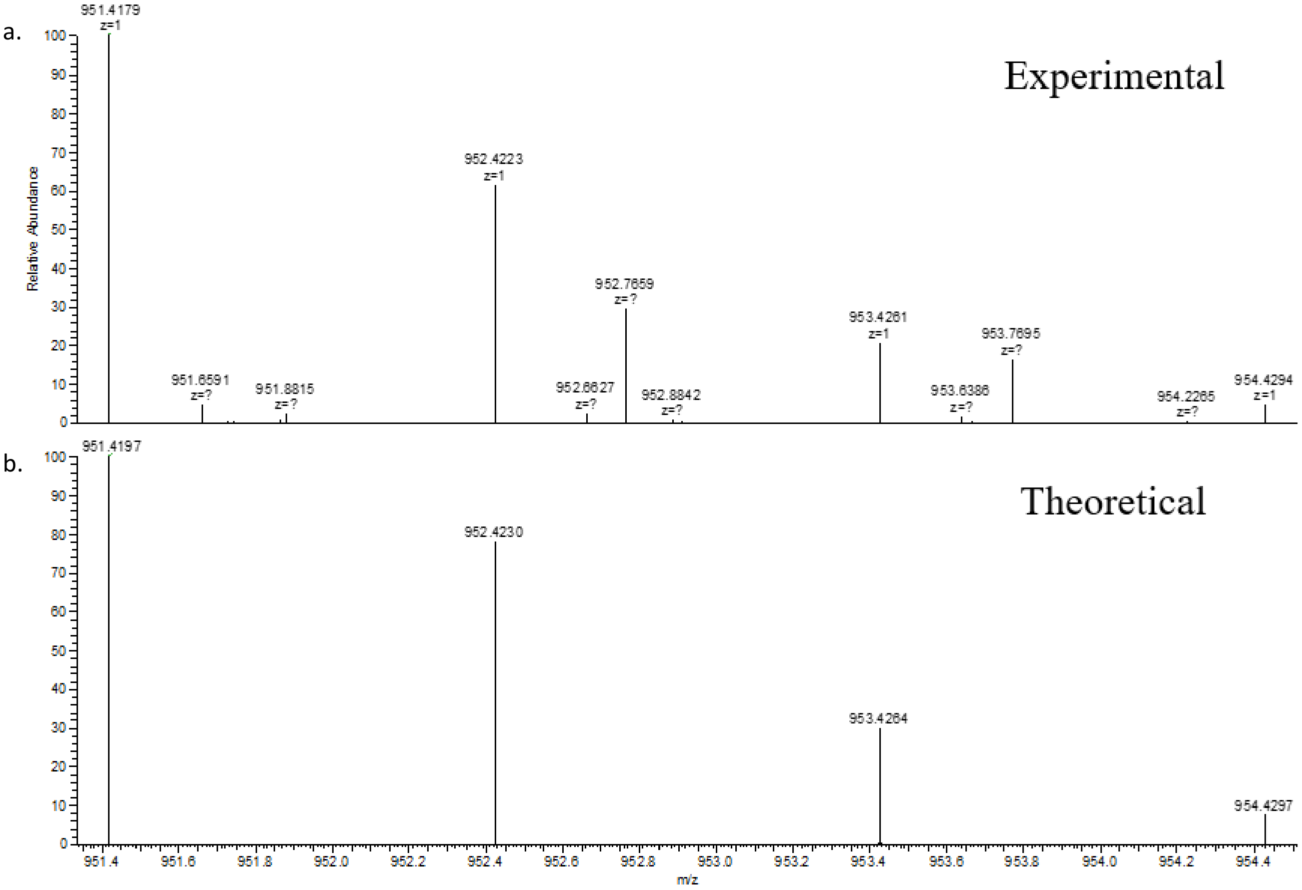

Figure S 41. HRMS spectra of *MM*-**T$_4$H** a. experimental spectrum, b. theoretical spectrum.

PPP-**HT$_4$H**

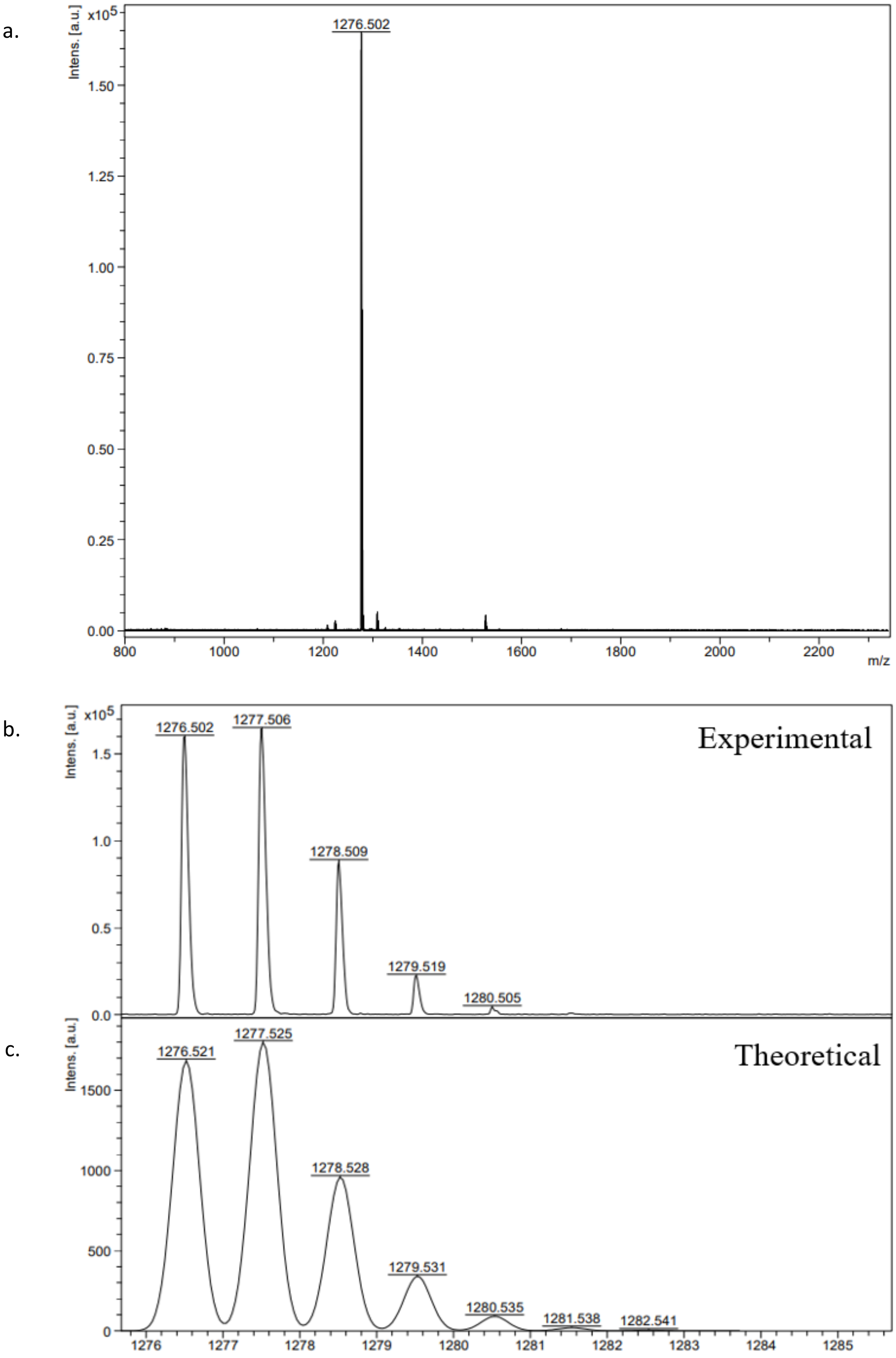

Figure S 42. MALDI-TOF spectrum of *PPP*-**HT$_4$H**. a. full spectrum, b. experimental spectrum, c. theoretical spectrum.

PP-**T$_4$H**

a.

b.

c.

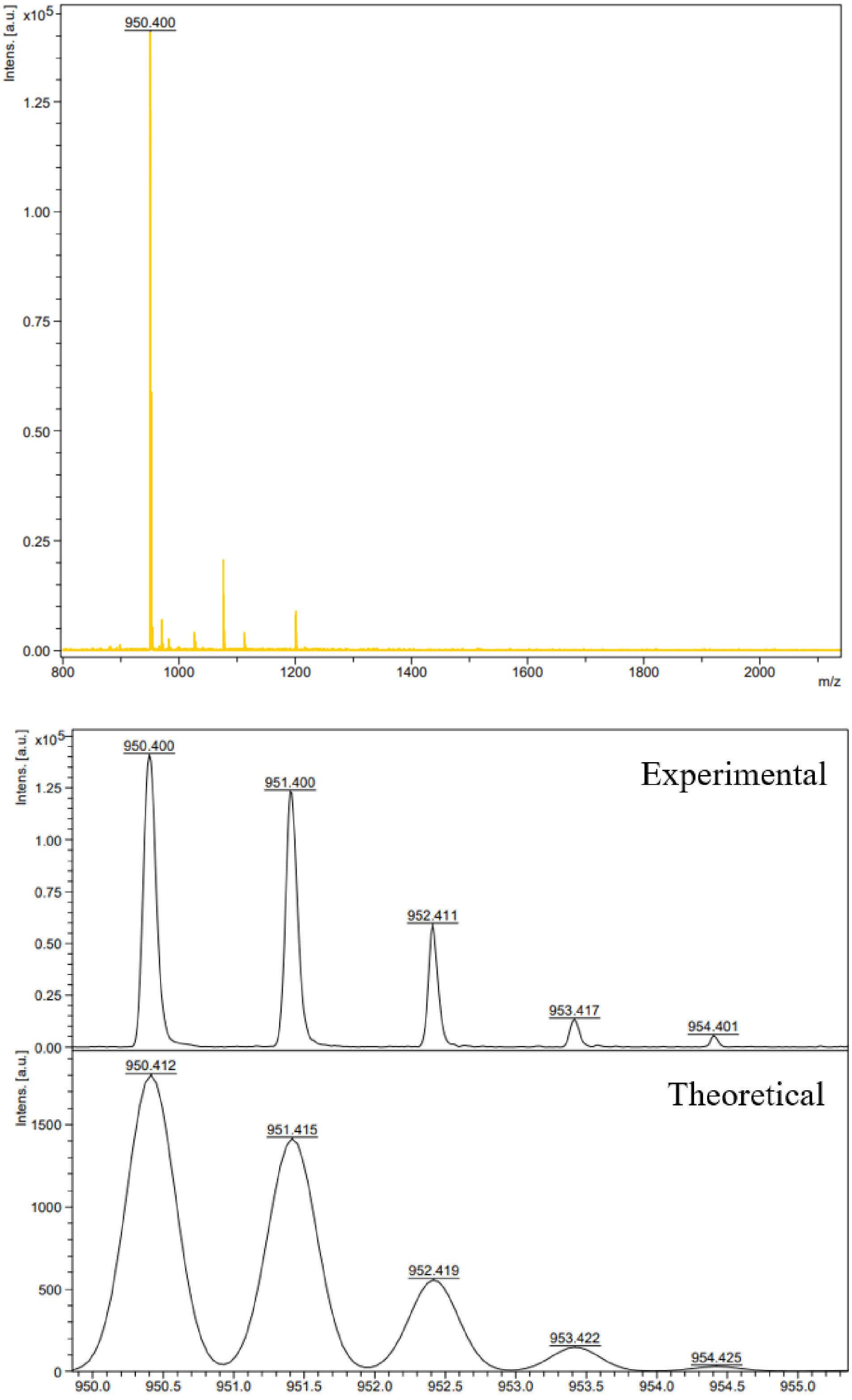

Figure S 43. MALDI-TOF spectra of *PP*-**T$_4$H**. a. full spectrum, b. experimental spectrum, c. theoretical spectrum.

MPM-**HT₄H**

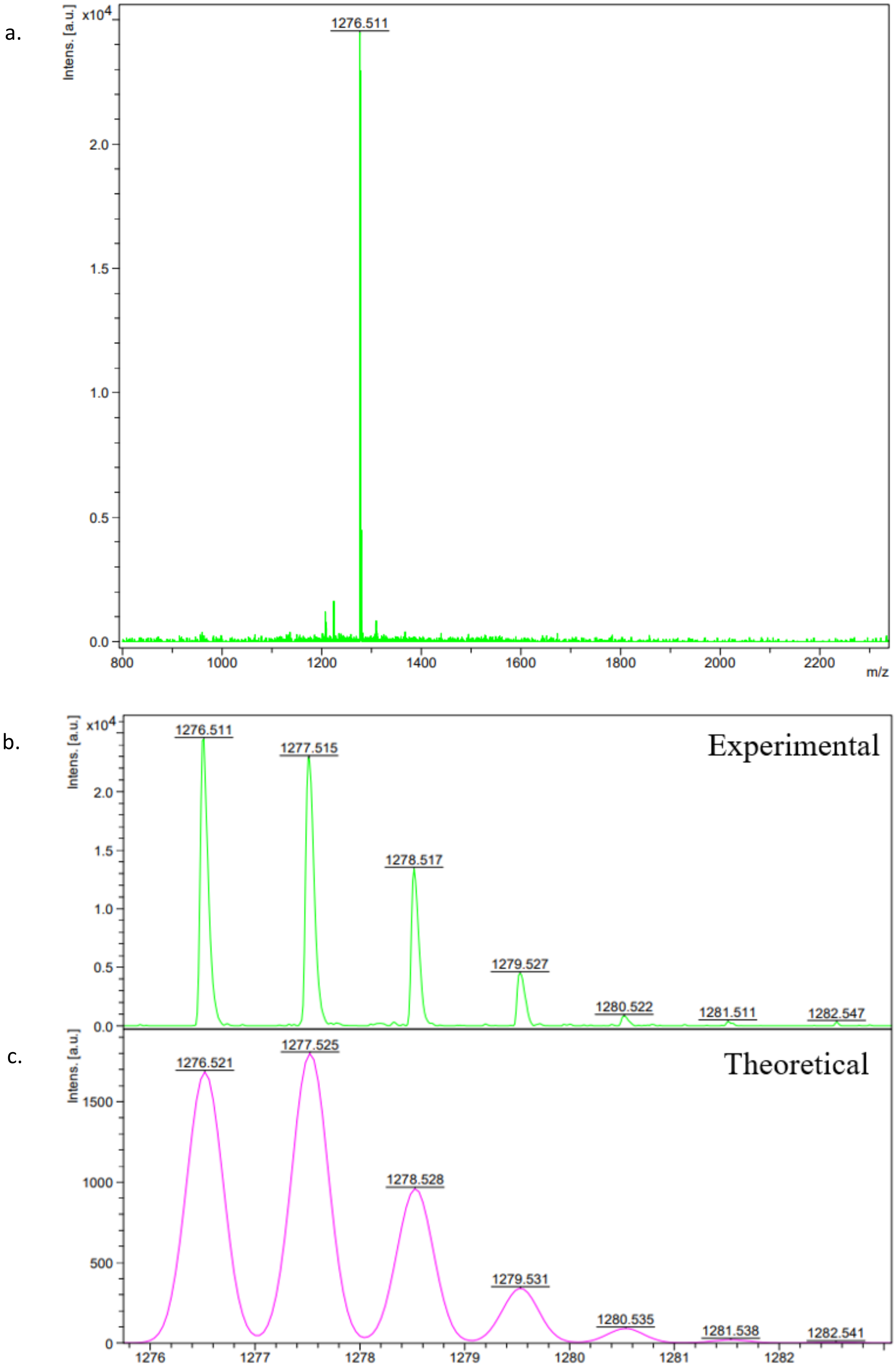

Figure S 44. MALDI-TOF spectra of *MPM*-**HT$_4$H**. a. full spectrum, b. experimental spectrum, c. theoretical spectrum.

PM-**T$_4$H**

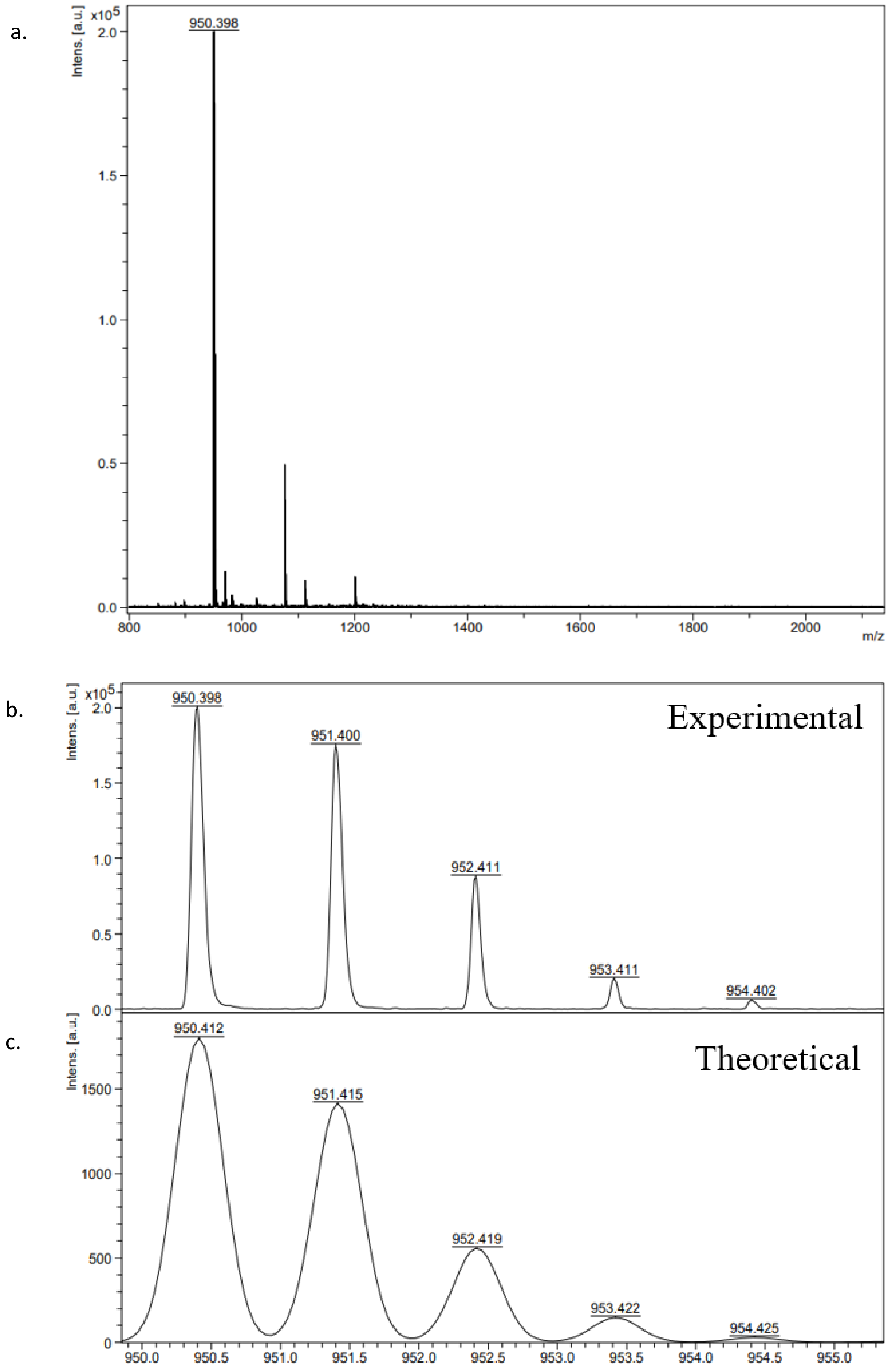

Figure S 45. MALDI-TOF spectrum of *PM*-**T$_4$H**. a. full spectrum, b. experimental spectrum, c. theoretical spectrum.

PPP-**HT**$_{8}$**H**

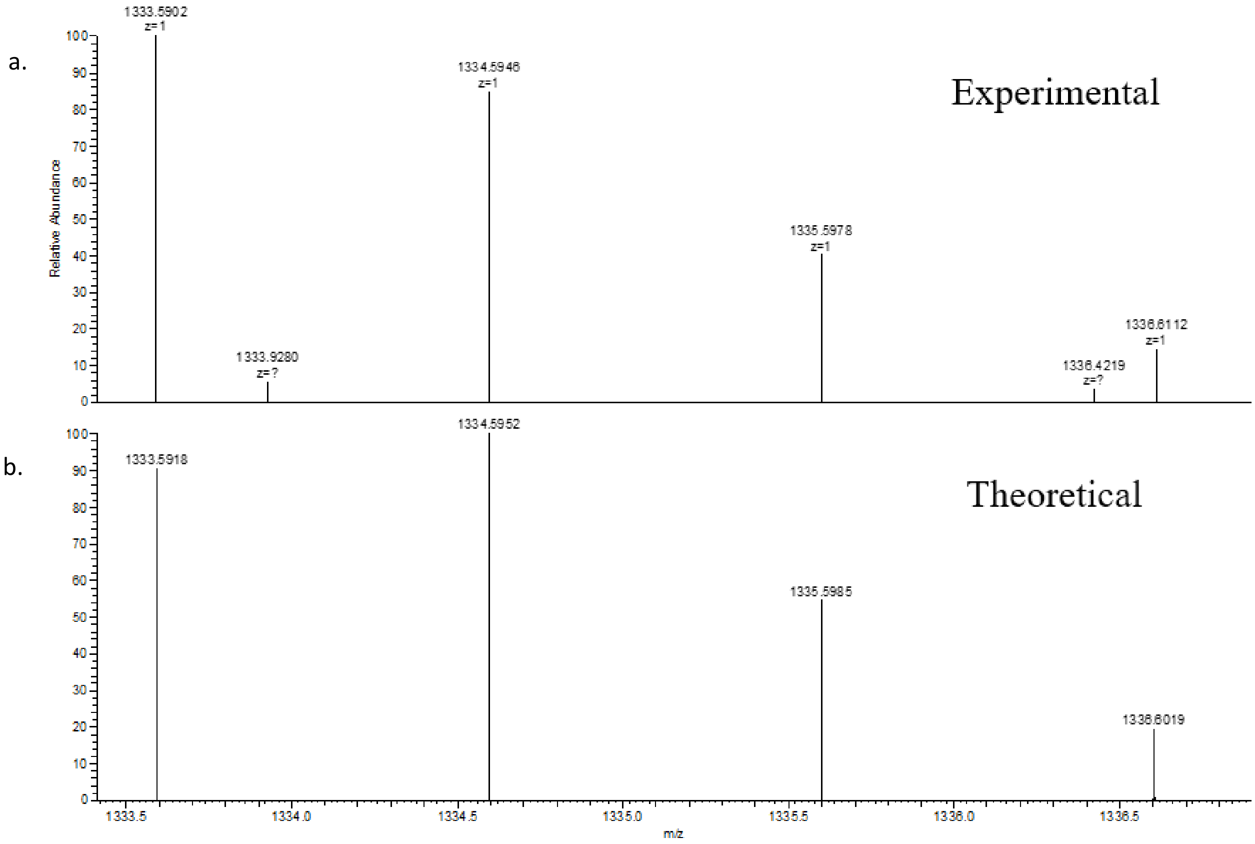

Figure S 46. HRMS spectra of *PPP*-**HT**$_{8}$**H**. a. experimental spectrum, b. theoretical spectrum.

PP-**T**$_8$**H**

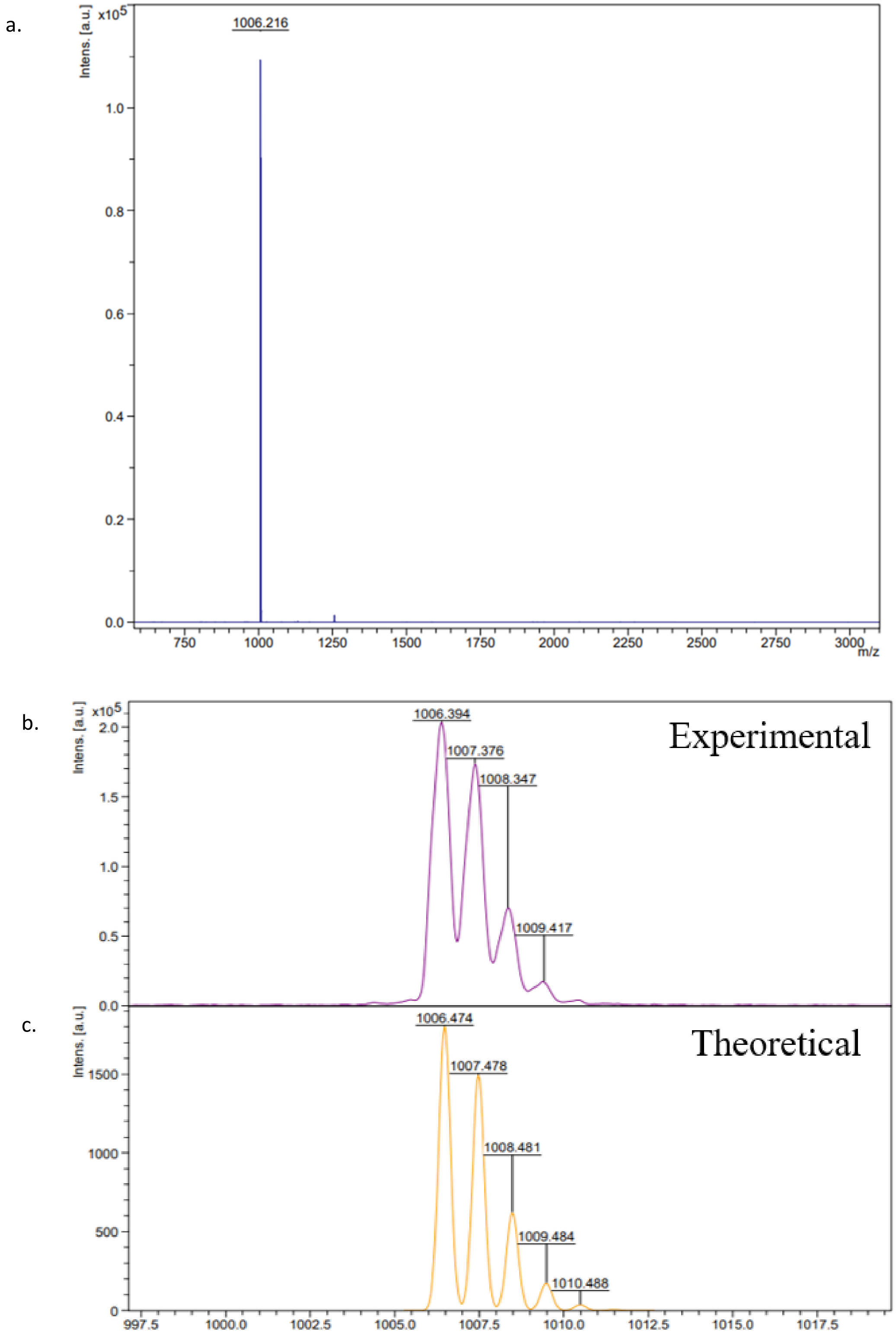

Figure S 47. MALDI-TOF spectra of *PP*-**T**$_8$**H**. a. full spectrum, b. experimental spectrum, c. theoretical spectrum.

MMM-**HT$_8$H**

a.

b.

c.

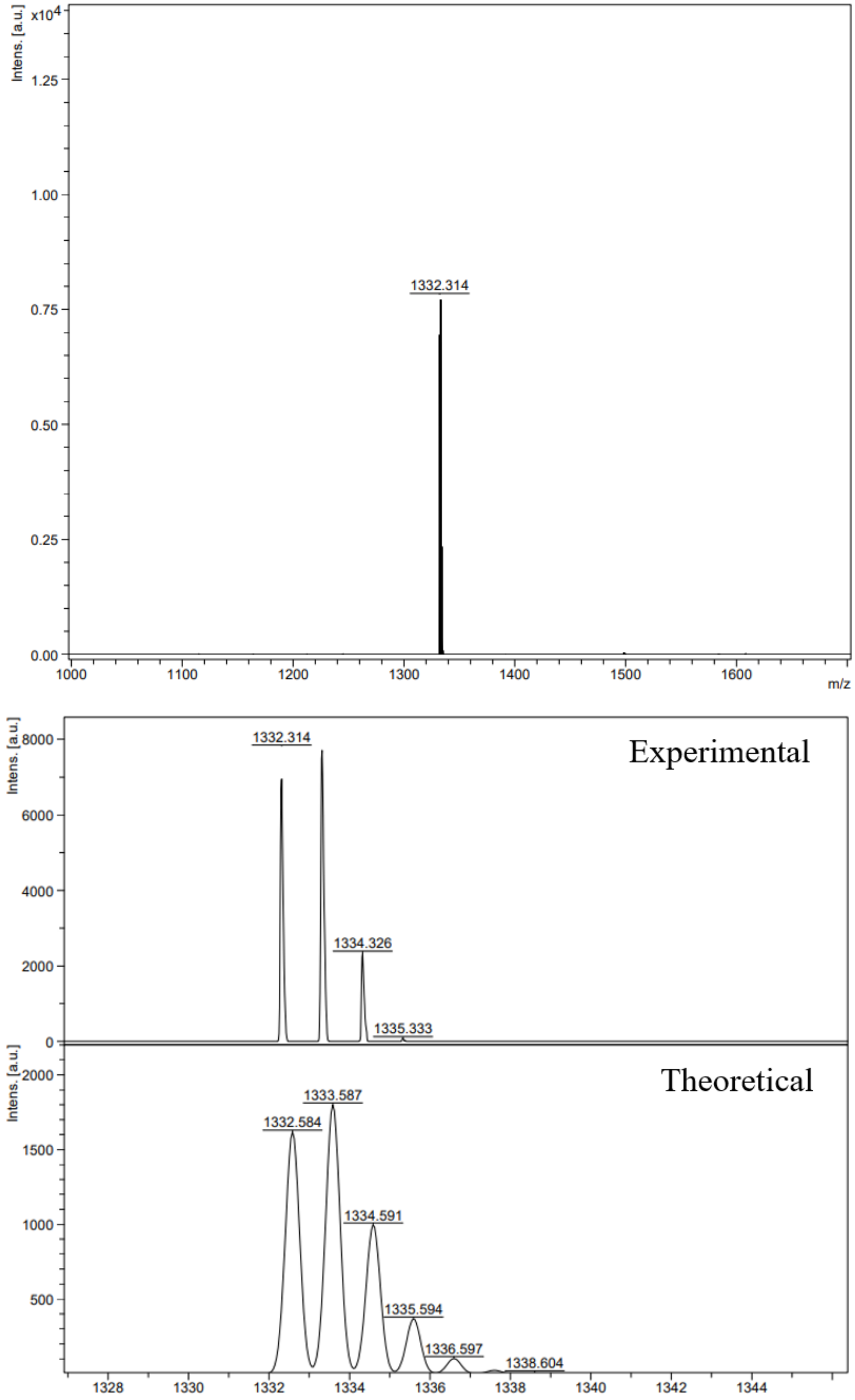

Figure S 48. MALDI-TOF spectra of *MMM*-**HT$_8$H**. a. full spectrum, b. experimental spectrum, c. theoretical spectrum.

MM-**T_8H**

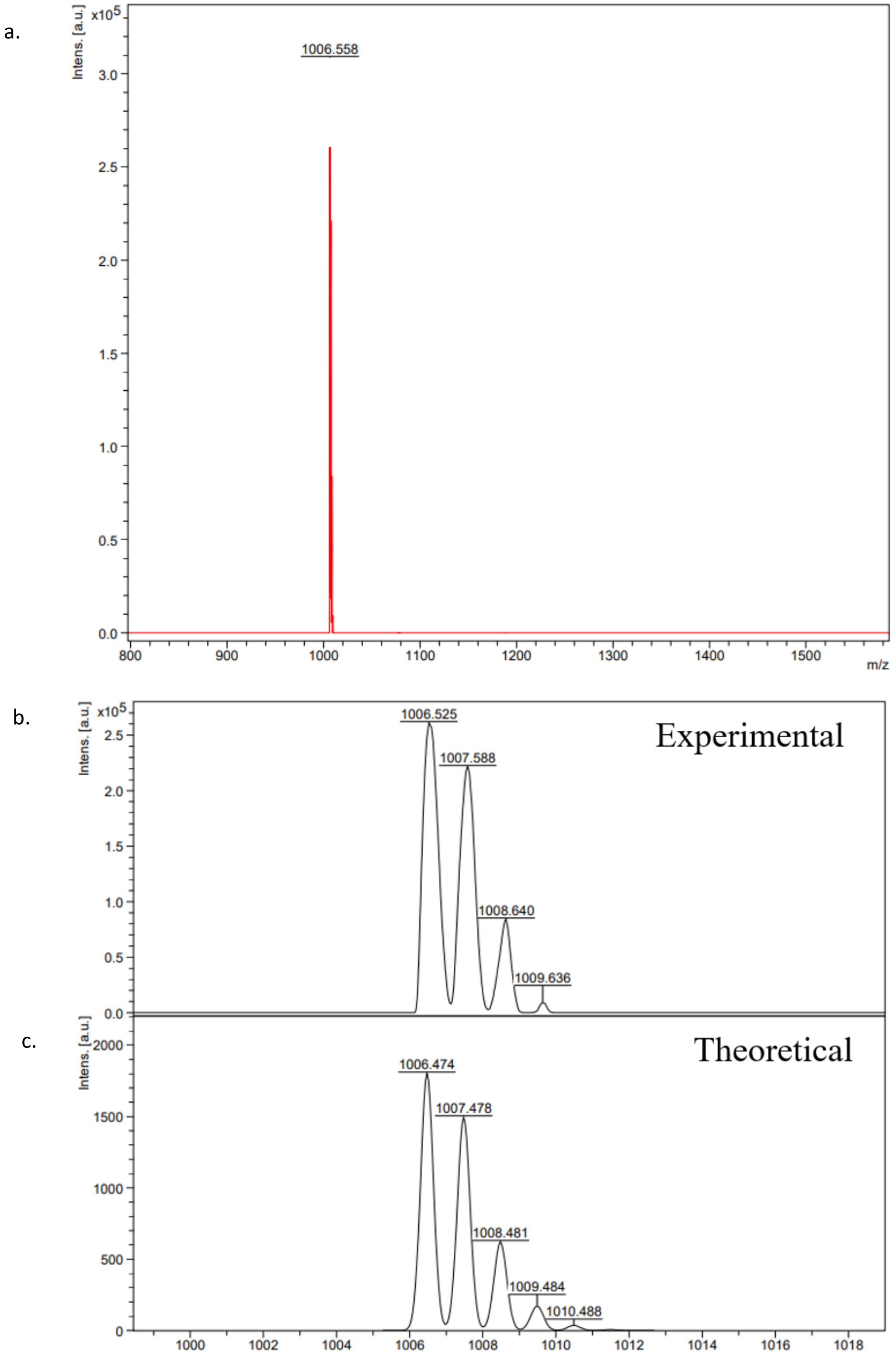

Figure S 49. MALDI-TOF spectra of *MM*-**T_8H**. a. full spectrum, b. experimental spectrum, c. theoretical spectrum.

MPM-**HT₈H**

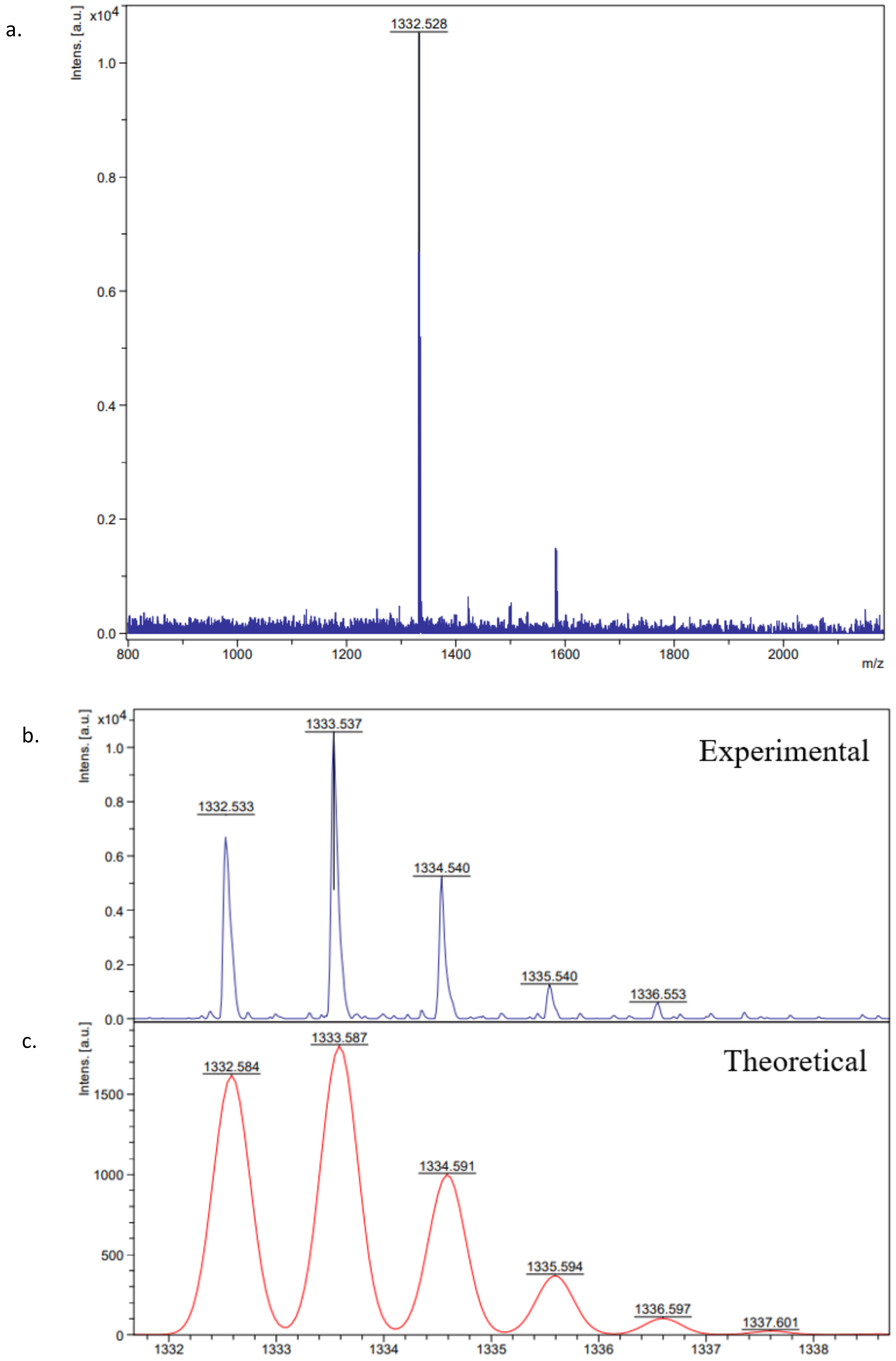

Figure S 50. MALDI-TOF spectra of *MPM*-**HT₈H**. a. full spectrum, b. experimental spectrum, c. theoretical spectrum.

S4 Photophysical properties

S4.1 UV-vis absorption spectra

All photophysical studies were performed with dilute solutions of the compounds keeping the absorbance from the lowest energy band in the range of 0.05 to exclude self-absorption.

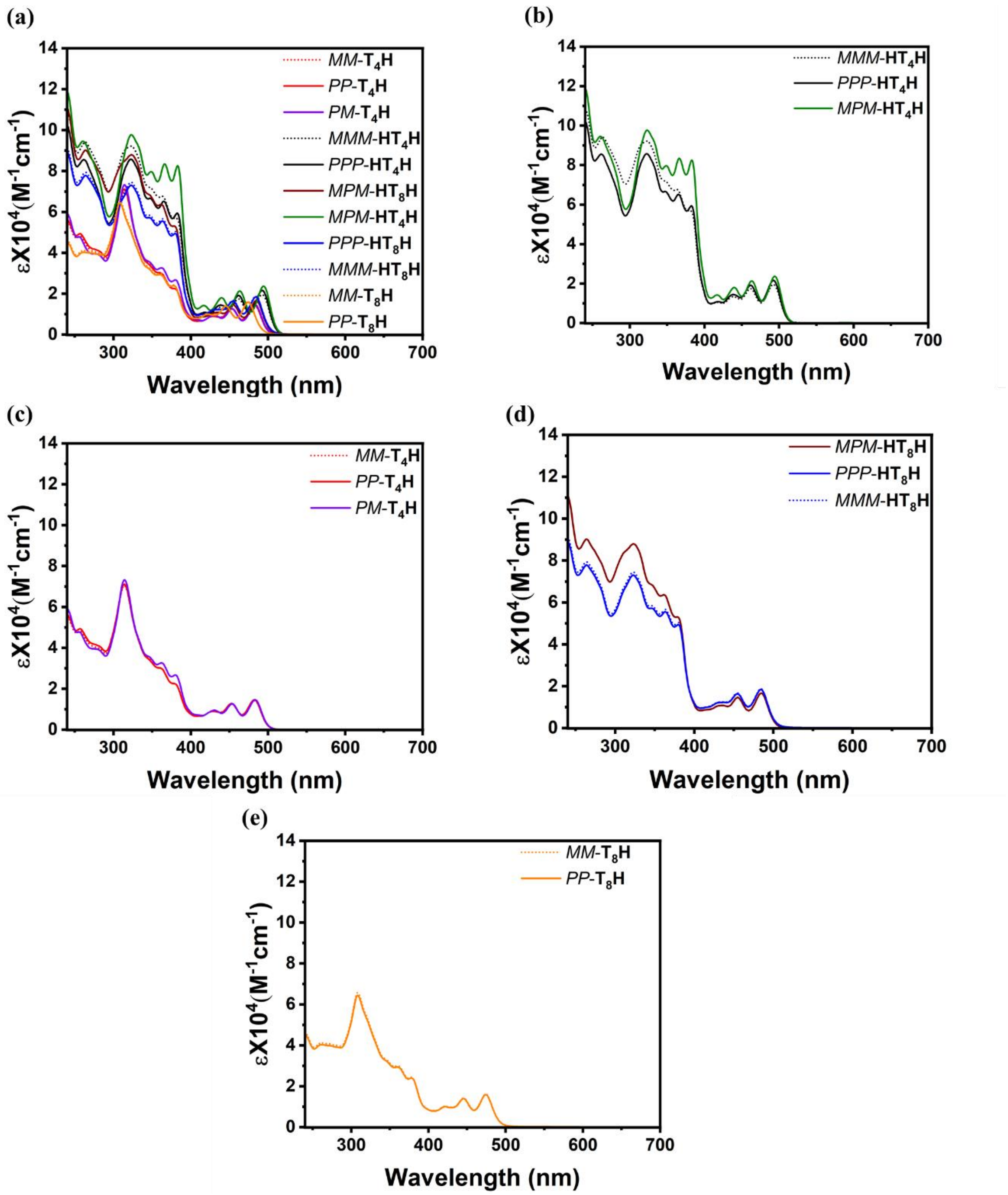

Figure S 51. UV-vis absorption spectra of the compounds indicated on each subfigure dissolved in THF and measured at 298 K. (a) all synthesized helitwistacene molecules (b) **HT$_4$H** helitwistacene molecules (c) **T$_4$H** helitwistacene molecules (d) **HT$_8$H** helitwistacene molecules (e) **T$_8$H** helitwistacene molecules.

S4.1.1 UV-vis absorption spectra of ***MMM***-HT_4H and ***PPP***-HT_8H in different solvents.

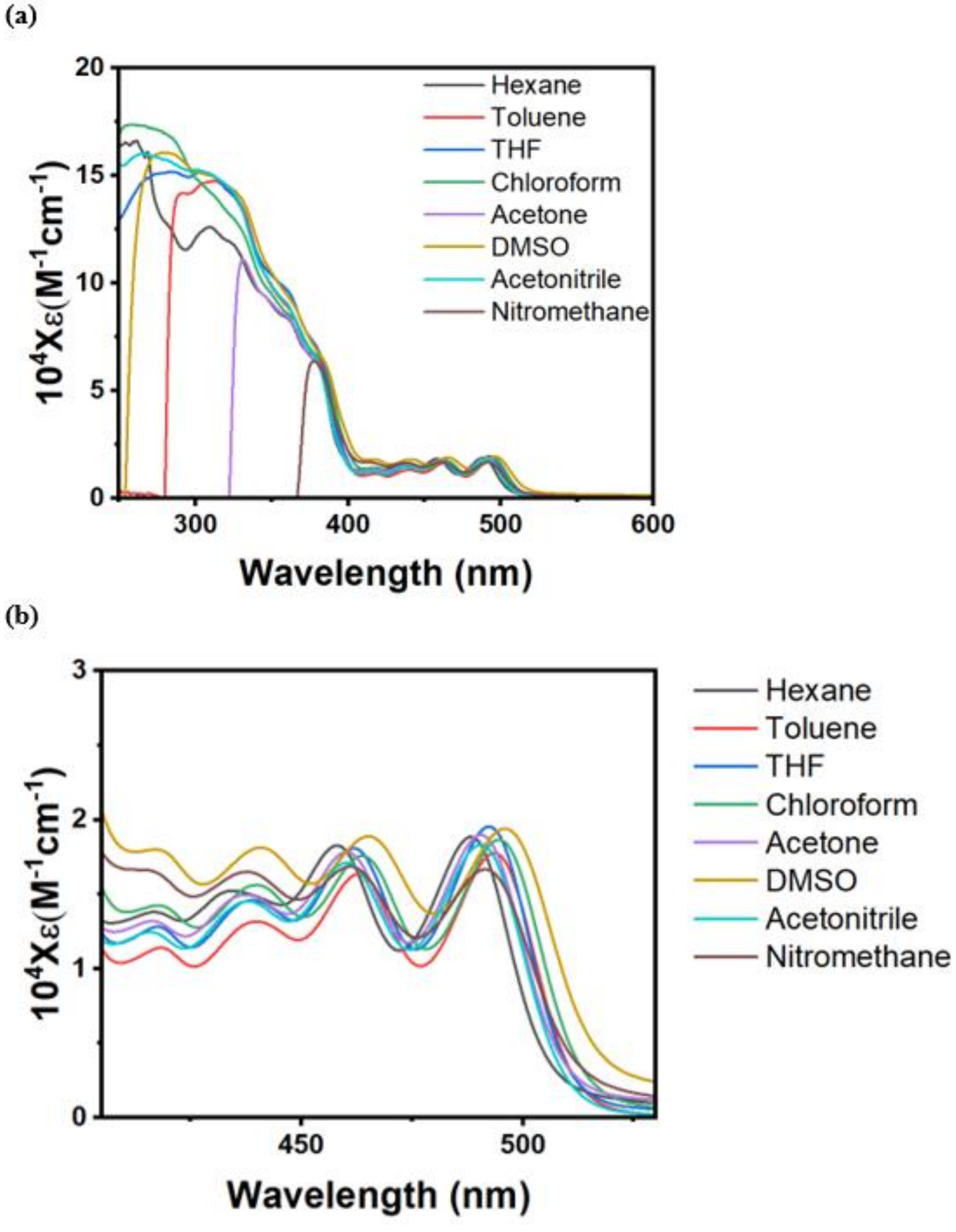

Figure S 52. UV-vis absorption spectrum of *MMM*-**HT_4H** dissolved in different solvents and measured at 298 K. (a) full UV-vis absorption spectrum in different solvents (b) magnification of the lowest energy transition of *MMM*-**HT_4H** in the different solvents.

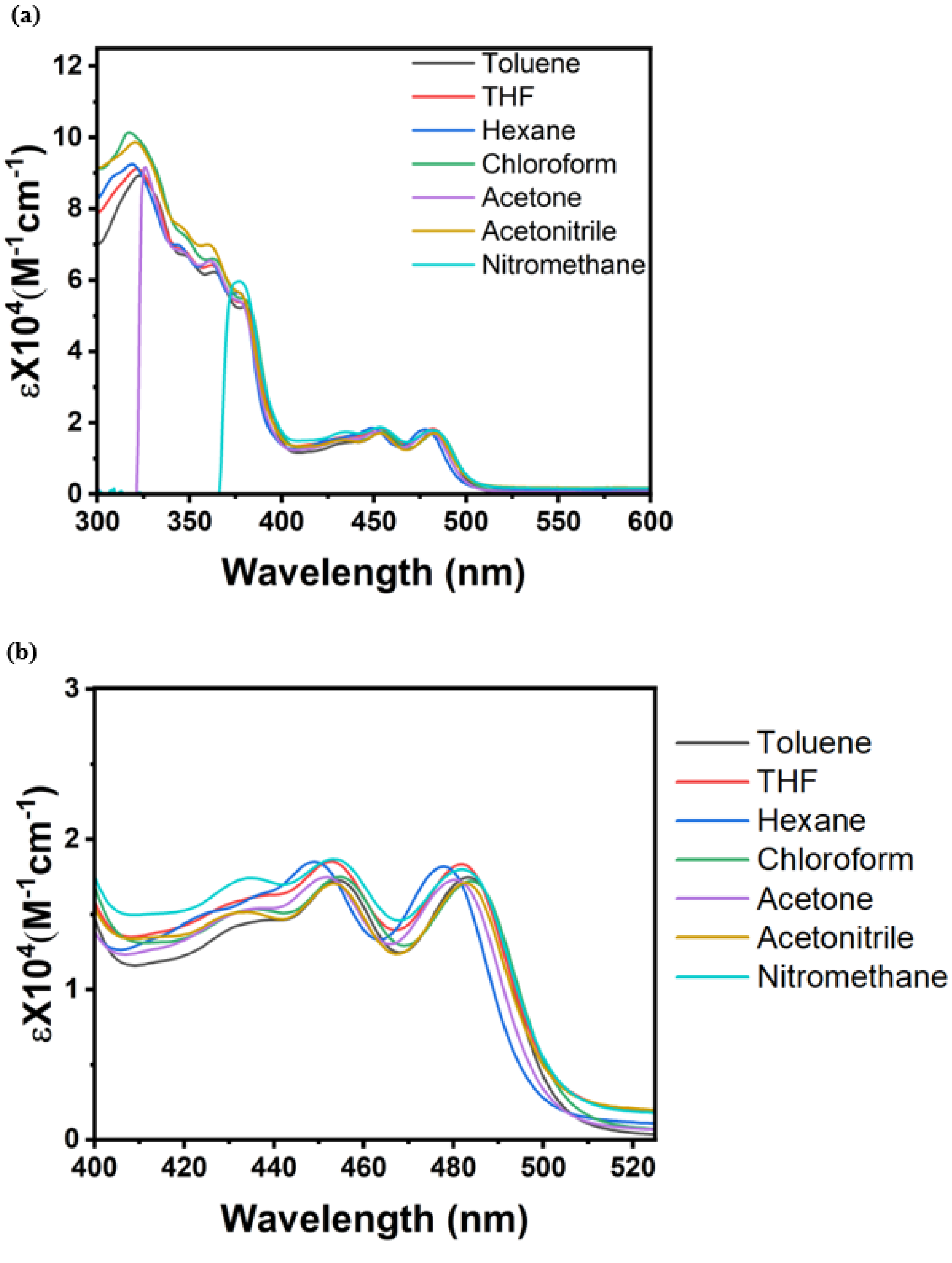

Figure S 53. UV-vis absorption spectrum of *PPP*-**HT₈H** dissolved in different solvents and measured at 298 K. (a) full UV-vis absorption spectrum in different solvents (b) magnification of the lowest energy transition of *PPP*-**HT₈H** in the different solvents.

S4.2 ECD spectra

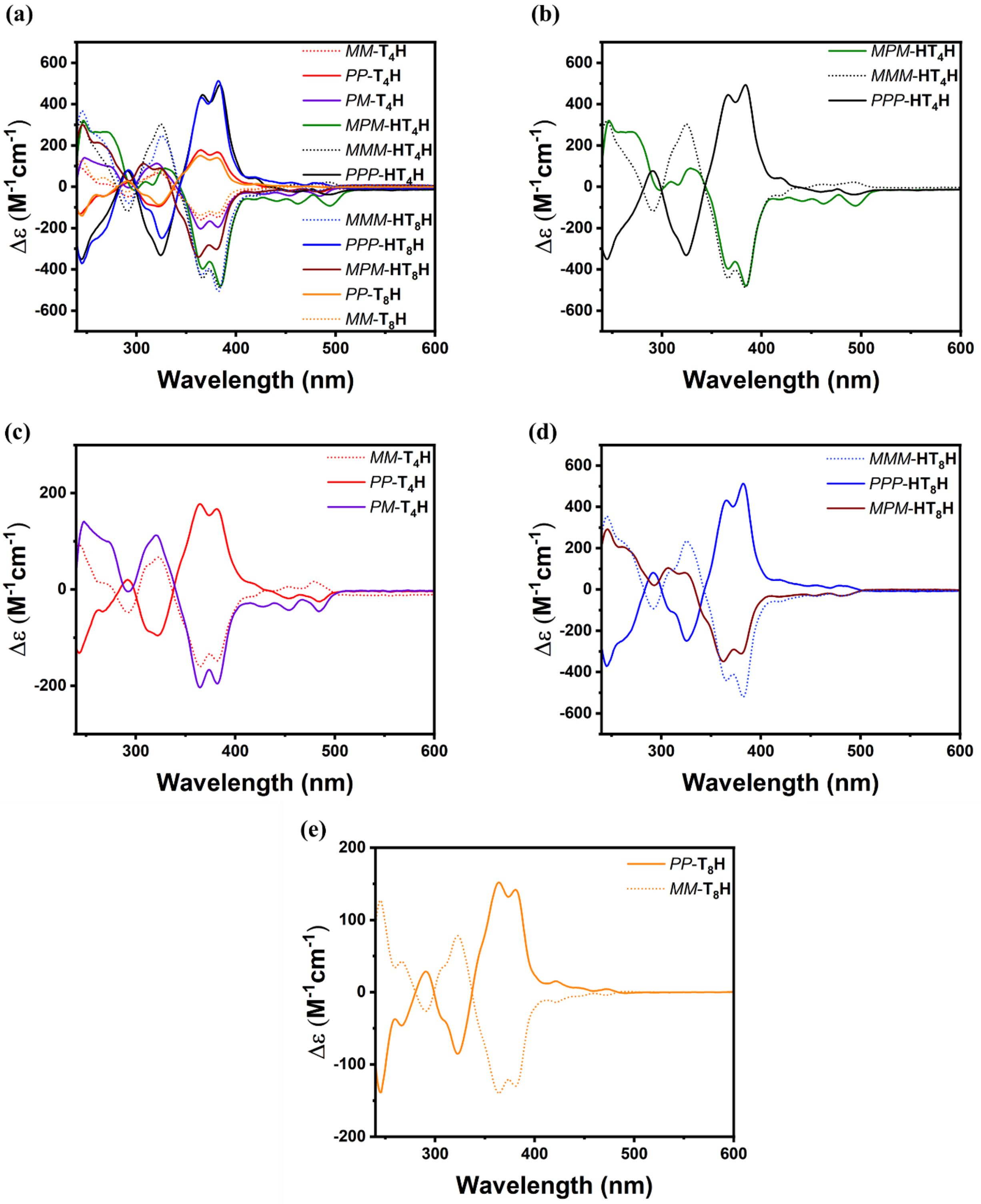

Figure S 54. ECD spectra of the compounds indicated on each subfigure dissolved in THF and measured at 298 K. (a) all synthesized helitwistacene compounds (b) **HT$_4$H** helitwistacene molecules (c) **T$_4$H** helitwistacene molecules (d) **HT$_8$H** helitwistacene molecules (e) **T$_8$H** helitwistacene molecules.

S4.2.1 ECD spectra of helitwistacene molecules in different solvents

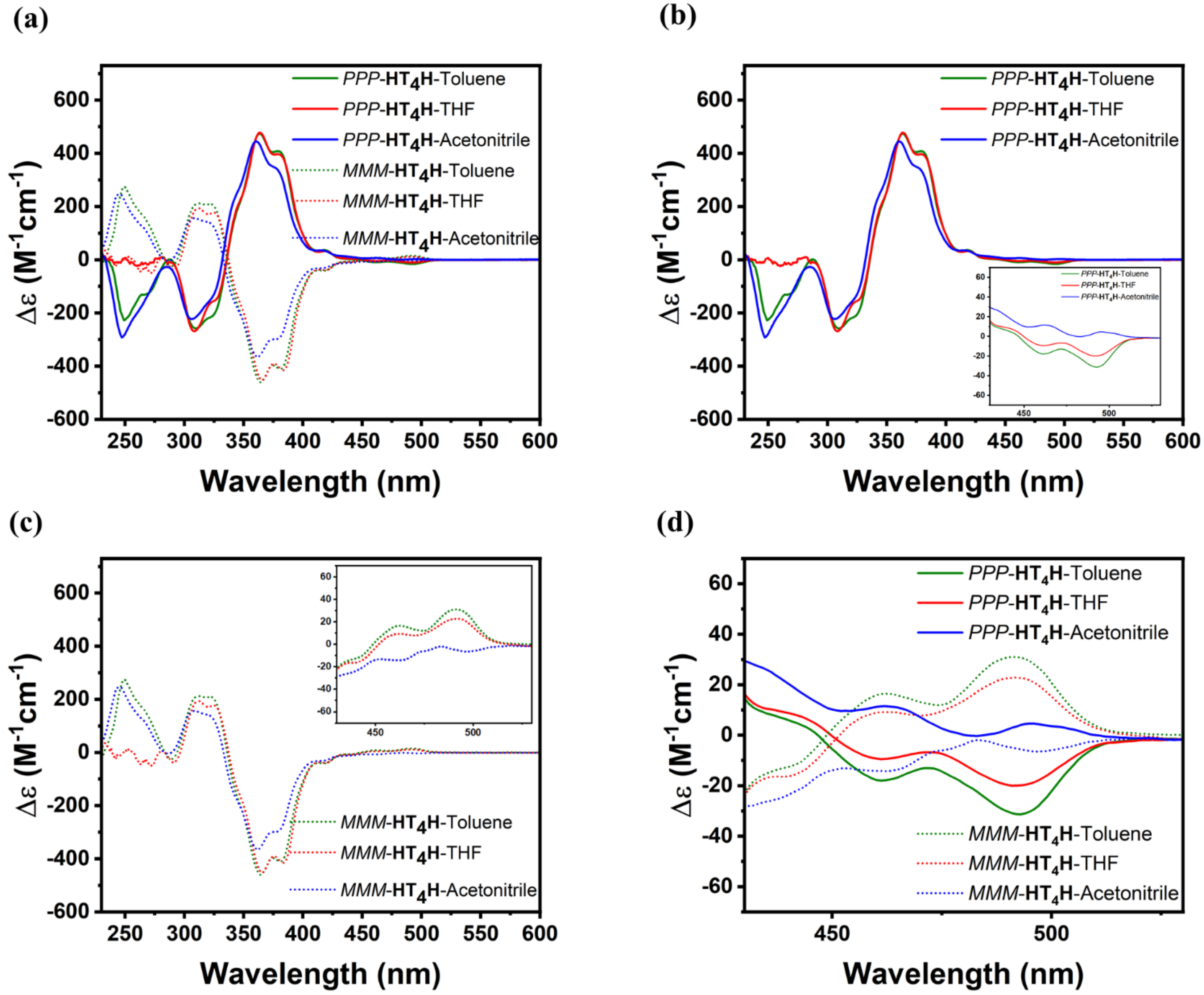

Figure S 55. ECD spectra measured at 298 K of: (a) enantiomers *PPP*-**HT$_4$H** and *MMM*-**HT$_4$H** in three solvents (in THF, acetonitrile and toluene) that have different polarities; (b) *PPP*-**HT$_4$H** (inset shows the lowest energy transition); (c) *MMM*-**HT$_4$H** (inset shows the lowest energy transition); (d) magnification of the lowest energy transition of both compounds in the three solvents.

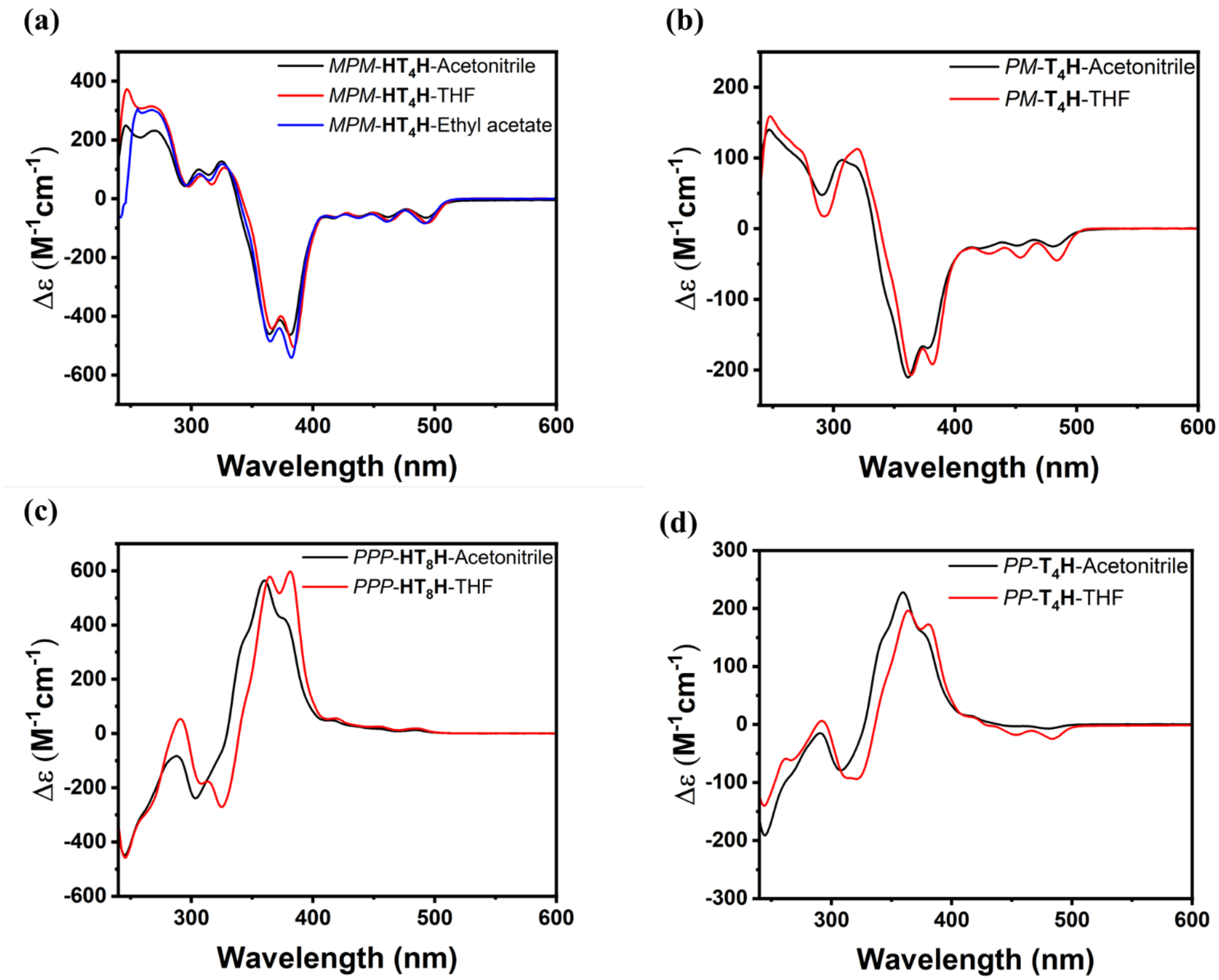

Figure S 56. CD spectra of (a) *MPM*-**HT$_4$H**; (b) *MP*- **T$_4$H**; (c) *PPP*-**HT$_8$H**; and (d) *PP*- **T$_4$H** in THF, acetonitrile and toluene measured at 298 K.

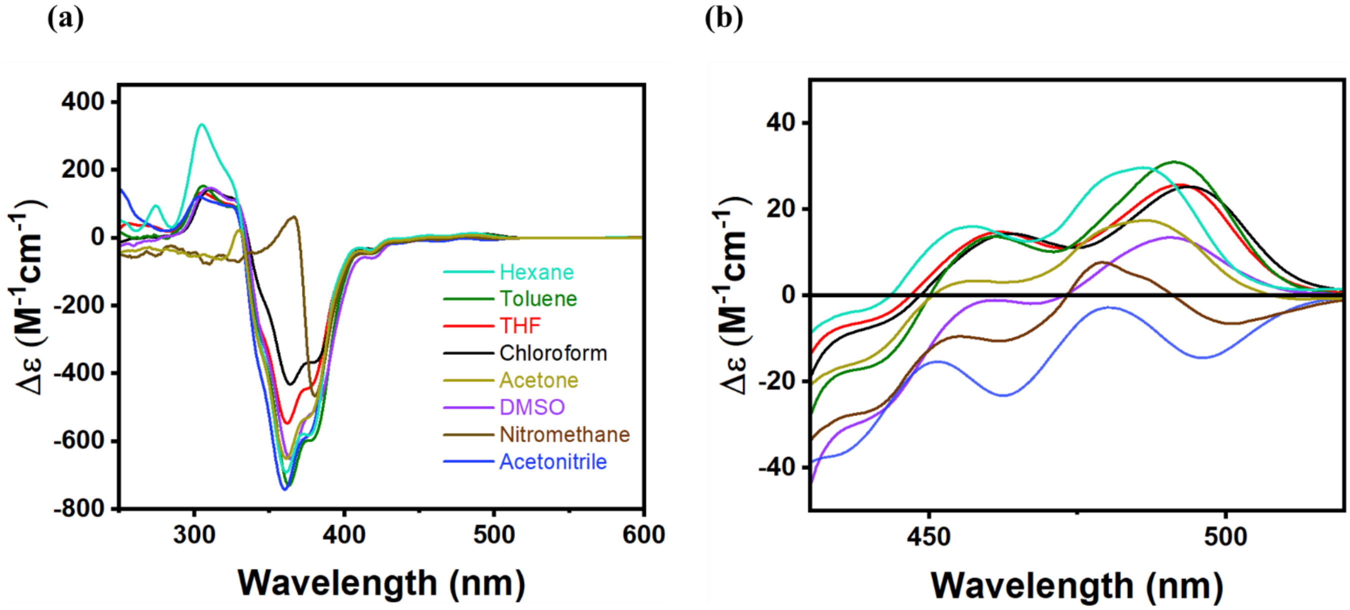

Figure S 57. ECD spectrum of *MMM*-**HT$_4$H** dissolved in different solvents and measured at 298 K. (a) full CD spectrum in different solvents (b) magnification of the lowest energy transition of *MMM*-HT$_4$H in the different solvents.

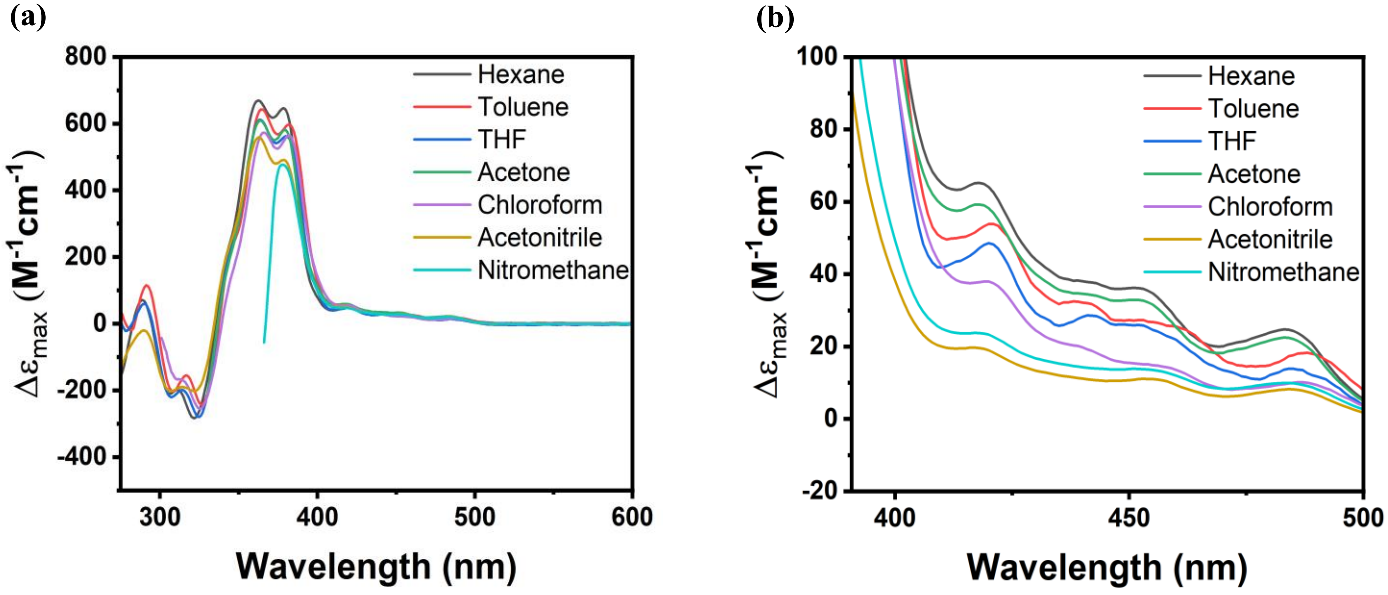

Figure S 58. ECD spectrum of *PPP*-**HT$_8$H** dissolved in different solvents and measured at 298 K. (a) full CD spectrum in different solvents (b) magnification of the lowest energy transition of *PPP*-**HT$_8$H** in the different solvents.

S4.2.2 The effect of solvents viscosity on the ECD.

In the Figure below, we show the analysis of the maximal ellipticity as a function of either solvent viscosity (left) and dipole moment (right). While there is no correlation between viscosity and $\Delta\varepsilon_{max}$, there is a clear trend when the dipole moment is considered. It is perhaps most obvious to observe a solvent pair such as hexane acetonitrile having similar viscosity (0.31 and 0.38 respectively), and very different dipole moments (0.08 and 3.44, respectively). Each of these solvents are in the most extreme positive or negative scales of the molar ellipticity values. It is therefore obvious that viscosity does not affect the chiroptical response in a noticeable manner.

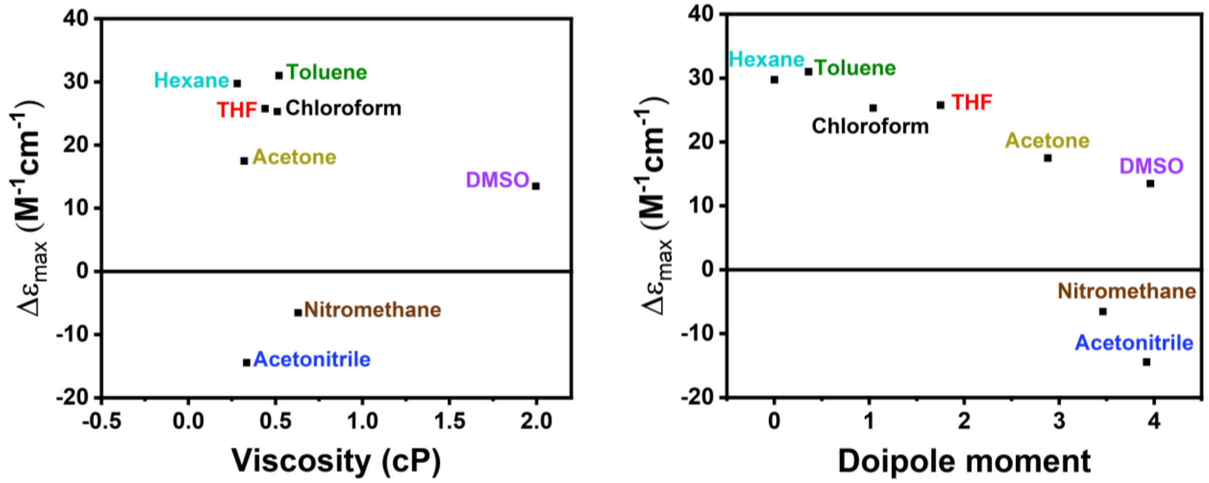

Figure S 59. The maximal ECD intensity of the S0→S1 transition vs. viscosity (left) and dipole moment (right) of *MMM*-**HT**$_4$**H**.

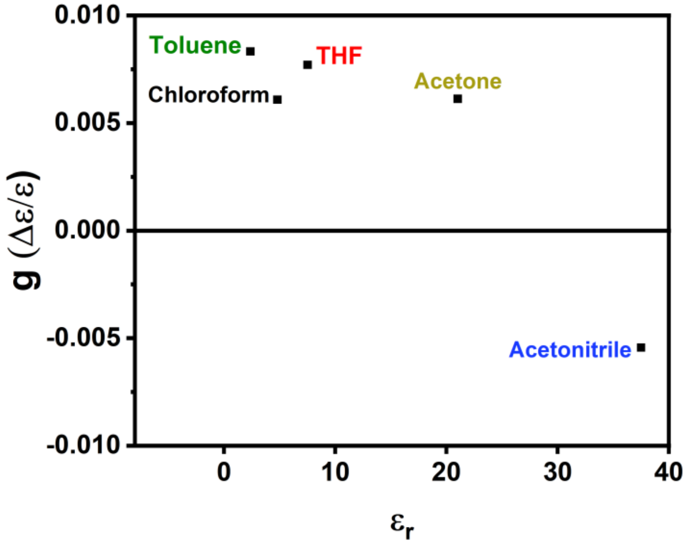

Figure S 60. The Anisotropy factor g_{abs} vs. solvent polarity (dielectric constant) of *MMM*-**HT**$_4$**H**.

S4.2.3 ECD spectra of ***PPP***- HT_4H molecules in different temperatures.

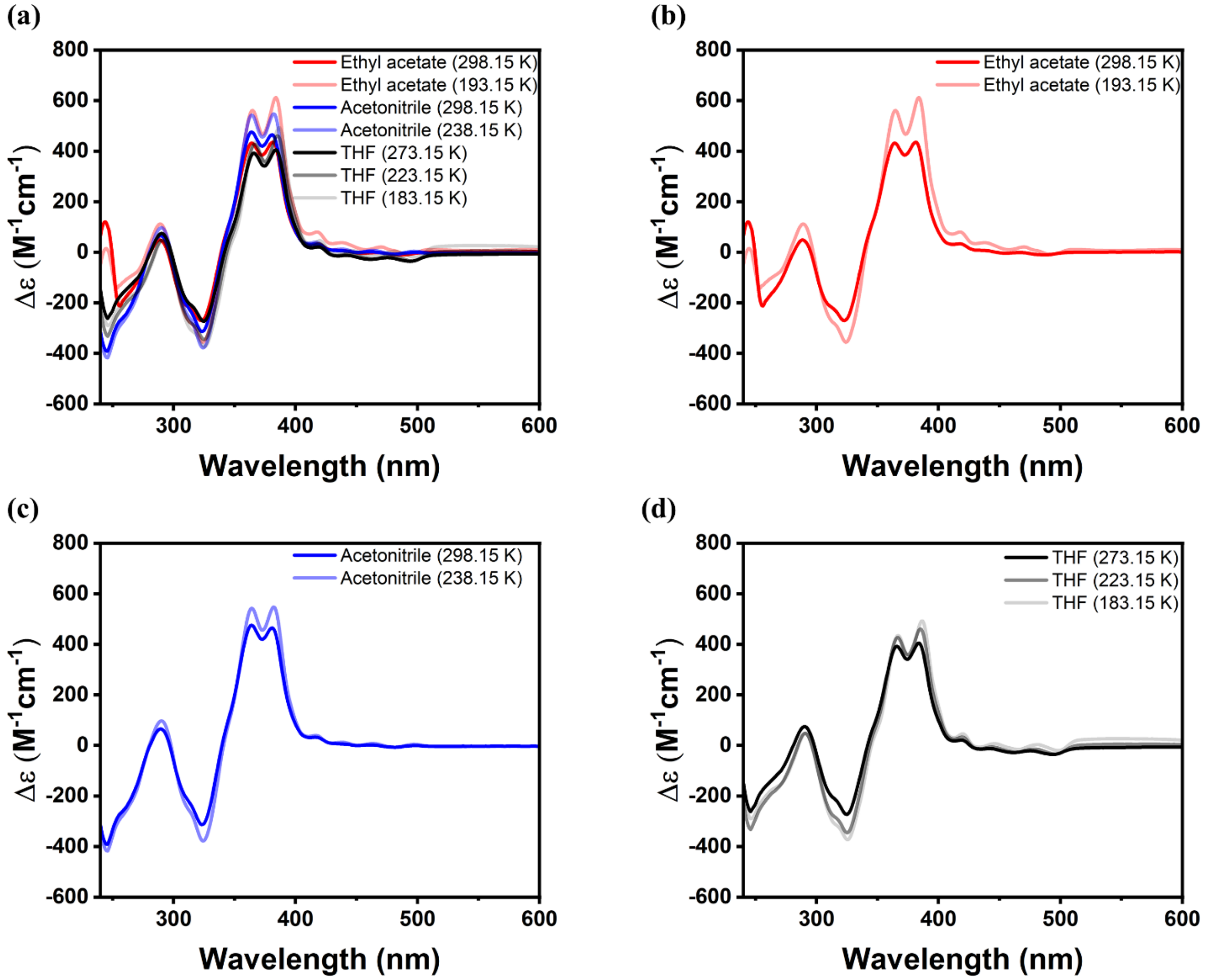

Figure S 61. CD spectra of *PPP*- $\mathbf{HT_4H}$ in different solvents and at different temperatures (a) all solvents and temperatures; (b) in ethyl acetate; (c) in acetonitrile; and (d) in THF.

S4.3 Steady-state fluorescence spectra

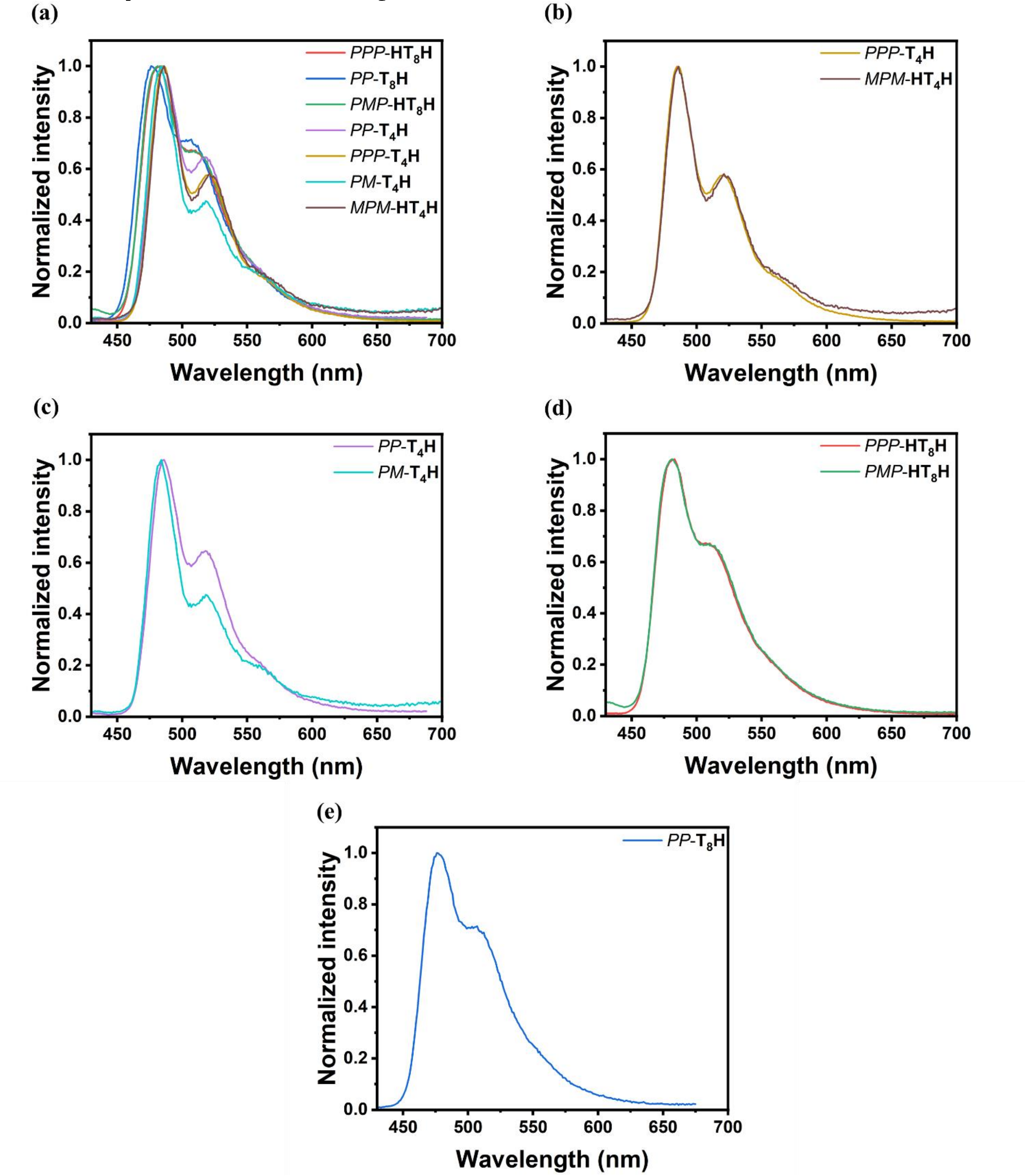

Figure S 62. Steady-state fluorescence spectra of the compounds indicated on each subfigure dissolved in THF and measured at 298 K. (a) all synthesized helitwistacene compounds (b) $\mathbf{HT_4H}$ helitwistacene molecules (c) $\mathbf{T_4H}$ helitwistacene molecules (d) $\mathbf{HT_8H}$ helitwistacene molecules (e) *PP*-$\mathbf{T_8H}$.

S4.3.1 Steady-state fluorescence spectra of *PPP*-**HT₄H** and *PPP*-**HT₈H** in different solvents

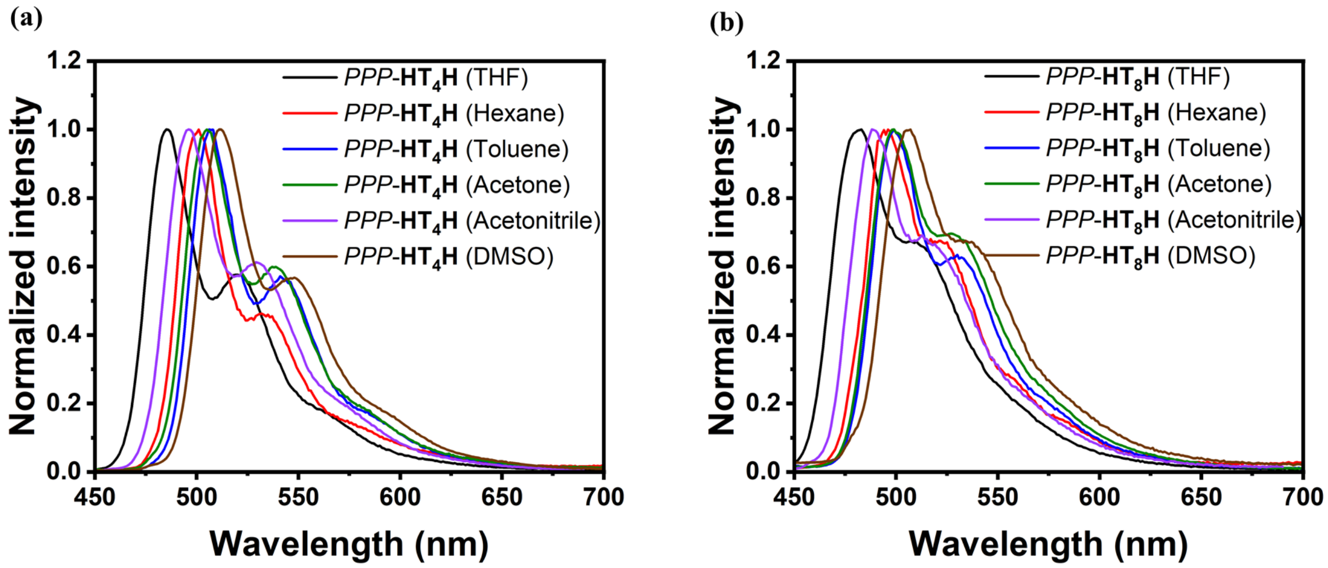

Figure S 63. Steady-state fluorescence spectra of the compounds (a) *PPP*-$\mathbf{HT_4H}$ (b) *PPP* -$\mathbf{HT_8H}$ dissolved in different solvents and measured at 298 K.

S4.3.2 Lippert-Mataga plot of (a) *PPP*-$\mathbf{HT_4H}$ and (b) *PPP*-$\mathbf{HT_8H}$.

Orientation polarizability **(Δ*f*)** values were calculated according to the Lippert-Mataga equation:

$$\Delta f = \frac{\mathcal{E} - 1}{(2\mathcal{E} + 1)} - \frac{n^2 - 1}{(2n^2 + 1)}$$

Where Ɛ is the dielectric constant, and ***n*** is the refractive index of the solvent.

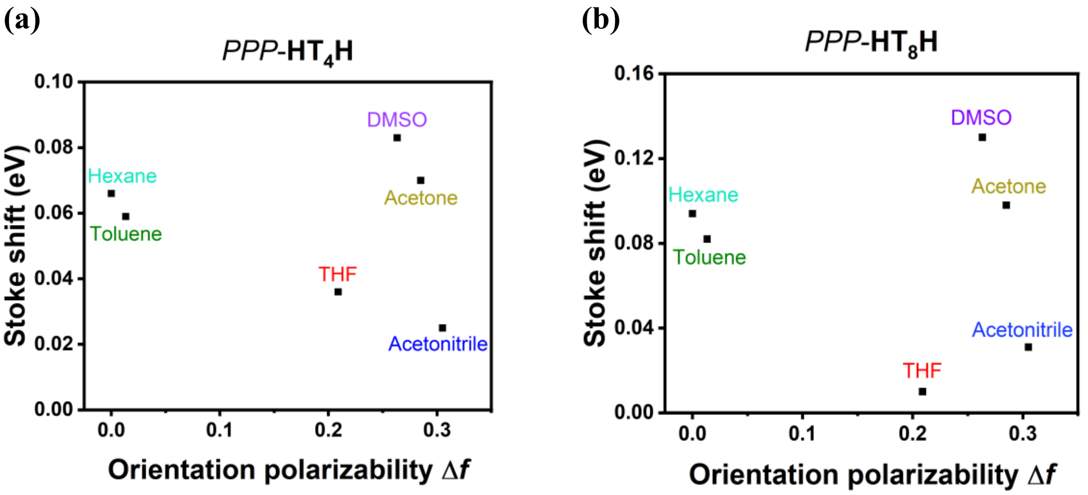

Figure S 64. Lippert-Mataga plot of (a) ***PPP*-HT$_4$H** and (b) ***PPP*-HT$_8$H**.

Stokes shift and Orientation polarizability (Δ*f*) calculations

Table 1. **Absorption (λ_{abs}) and emission (λ_{em}) maxima of *PPP*-**HT$_4$H**.**

Solvent	λ_{abs} (nm)	λ_{abs} (eV)	λ_{em} (nm)	λ_{em} (eV)	Stokes shift (eV)
**Hexane**	488	2.54	501	2.47	0.066
**Toluene**	494	2.51	506	2.45	0.059
**THF**	492	2.52	485	2.56	0.036
**Acetone**	491	2.52	505	2.45	0.070
**Acetonitrile**	491	2.52	496	2.50	0.025
**DMSO**	494	2.51	511	2.43	0.083

Table 2. Absorption (λ_{abs}) and emission (λ_{em}) maxima of *PPP*-**HT$_8$H**.

Solvent	λ_{abs} (nm)	λ_{abs} (eV)	λ_{em} (nm)	λ_{em} (eV)	Stokes shift (eV)
**Hexane**	478	2.59	496	2.50	0.094
**Toluene**	483	2.57	499	2.48	0.082
**THF**	481	2.58	483	2.57	0.010
**Acetone**	480	2.59	499	2.48	0.098
**Acetonitrile**	483	2.57	489	2.54	0.031
**DMSO**	485	2.56	511	2.43	0.13

Table 3. Orientation polarizability (Δf) information in different solvents.

**Solvent**	ε	***n***	***n²***	**Δ*f***
**Hexane**	1.89	1.3749	1.89035	4.59497E-5
**Toluene**	2.38	1.4969	2.24071	0.01324
**THF**	7.52	1.4072	1.98021	0.20888
**Acetone**	21.01	1.3586	1.84579	0.28485
**Acetonitrile**	36.64	1.3441	1.8066	0.30496
**DMSO**	47	1.4793	2.18833	0.26319

S4.4 Excitation spectra

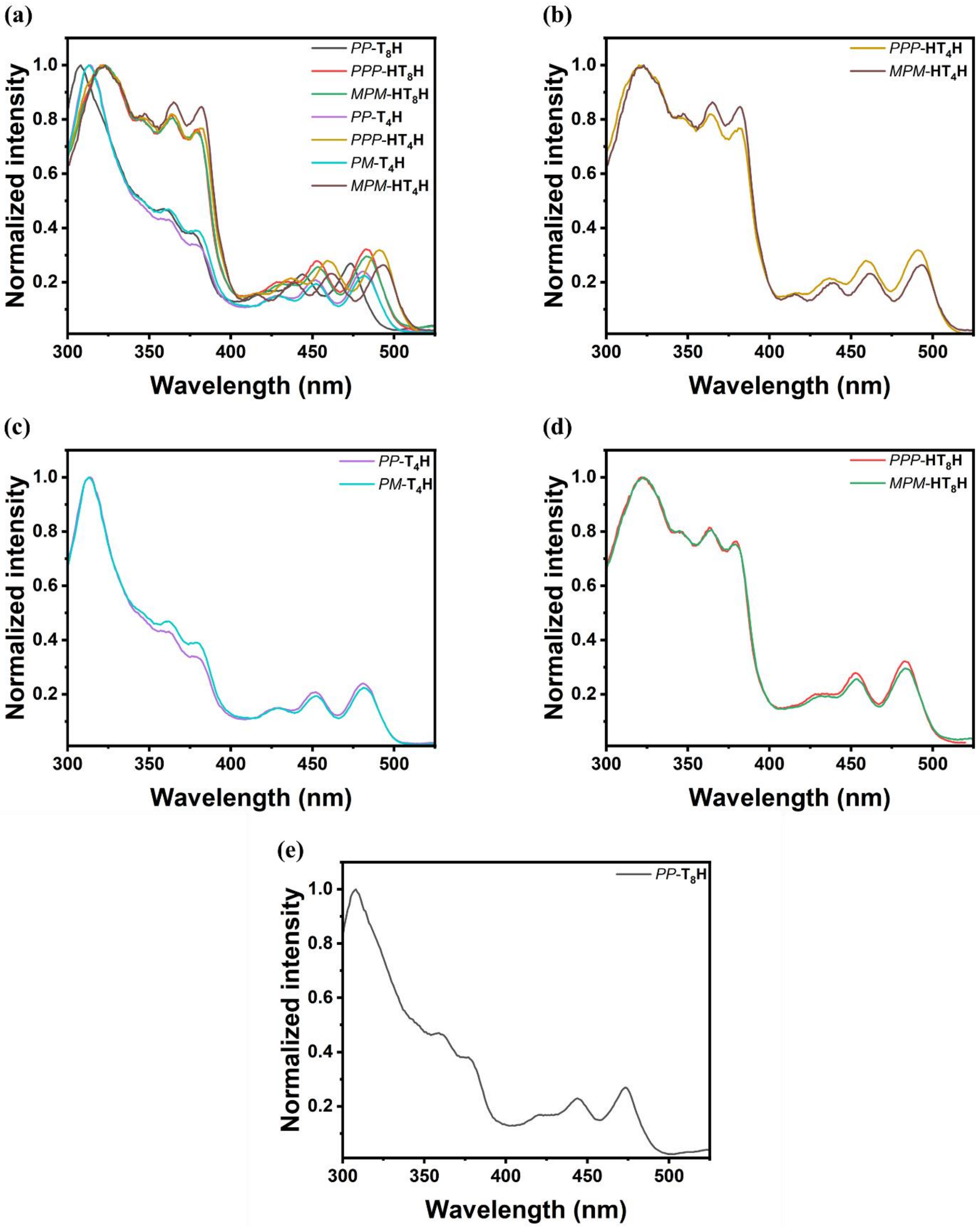

Figure S 65. Excitation spectra of the compounds indicated on each subfigure dissolved in THF and measured at 298 K. (a) all synthesized helitwistacene compounds (b) **HT$_4$H** helitwistacene molecules (c) **T$_4$H** helitwistacene molecules (d) **HT$_8$H** helitwistacene molecules (e) *PP*-**T$_8$H**.

S4.5 Circularly polarized luminescence (CPL).

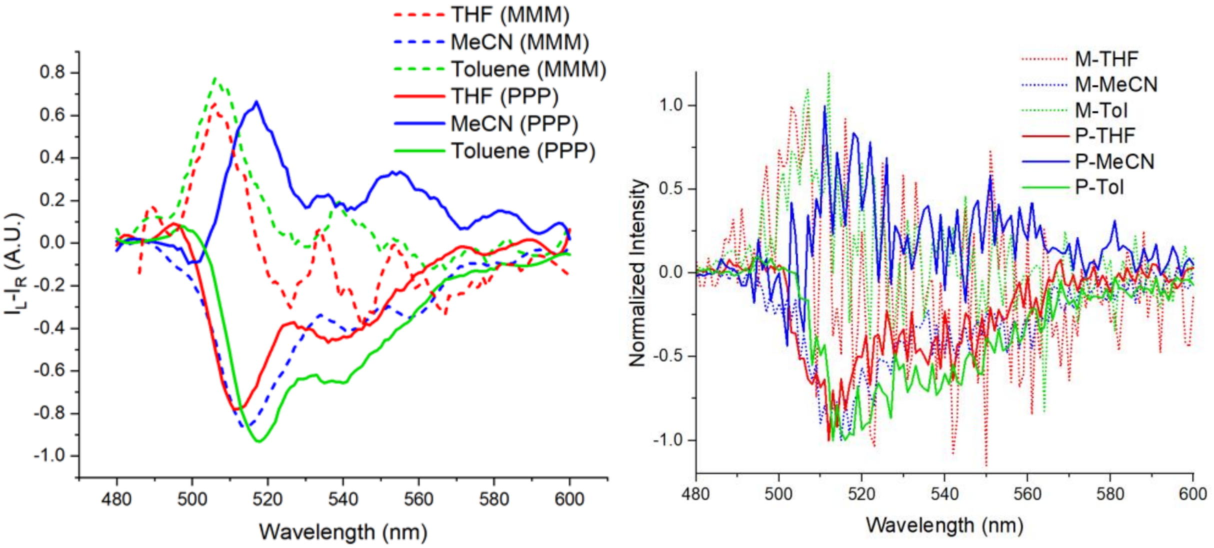

Figure S 66. Smoothed (left) and unsmoothed (right) CPL spectrum of *MMM*-**HT$_4$H** and *PPP*-**HT$_4$H** dissolved in THF, toluene and acetonitrile and measured at 298 K.

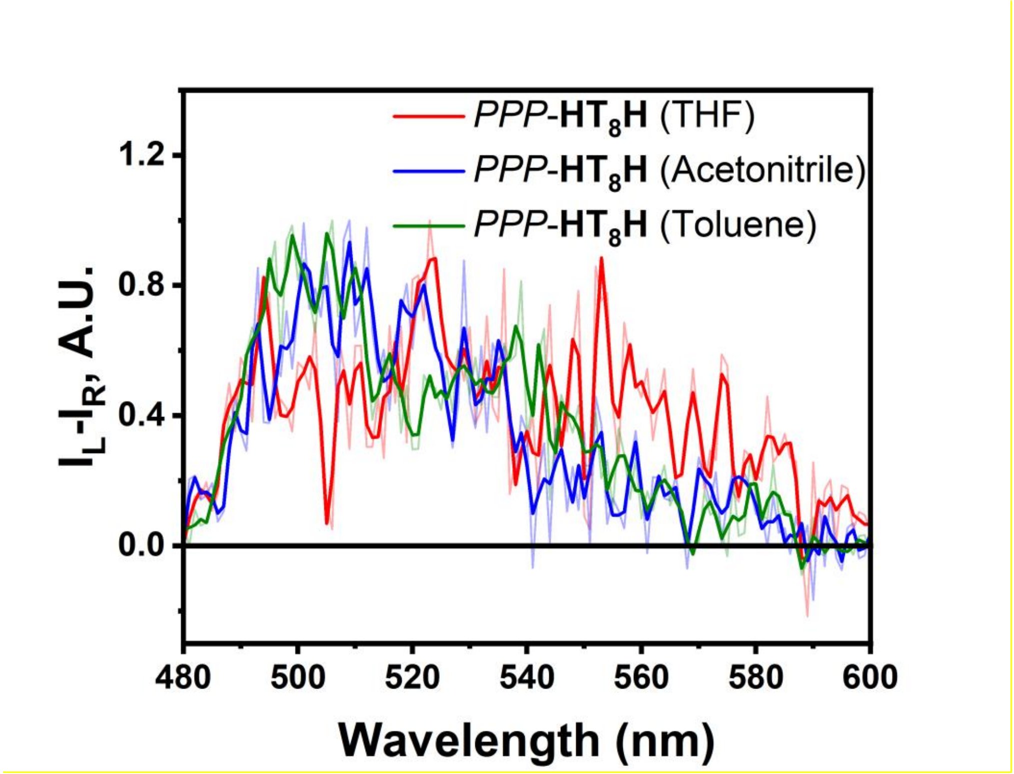

Figure S 67. Smoothed CPL spectrum of *PPP*-**HT$_8$H** dissolved in THF, toluene and acetonitrile and measured at 298 K.

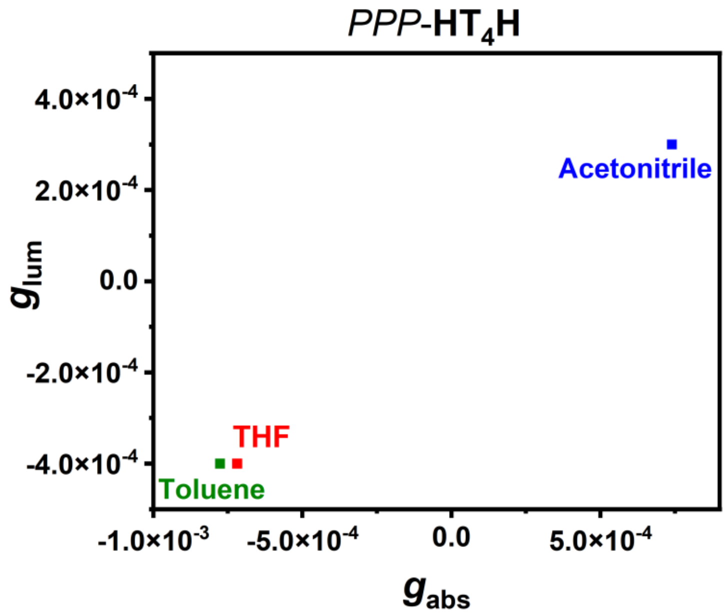

Figure S 68. Anisotropy factors g_{lum} versus g_{abs} of *PPP*-**HT$_4$H**.

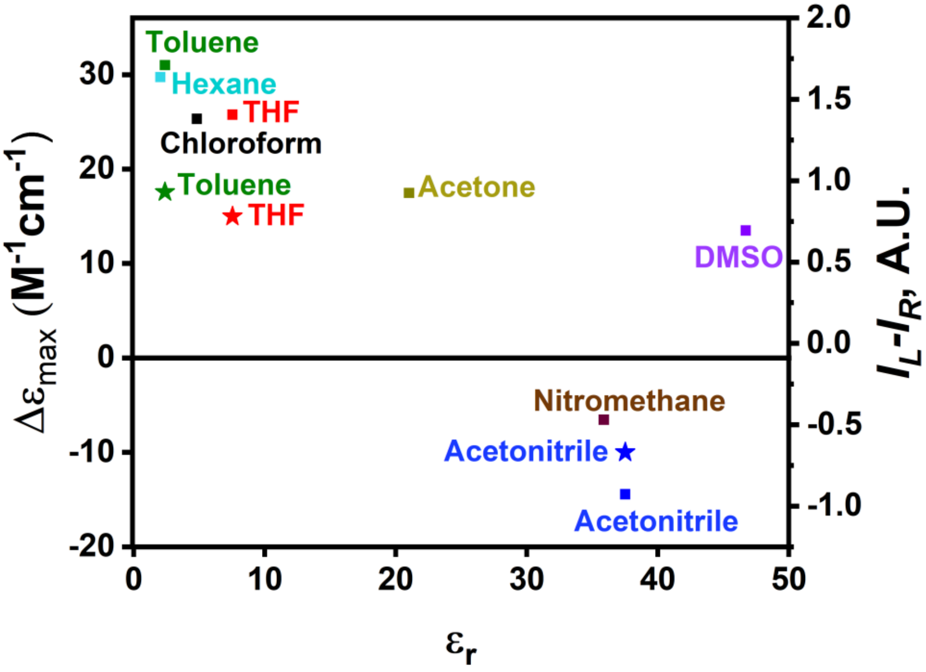

Figure S 69. The maximal ECD intensity of the $S_0 \rightarrow S_1$(square) and $S_1 \rightarrow S_0$ (star) transitions vs. solvent polarity (dielectric constant) of *MMM*-**HT$_4$H**.

S4.6 Quantum yields and CPL brightness factor of helitwistacenes molecules.

Table 4. Quantum yield of all synthesized helitwistacene compounds

	**Helitwistacene molecules**	**Quantum yield %**
1	*PPP*-**HT_4H**	51
2	*PP*-**T_4H**	51
3	*MPM*-**HT_4H**	38
4	*PM*-**T_4H**	49
5	*PPP*-**HT_8H**	44
6	*PP*-**T_8H**	50
7	*MPM*-**HT_8H**	53

Table 5. Quantum yield of ***PPP***-HT_4H and ***PPP***-HT_8H in different solvents

**Solvent**	Quantum yield of *PPP*-**HT_4H** (%)	Quantum yield of *PPP*-**HT_8H** (%)
**Toluene**	50	42
**THF**	51	44
**Acetonitrile**	38	35

Table 6. CPL brightness factors of *PPP*-**HT_4H** and *PPP*-**HT_8H** in THF

**Molecule**	ε (M^{-1} cm^{-1}) [λ_{max}] (nm)	**Quantum yield (Θ)**	**B= ε x Θ**	**$\lvert g_{lum}\rvert$**	$B_{CPL}= \frac{B \times \lvert glum\rvert}{2}$ ($M^{-1}cm^{-1}$)
PPP-**HT_4H**	19548 (492)	51%	9969.5	4 x10^{-4}	2
PPP-**HT_8H**	18780 (484)	44%	8263.2	5 x10^{-4}	2.06

S4.7 Fluorescence lifetime (τ_f)

Table 7. Fluorescence lifetime (τ_f) of all synthesized helitwistacene compounds

	Helitwistacene compounds	Lifetime (ns)
1	*PPP*-**HT$_4$H**	3.06
2	*PP*-**T$_4$H**	3.53
3	*MPM*-**HT$_4$H**	3.59
4	*PM*-**T$_4$H**	3.81
5	*PPP*-**HT$_8$H**	3.57
6	*PP*-**T$_8$H**	3.72
7	*MPM*-**HT$_8$H**	3.83

Table 8. Fluorescence lifetime (τ_f) of *PPP*-**HT$_4$H** and *PPP*-**HT$_8$H** in different solvents

Solvent	Lifetime of *PPP*-**HT$_4$H** (ns)	Lifetime of *PPP*-**HT$_8$H** (ns)
Toluene	2.98	3.45
THF	3.06	3.57
Acetonitrile	3.26	3.96

S5 Single crystal X-ray diffraction crystallography (SCXRD)

Single crystals of *MPM*-**HT$_4$H** and *PM*-**T$_4$H** were obtained from a mixture of DCM/hexane by slow evaporation.

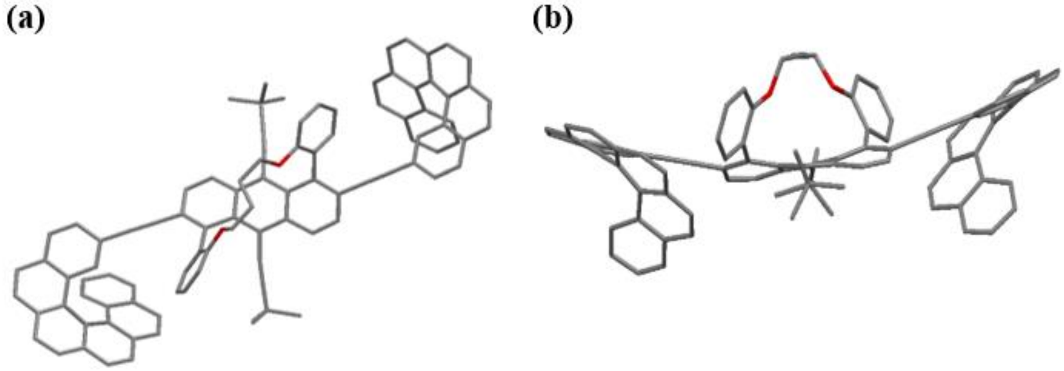

Figure S 70. Crystal structure of *MPM*-**HT₄H**; (a) top view. (b) side view.

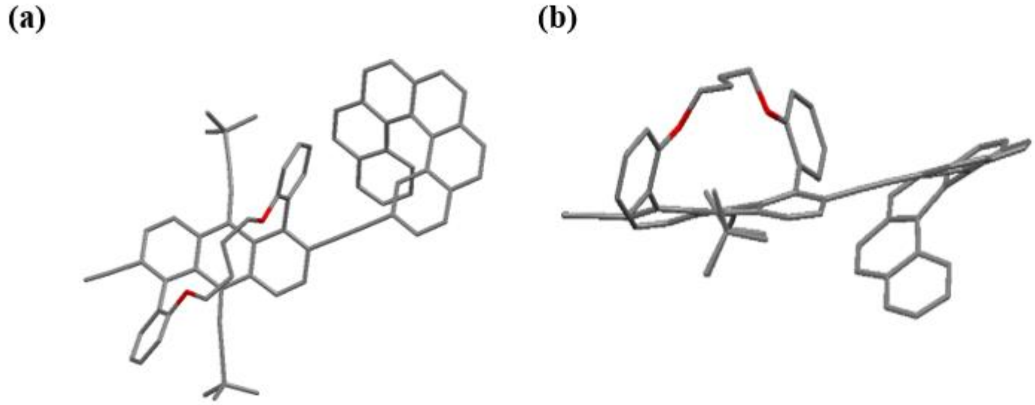

Figure S 71. Crystal structure of *PM*-**T₄H**; (a) top view; (b) side view.

Table 9. Crystal data and structure refinement for *MPM*-**HT₄H**.

**Parameter**	*MPM*-**HT$_4$H**
Empirical formula	$C_{98}H_{68}O_2$
CCDC No.	2292979

Formula weight	1277.52
Temperature/K	100.0(1)
Crystal system	orthorhombic
Space group	$P2_12_12$
a/Å	27.351(1)
b/Å	16.3358(6)
c/Å	7.8161(2)
α/°	90
β/°	90
γ/°	90
Volume/Å^3	3492.2(2)
Z	2
ρ_{calc}g/cm^3	1.215
μ/mm^{-1}	0.071
F(000)	1344.0
Crystal size/mm^3	0.1x0.1x0.6
Radiation	Mo Kα (λ = 0.71073)
2Θ range for data collection/°	5.116 to 57.956
Index ranges	$-37 \leq h \leq 36$, $-21 \leq k \leq 17$, $-9 \leq l \leq 10$
Reflections collected	23641
Independent reflections	8818 [R_{int} = 0.0323, R_{sigma} = 0.0461]
Data/restraints/parameters	8818/0/454
Goodness-of-fit on F^2	1.036
Final R indexes [I>=2σ (I)]	R_1 = 0.0734, wR_2 = 0.1838
Final R indexes [all data]	R_1 = 0.0971, wR_2 = 0.1956
Largest diff. peak/hole / e Å^{-3}	0.41/-0.23
Flack parameter	0.1(8)

Table 10. Crystal data and structure refinement for *PM*-**T$_4$H**.

**Parameter**	*PM*- **T$_4$H**
Empirical formula	$C_{72}H_{54}O_2$
CCDC No.	2292980

Formula weight	951.15
Crystal system	orthorhombic
Space group	$P\ 2_12_12_1$
Crystal size	0.144 x0.035 x0.030
Crystal color and shape	Yellow prism
Temperature (K)	100
wavelength (Å)	1.54178
a, (Å)	7.6356(1)
b, (Å)	16.6785(2)
c, (Å)	40.5077(3)
α, (°)	90
β, (°)	90
γ, (°)	90
Volume ($Å^3$)	5158.6(1)
Z	4
$\rho_{calcd,}$(g cm^{-1})	1.225
μ, mm^{-1}	0.552
No. of reflection (unique)	97131(9698)
R_{int}	0.0350
Completeness to θ (%)	94.6
data / restraints / parameters	9698/48/673
goodness-of-fit on F^2	1.056
Final R_1 and wR_2 indices [$I > 2\sigma(I)$]	0.0461,0.1249
R_1 and wR_2 indices (all data)	0.0521,0.1290

S6 Computational details

All calculations were carried out using the Gaussian 09 program applying density functional theory (DFT).[12] All molecules were optimized using a hybrid density functional and Becke's three parameter exchange functional combined with the LYP correlation functional (B3LYP) and with the 6-31G(d) basis set (B3LYP/6-31G(d)).[13] To understand the UV-vis and CD spectral transition

of the molecules, time dependent (TD)-DFT calculations were performed using the CAM-B3LYP functional with the 6-31G(d) basis set. Scanning potential energy surface was performed using DFT/B3LYP-GD3/6-31G(d) (using Grimmes's dispersion with the D3 damping function). [14]

S6.1 Calculated structures of the synthesized molecules

Table 11. Optimized (DFT-B3LYP-6-31G(d)) structures of the synthesized molecules

Molecule	Absolute energy (Hartree)
MP-**T$_4$H**	-2926.459961
PP-**T$_4$H**	-2926.457851
MPM-**HT$_4$H**	-3925.952596
PPP-**HT$_4$H**	-3925.95712
PPP-**HT$_8$H**	-4083.227246
MPM-**HT$_8$H**	-4083.224448
PP-**T$_8$H**	-2926.633614

Optimized (DFT-B3LYP-6-31G(d)) geometries of *PM*-**T$_4$H** and *PP*-**T$_4$H**

PM-**T$_4$H**

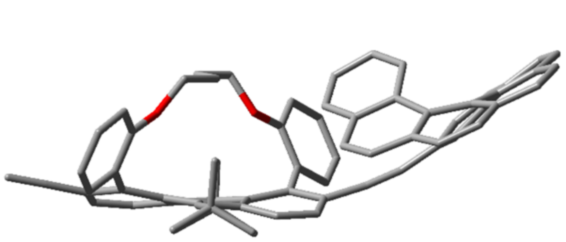

PP-**T$_4$H**

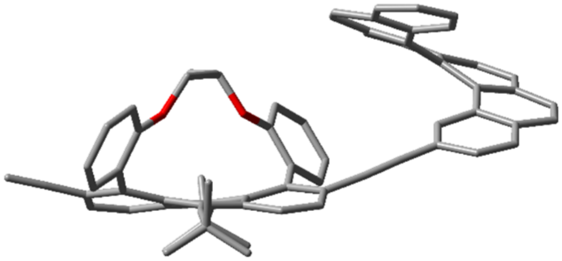

Optimized (DFT-B3LYP-6-31G(d)) geometries of *MPM*-**HT$_4$H** and *PPP*-**HT$_4$H**

MPM-$\mathbf{HT_4H}$

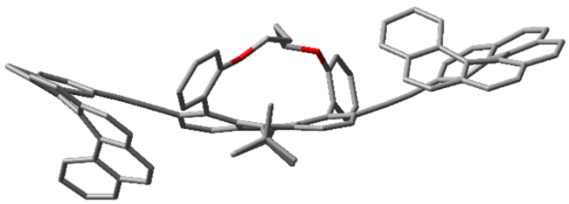

PPP-$\mathbf{HT_4H}$

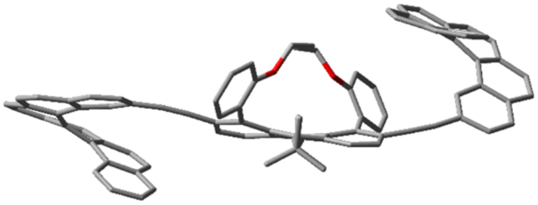

Optimized (DFT-B3LYP-6-31G(d)) geometries of ***MPM*- $\mathbf{HT_8H}$** and ***PPP*- $\mathbf{HT_8H}$**

MPM-$\mathbf{HT_8H}$

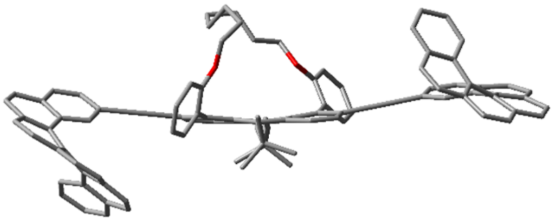

PPP-$\mathbf{HT_8H}$

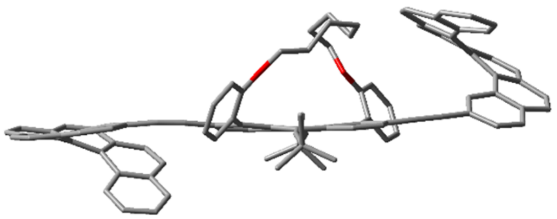

Optimized (DFT-B3LYP-6-31G(d)) geometries of *PP*- $\mathbf{T_8H}$

PP- $\mathbf{T_8H}$

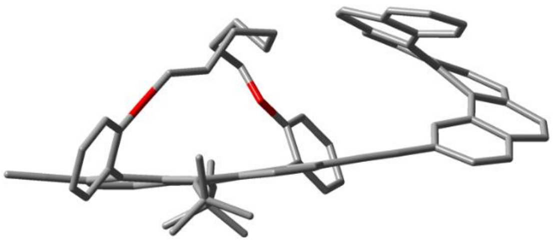

S6.2 Calculated UV-vis absorption spectrum and CD spectrum of the synthesized molecules

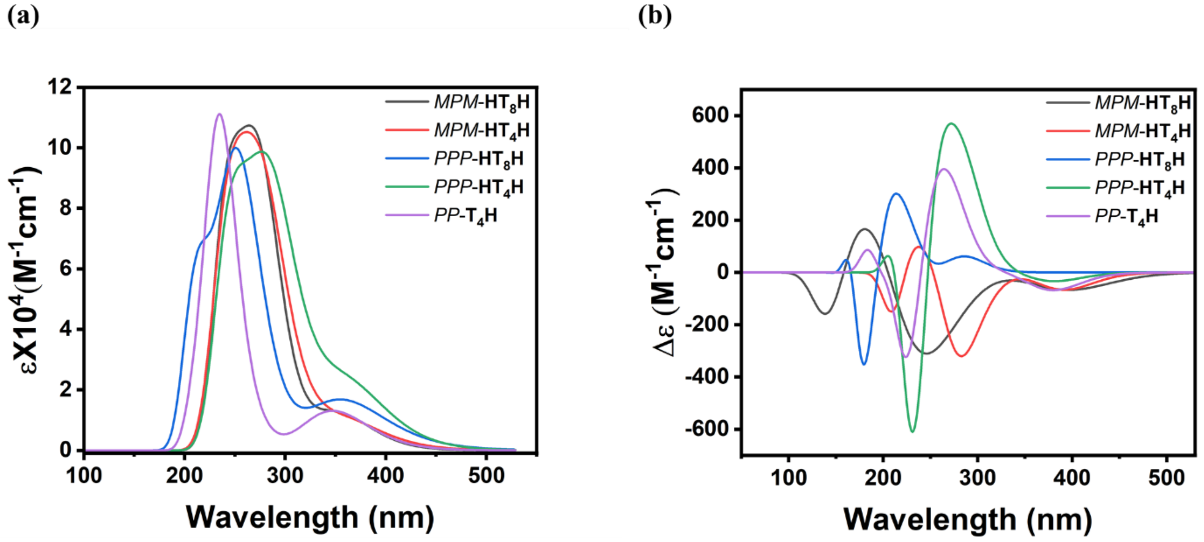

Figure S 72. Calculated (TD-DFT-6-31G(d)-CAMB3LYP) spectra of *MPM*-**HT_8H**, *MPM*-**HT_4H**, *PPP*-**HT_8H**, *PPP*-**HT_4H**, *PP*-**T_4H**: (a). UV-vis absorption spectrum, (b). ECD spectrum.

S6.3 Scanning potential energy surface

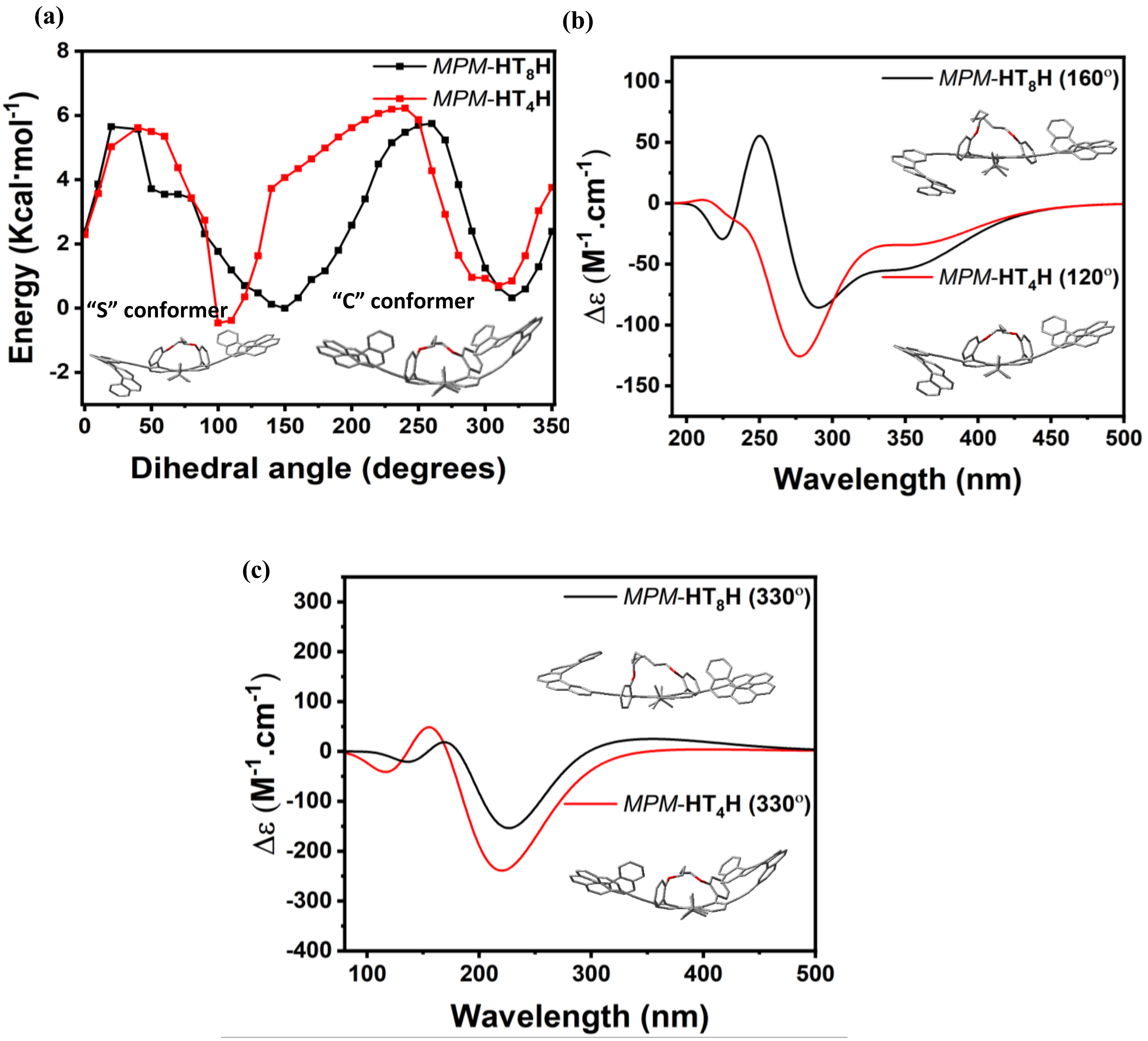

Figure S 73. (a) Calculated (B3LYP-GD3/6-31G(d)) torsional energy of *MPM*-**HT$_4$H** (red) and *MPM*-**HT$_8$H** (black). Calculated (TD-DFT/CAM-B3LYP/6-31G(d)) ECD for *MPM*-**HT$_4$H** and *MPM*-**HT$_8$H** (b) at their global minima (c) local minima.

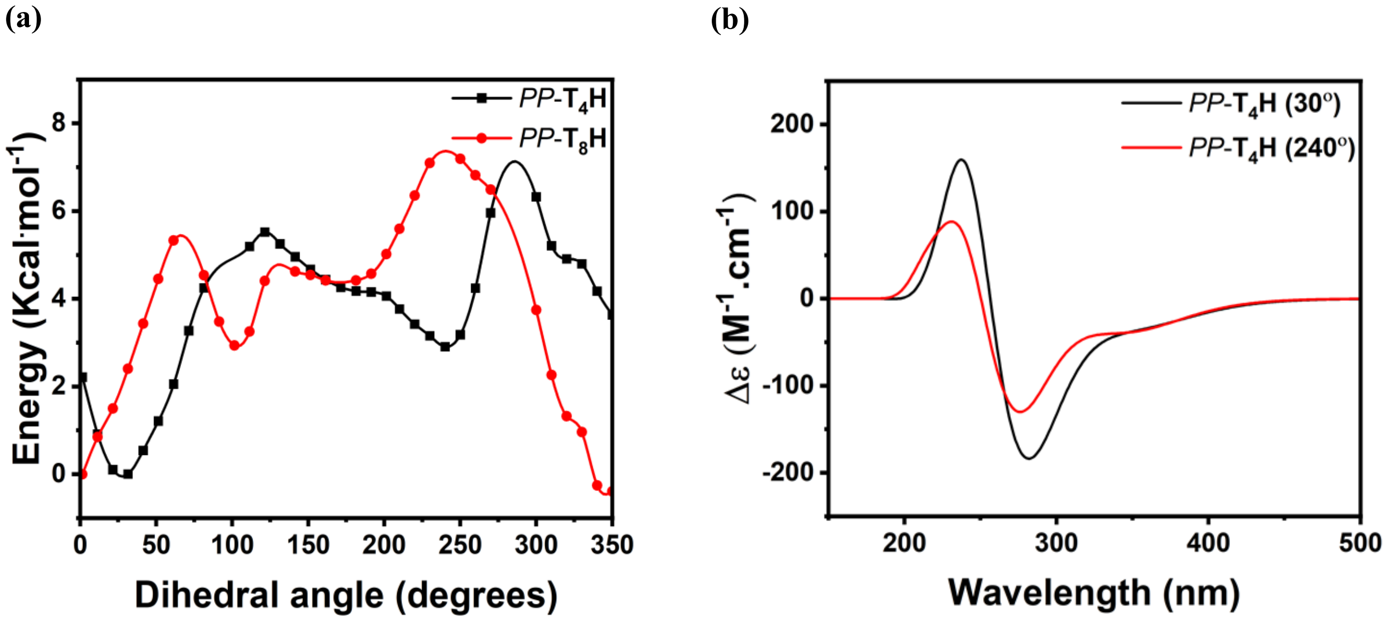

Figure S 74. (a) Calculated (B3LYP-GD3/6-31G(d)) torsional energy of *PP*-**T_4H** (black) and *PP*-**T_8H** (red). (b) Calculated (TD-DFT/CAM-B3LYP/6-31G(d)) ECD for *PP*-T_4H at their global minima and local minima.

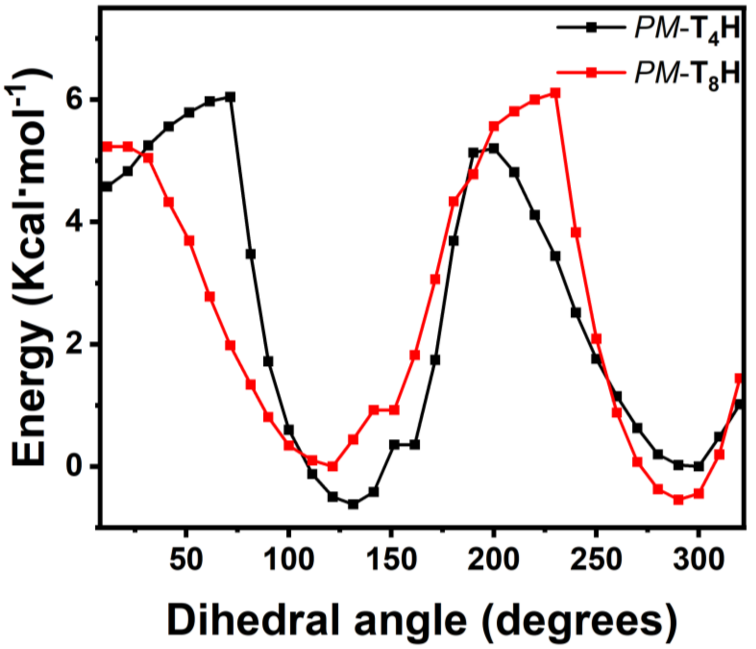

Figure S 75. Calculated (B3LYP-GD3/6-31G(d)) torsional energy of *PM*-**T_4H** (black) and *PM*-**T_8H** (red).

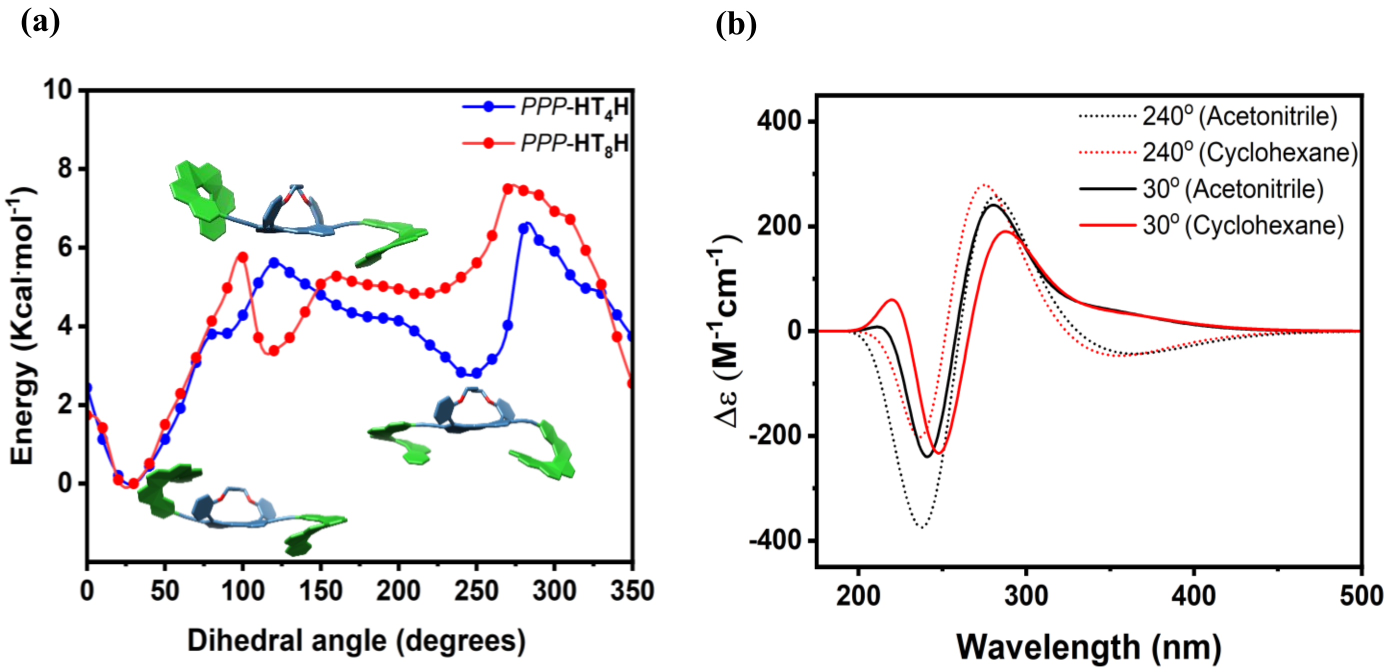

Figure S 76. (a) Calculated (B3LYP-GD3/6-31G(d)) torsional energy of *PPP*-**HT**$_4$**H** (blue) and *PPP*-**HT**$_8$**H** (red). (b) Calculated ECD spectra (TD-DFT/CAM-B3LYP/6-31G(d)) using both the GD3 dispersion correction and the CPCM model for *PPP*-**HT**$_4$**H** at its local and global minima.

S7 NMR analysis in different solvents

S7.1 NMR analysis of *PPP*-**HT$_4$H** in different solvents

To study the effect of solvents on the conformation of helitwistacenes, we have recorded the NMR spectra of *PPP*-**HT$_4$H** in different ratios of $CDCl_3$:CD_3CN. As can be observed in Figure S51, the most significant changes in the chemical shift are related to protons in the vicinity of the rotatable bond (1’, 3’, 4’) or in the vicinity of the bridge (13’).

For example: proton 13’, which should be affected by the free rotation with closer interaction to the tether, is shifted downfield by 0.12 ppm, while protons 16’ or 15’ which should be significantly less affected, is shifted by 0.05 and 0.02 ppm, respectively. We note that since the helicene moiety consist of only C-H bonds, it is relatively apolar and therefore the chemical shift should not be significantly affected by the difference in the dielectric constant.

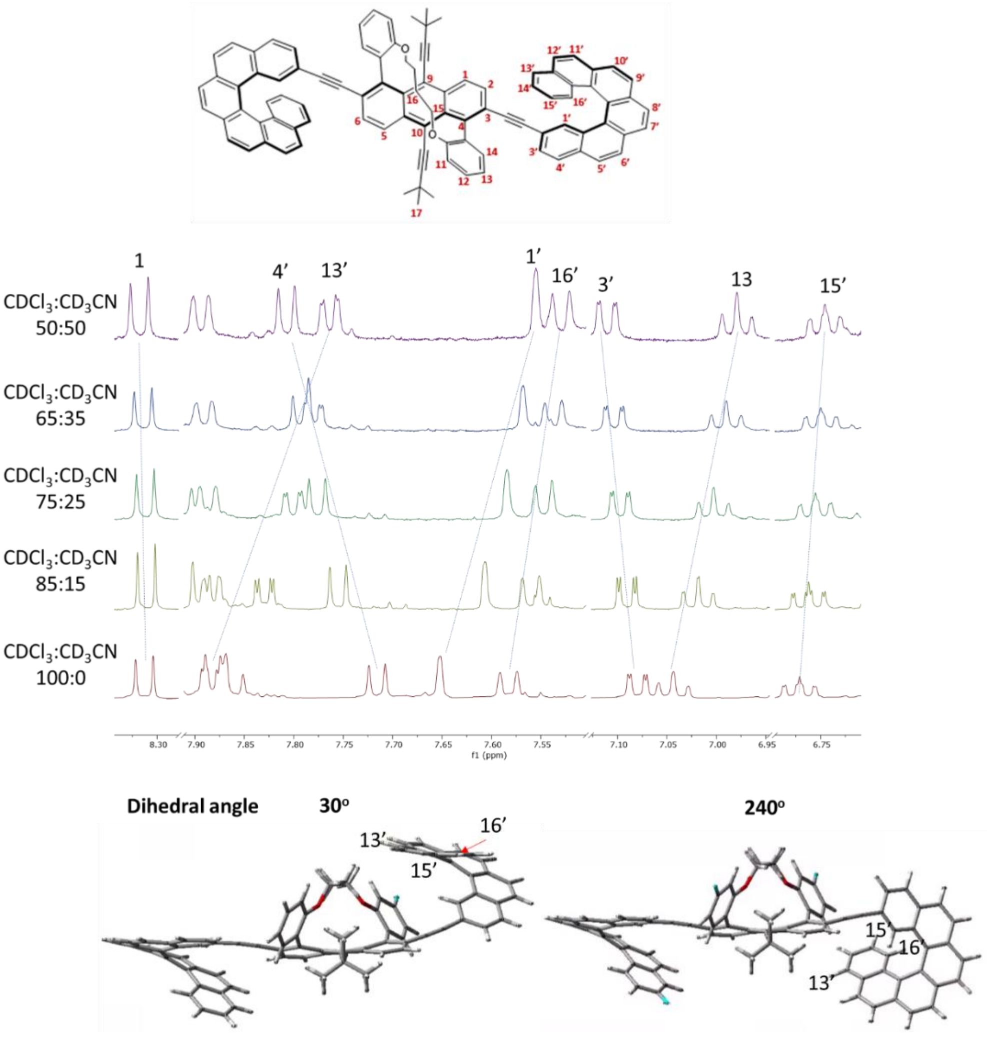

Figure S 77. NMR spectra of *PPP*-**HT$_4$H**. For Full spectra and analysis, see Figure S1. The proximity of 13’ to the bridge in the 30° conformation is depicted in the structure below the spectra.

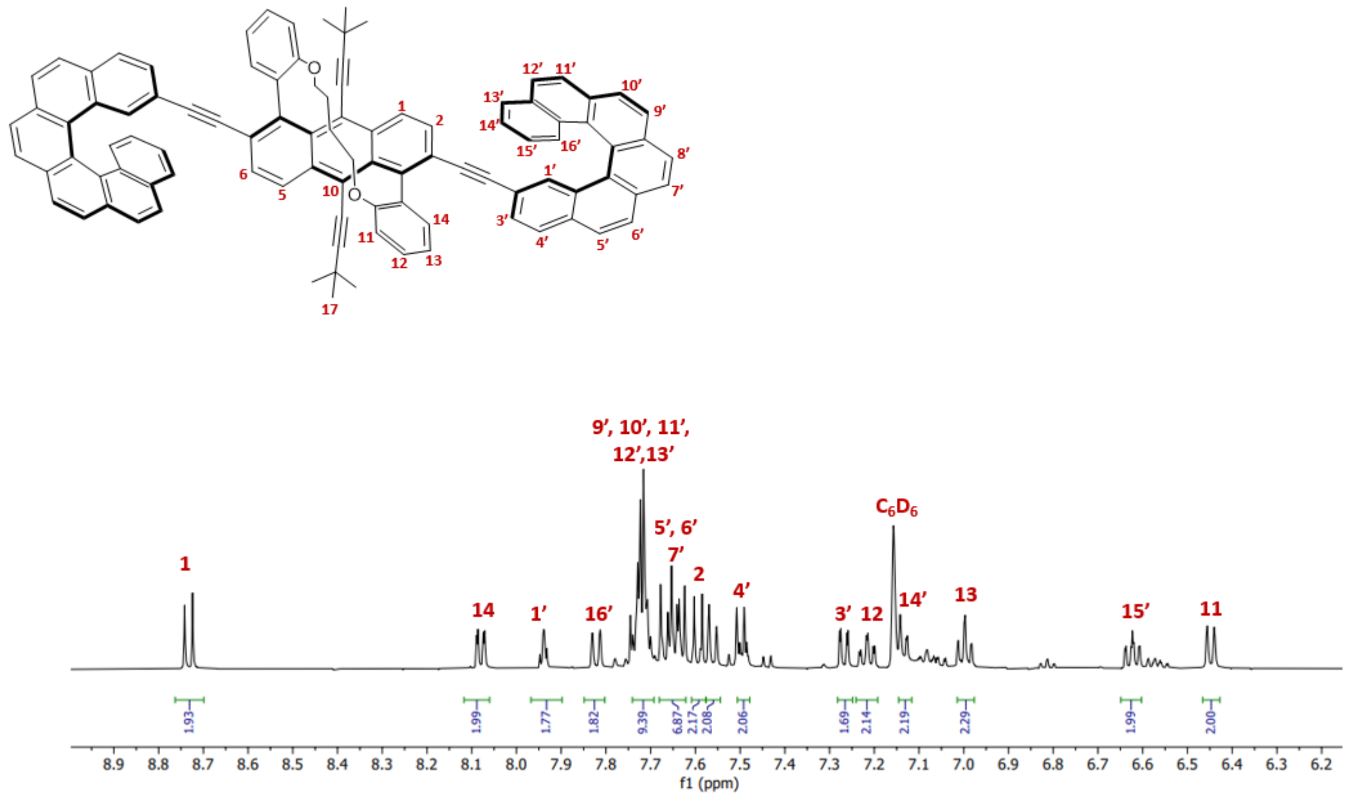

Figure S 78. ^{1}H NMR (500 MHz) of *PPP*-**HT₄H** in C_6D_6, measured at 298 K.

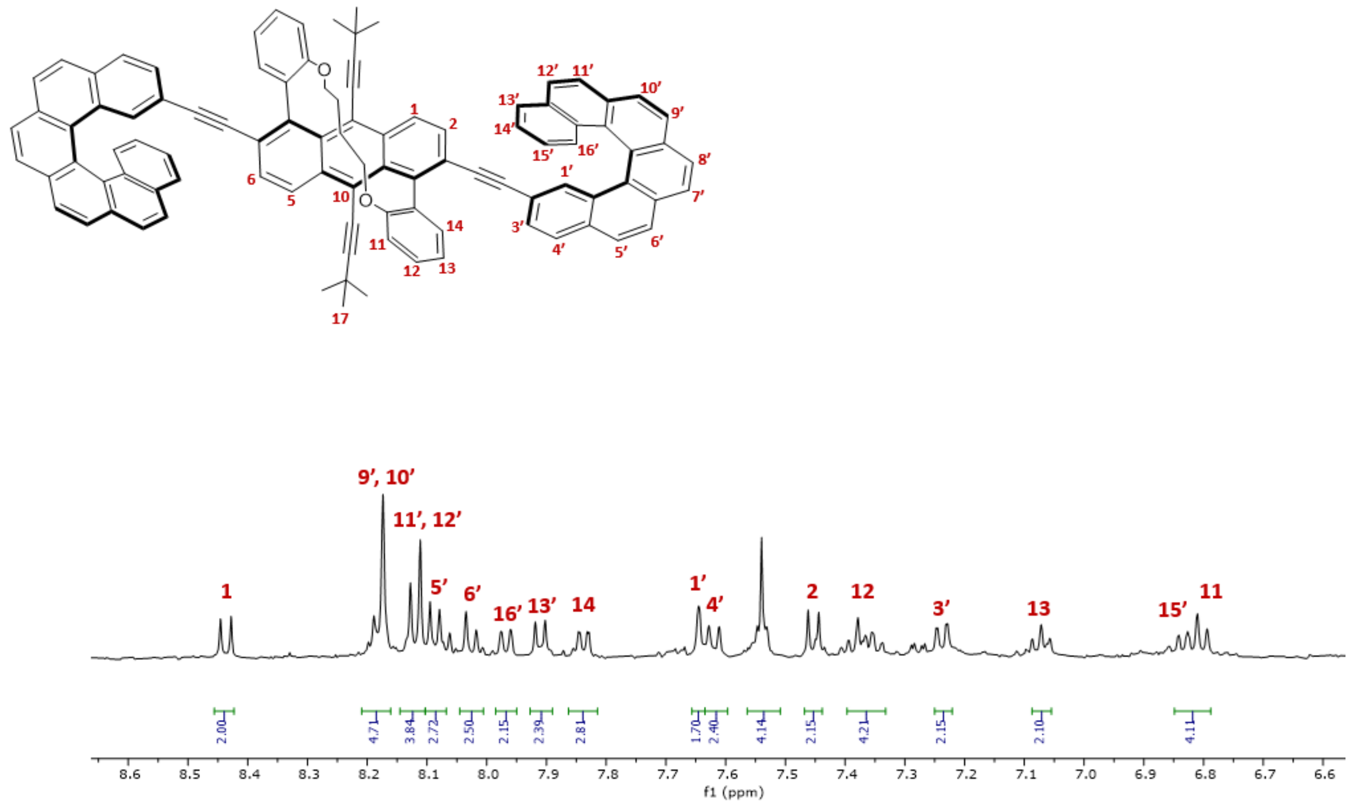

Figure S 79. ^{1}H NMR (500 MHz) of *PPP*-**HT$_4$H** in CD_3NO_2, measured at 298 K.

S7.2 NMR analysis of *PPP*-**HT$_8$H** in different solvents

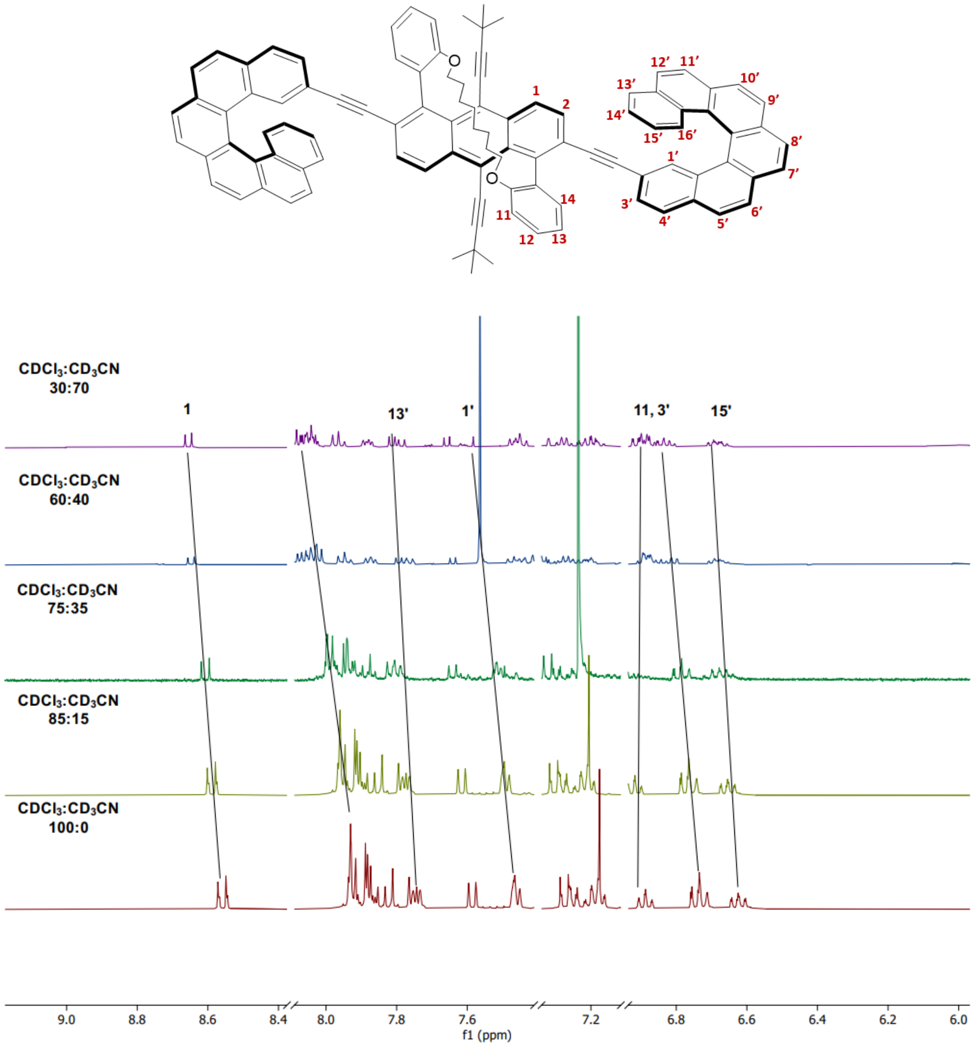

Figure S 80. NMR spectra of *PPP*-**HT$_8$H**. For Full spectra and analysis, see Figure S21.

S8 Supplementary References

[1] M. Jakubec, T. Beránek, P. Jakubík, J. Sýkora, J. Žádný, V. Církva, J. Storch, *J. Org. Chem.* **2018**, *83*, 3607–3616.

[2] A. Bedi, A. Manor Armon, Y. Diskin-Posner, B. Bogosalvsky, O. Gidron, *Nature Communications* **2022**, *13*, 451.

[3] R. Hoffman, *Journal of Magnetic Resonance* **2022**, *335*, 107105.

[4] R. K. Harris, E. D. Becker, S. M. Cabral De Menezes, P. Granger, R. E. Hoffman, K. W. Zilm, *Solid State Nuclear Magnetic Resonance* **2008**, *33*, 41–56.

[5] R. D. Boyer, R. Johnson, K. Krishnamurthy, *Journal of Magnetic Resonance* **2003**, *165*, 253–259.

[6] D. O. Cicero, G. Barbato, R. Bazzo, *Journal of Magnetic Resonance* **2001**, *148*, 209–213.

[7] T. L. Hwang, A. J. Shaka, *J. Am. Chem. Soc.* **1992**, *114*, 3157–3159.

[8] U. Hananel, A. Ben-Moshe, H. Diamant, G. Markovich, *Proceedings of the National Academy of Sciences* **2019**, *116*, 11159–11164.

[9] *SMART-NT V5.6, BRUKER AXS GMBH, D-76181 Karlsruhe, Germany, 2002*, **n.d.**

[10] *SAINT-NT V5.0, BRUKER AXS GMBH, D-76181 Karlsruhe, Germany, 2002*, **n.d.**

[11] *SHELXTL-NT V6.1, BRUKER AXS GMBH, D-76181 Karlsruhe, Germany, 2002*, **n.d.**

[12] J.-L. Calais, *International Journal of Quantum Chemistry* **1993**, *47*, 101–101.

[13] A. D. Becke, *J. Chem. Phys.* **1993**, *98*, 5648–5652.

[14] In *A Chemist's Guide to Density Functional Theory*, **2001**, pp. 33–40.
